# Selecting suitable reference genes for qPCR normalization: a comprehensive analysis in MCF-7 breast cancer cell line

**DOI:** 10.1186/s12860-020-00313-x

**Published:** 2020-09-25

**Authors:** Nityanand Jain, Dina Nitisa, Valdis Pirsko, Inese Cakstina

**Affiliations:** Laboratory of Molecular Genetics, Institute of Oncology, Riga Stradiņš University, Dzirciema street 16, Riga, LV-1007 Latvia

**Keywords:** MCF-7, RT-qPCR, Reference genes, Gene expression, Sub-clones, Breast cancer cell line, Nutrient stress, Culture media

## Abstract

**Background:**

MCF-7 breast cancer cell line is undoubtedly amongst the most extensively studied patient-derived research models, providing pivotal results that have over the decades translated to constantly improving patient care. Many research groups, have previously identified suitable reference genes for qPCR normalization in MCF-7 cell line. However, over the course of identification of suitable reference genes, a comparative analysis comprising these genes together in a single study has not been reported. Furthermore, the expression dynamics of these reference genes within sub-clones cultured over multiple passages (p) has attracted limited attention from research groups. Therefore, we investigated the expression dynamics of 12 previously suggested reference genes within two sub-clones (culture A1 and A2) cultured identically over multiple passages. Additionally, the effect of nutrient stress on reference gene expression was examined to postulate an evidence-based recommendation of the least variable reference genes that could be employed in future gene expression studies.

**Results:**

The analysis revealed the presence of differential reference gene expression within the sub-clones of MCF-7. In culture A1, *GAPDH-CCSER2* were identified as the least variable reference genes while for culture A2, *GAPDH-RNA28S* were identified. However, upon validation using genes of interest, both these pairs were found to be unsuitable control pairs. Normalization of *AURKA* and *KRT19* with triplet pair *GAPDH-CCSER2-PCBP1* yielded successful results. The triplet also proved its capability to handle variations arising from nutrient stress.

**Conclusions:**

The variance in expression behavior amongst sub-clones highlights the potential need for exercising caution while selecting reference genes for MCF-7. *GAPDH-CCSER2-PCBP1* triplet offers a reliable alternative to otherwise traditionally used internal controls for optimizing intra- and inter-assay gene expression differences. Furthermore, we suggest avoiding the use of *ACTB, GAPDH* and *PGK1* as single internal controls.

## Background

MCF-7 (Michigan Cancer Foundation 7) is one of the most commonly used patient derived breast cancer cell line. Established in 1970 by researcher Herbert D. Soule at the Michigan Cancer Foundation, the cell line is derived from the pleural effusion and chest wall nodule showing metastasis of breast adenocarcinoma. The number 7 in MCF-7 represents Soule’s seventh attempt in which he succeeded in generating a cancer cell line [1]. The universal adoption of the cell line is evident from a simple PubMed search (Search word: MCF-7) which retrieves about 39,000 citations (from 1973 to March 2020) with about 900 citations being reported in the first three months of 2020 alone.

In 2013, an updated approach based on IHC (immuno-histochemistry) markers was introduced by St. Gallen International Expert Consensus on Primary Therapy of Early Breast Cancer to determine subtypes of breast cancers [2]. Accordingly, Luminal A subtype tumors were defined as Estrogen Receptor positive (ER+), Progesterone Receptor (PR) ≥ 20%, HER2 negative, Ki67 proliferation marker < 14% and if available, low recurrence risk tumors based on gene-based assays [2]. MCF-7 neoplastic cells were found to be positive for both Estrogen (ER) and Progesterone (PR) receptors along with having low metastatic activity and hence, fulfilled the criteria to be classified as Luminal A molecular subtype tumor cell line [3].

Cell based assays (cell lines) such as MCF-7 represents techniques that can provide more biologically meaningful information than simplified biochemical assays [4]. The key reasons for their universal adoption are lower operational costs and the ease of operation in terms of preparing and observing the cells. Furthermore, they represent an unlimited self-replicating source that can be grown in almost infinite quantities [5] yielding unlimited amounts of DNA/RNA that enables studies related to validation and downstream functional analysis.

However, MCF-7 cell line, like other cell lines is prone to certain disadvantages. It is vulnerable to genotypic and phenotypic drift during its long-term culturing [5]. This is of profound concern since the cell line has been deposited in cell banks for many years now and has risked and in some certain cases caused arising of subpopulations within the cell line. Subpopulations can cause phenotypic changes over time with the selection of specific, more rapidly growing clones within a population, as demonstrated by Osborne et al. [6] and Resnicoff et al. [7] in 1987.

For decades, extensive evidence that MCF-7 cells showed clonal variations have been reported, depicting differences in phenotypic traits such as estrogen/progesterone responsiveness, epidermal growth factor (EGF) expression or the ability to form tumors in syngeneic mice [8]. Also, genetic variability in the sublines and sub-clones of MCF-7 cell line on karyotypic and chromosomal levels have been demonstrated by various researchers [9–15]. Finally, in 2016 Kleensang et al., illustrated variations among MCF-7 cell line obtained from the same cell bank in the same batch [16]. Presence of such heterogeneity bolsters the need for validating and cross-examining the genetic variability as well as reference gene expression in the cell line.

A widely accepted technique for validation of gene expression is the Reverse Transcription – quantitative Polymerase Chain Reaction (RT-qPCR). It is a simple, highly sensitive, reproducible, and high yielding throughput technique that can confirm gene expression differences and measure transcript abundances [17, 18]. Nevertheless, the results obtained from RT- qPCR still need to be normalized against another data set or set references to correct for any sampling noise, such as differences in the amount of starting material, in order to estimate results accurately [19].

These reference genes (previously housekeeping genes) are expressed constitutively and are required for the maintenance of basal cellular functions. In general practice, it is presumed that the endogenous reference gene represents an ideal gene that is sufficiently abundant and has stable expression across different tissues and cell lines under different experimental conditions [20]. In addition, it is assumed that the expression levels remain same amongst biological replicate cell cultures over successive passages. However, some studies suggest that the expression of these reference genes may not be as uniform as previously thought and may also fluctuate significantly under different experimental conditions [21, 22]. Hence, it becomes imperative to validate reference genes before their use in a study as using a non-validated reference gene could lead to misleading interpretations arising from inaccurate results [20, 23].

As pointed out in the MIQE guidelines [24], normalization against a single reference gene is not recommended unless clear evidence of its uniform expression dynamics is described for the specific experimental conditions. Over the previous two decades, many studies have been undertaken identifying new stable reference genes for MCF-7 cell line or breast cancer as a whole [25–32].

However, these studies didn’t undertake a detailed analysis validating the reference gene expression over multiple passages and/or, within sub-clones (biological replicate cultures). This gap in validation leaves us with a cause of concern especially, if such cell lines are to be regarded as valid and suitable models for evaluating the behavior and development of breast cancer and validating their likely response to potential new drugs and therapies.

The present study, therefore, aims to fill the void by investigating the gene expression of twelve reference genes that were previously identified as stable genes by various studies [25–28, 33–37] for MCF-7 cell line, but were not accounted and studied together and therefore, were unable to make an evidence supported recommendation of reference genes to be used for normalization of the gene of interest in MCF-7 cell line cultured routinely as well in nutrient stress conditions. The present study investigated the differential reference gene expression in two sub-clones (culture A1 and A2) cultured identically over multiple passages (p). Additionally, the effect of nutrient stress on reference gene expression was examined to further bolster the selection of reference genes.

## Results

### Curation of the dataset and descriptive analysis

A total of three lysates were collected from each passage from both MCF-7 cultures A1 and A2. Only two lysates were evaluated for passage 28 (p28) and passage 31 (p31) from culture A1. Amplification for each of the 12 reference genes produced a dataset with 900 Cq values. As shown in Table 1, *RNA18S* showed the highest expression in both cultures A1 and A2 (Cq mean = 7.93 and 8.26 respectively), closely followed by *RNA28S* (Cq mean = 8.27 and 8.35 respectively). Both genes showed high amplification levels, appearing close to seven cycles earlier than any other gene in both cultures (*ACTB* Cq mean = 15.98 and 16.11 respectively). *CCSER2* presented the least expression levels in both cultures (Cq mean = 26.58 and 26.56 respectively) while *GAPDH* showed the least standard deviation (S.D = 0.30) in culture A1. *ACTB* showed the least standard deviation (S.D = 0.32) in culture A2. The largest variation between Cq values was shown by *HNRNPL* (S.D = 0.35) in culture A1 and *PGK1* (S.D = 0.61) in culture A2.

**Table 1:** Descriptive Statistics of the Reference Genes Cq (Quantification Cycle) Values

Gene	N ^a^		Mean Cq ± S.D ^b^		Minimum Cq		Maximum Cq	
	A1	A2	A1	A2	A1	A2	A1	A2
***ACTB***	30	45	15.98 ± 0.26	16.11 ± 0.32	15.38	15.53	16.45	16.62
***GAPDH***	30	45	17.13 ± 0.17	17.07 ± 0.39	16.78	16.43	17.52	17.67
***RPL13A***	30	45	21.03 ± 0.29	20.74 ± 0.43	20.71	20.14	21.64	21.70
***PGK1***	30	45	21.08 ± 0.22	21.09 ± 0.61	20.79	20.29	21.76	22.11
***HSPCB***	30	45	20.40 ± 0.29	20.42 ± 0.51	20.02	19.70	20.98	21.77
***RNA28S***	30	45	8.27 ± 0.23	8.35 ± 0.40	7.61	7.83	8.64	9.58
***RNA18S***	30	45	7.93 ± 0.27	8.26 ± 0.49	7.45	7.58	8.47	9.83
***PUM1***	30	45	23.14 ± 0.23	23.08 ± 0.39	22.73	22.35	23.68	24.23
***CCSER2***	30	45	26.58 ± 0.21	26.56 ± 0.39	26.04	26.03	26.95	27.32
***HNRNPL***	30	45	22.73 ± 0.35	22.91 ± 0.56	22.11	21.97	23.52	23.99
***PCBP1***	30	45	22.13 ± 0.22	22.17 ± 0.37	21.78	21.65	22.76	22.94
***SF3A1***	30	45	23.39 ± 0.34	23.51 ± 0.43	22.99	22.78	24.28	24.68

^a^ N – Total number of Cq values from all triplicates of three lysates. ^b^ S.D – Standard Deviation

### Coefficient of variation (CV%)

As shown in Table 2, across both cultures, the CV% was in the range of 14.89 to 40.11% indicating that all the candidate reference genes were characterized by stable expression. The CV% analysis revealed that *PCBP1* was the only gene to be in the top three stable genes (CV% = 14.89% (A1) and 24.90% (A2)) in both the cultures. Apart from *PCBP1*, the other two top stable genes in culture A1 were *GAPDH* (CV% = 12.35%) and *PGK1* (CV% = 14.86%), while in culture A2 those were *ACTB* (CV% = 22.98%) and *RNA28S* (CV% = 24.52%). In stark contrast, *PGK1* in culture A2 was the least stable gene (CV = 40.11%) showing variations in gene expression between the two cultures.

**Table 2:** Mean 2^-Cq^, Standard Deviation (S.D) 2^-Cq^ and CV% of the candidate reference genes

Candidate Reference Gene	MCF-7 Replicate Culture	Mean 2^**-Cq**^	S.D 2^**-Cq**^	CV (S.D/Mean)
***ACTB***	**A1**	1.6E-05	2.9E-06	18.81%
	**A2**	1.4E-05	3.3E-06	22.98%
***GAPDH***	**A1**	7.0E-06	8.7E-07	12.35%
	**A2**	7.5E-06	2.0E-06	26.84%
***RPL13A***	**A1**	4.7E-07	8.7E-08	18.39%
	**A2**	5.9E-07	1.6E-07	26.71%
***PGK1***	**A1**	4.5E-07	6.7E-08	14.86%
	**A2**	4.9E-07	2.0E-07	40.11%
***HSPCB***	**A1**	7.4E-07	1.4E-07	19.34%
	**A2**	7.5E-07	2.4E-07	31.31%
***RNA28S***	**A1**	3.3E-03	5.8E-04	17.66%
	**A2**	3.1E-03	7.7E-04	24.52%
***RNA18S***	**A1**	4.2E-03	8.0E-04	19.17%
	**A2**	3.4E-03	9.9E-04	28.97%
***PUM1***	**A1**	1.1E-07	1.7E-08	15.65%
	**A2**	1.2E-07	2.9E-08	25.03%
***CCSER2***	**A1**	1.0E-08	1.6E-09	15.55%
	**A2**	1.0E-08	2.7E-09	25.65%
***HNRNPL***	**A1**	1.5E-07	3.5E-08	23.70%
	**A2**	1.4E-07	5.2E-08	38.21%
***PCBP1***	**A1**	2.2E-07	3.3E-08	14.89%
	**A2**	2.2E-07	5.5E-08	24.90%
***SF3A1***	**A1**	9.3E-08	2.0E-08	21.20%
	**A2**	8.7E-08	2.4E-08	27.76%

### Relative mean changes in expression profiles of selected candidate reference genes

**Fig. 1:**
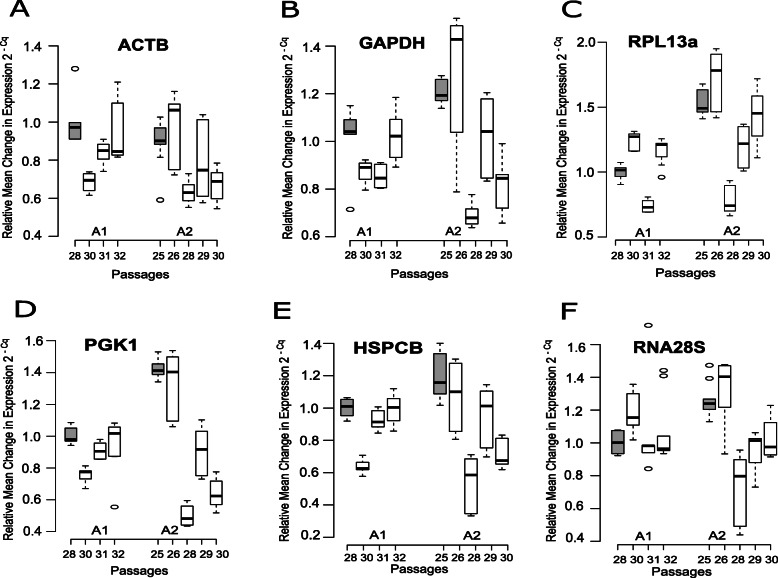
Relative change of expression of the candidate genes (**a**) *ATCB* (**b**) *GAPDH* (**c**) *RPL13A* (**d**) *PGK1* (**e**) *HSPCB* and (**f**) *RNA28S* over successive passages in both cultures A1 and A2. The expression change was measured as 2^-Cq^ with the experimental calibrators marked as gray boxplots (p28 in culture A1 and p25 in culture A2)

**Fig. 2:**
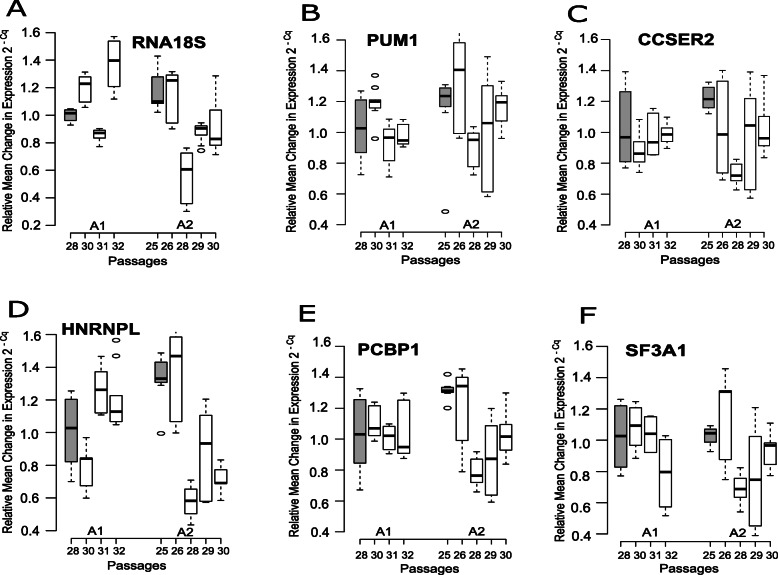
Relative change of expression of the candidate genes (**a**) *RNA18S* (**b**) *PUM1* (**c**) *CCSER2* (**d**) *HNRNPL* (**e**) *PCBP1* and (**f**) *SF3A1* over successive passages in both cultures A1 and A2. The expression change was measured as 2^-Cq^ with the experimental calibrators marked as gray boxplots (p28 in culture A1 and p25 in culture A2)

Relative mean changes in expression profiles were analyzed to study the gene expression stability and variations over successive passages in the both cultures. Passage 28 (p28) was selected as the experimental calibrator for culture A1 while Passage 25 (p25) was selected as the calibrator for culture A2, since they represented the initial investigated passages in both cultures. The fold change was then calculated using 2^-Cq^ with the other passages in both cultures (Figs. 1 and 2).

To determine significant relative expression changes, one-way ANOVA was used (Supplementary Table S1, see Additional file 1). In culture A1, only *CCSER2* (Fig. 2c) and *PCBP1* (Fig. 2e) showed no significant expression changes over successive passages (*ANOVA P* > 0.05). In culture A2, all the genes selected, including *CCSER2* and *PCBP1* showed significant expression changes over successive passages. Both *ACTB* (Fig. 1a) and *GAPDH* (Fig. 1b), along with *PGK1* (Fig. 1d) showed significant expression differences (*ANOVA P* < 0.01) in both cultures over successive passages, providing evidence using these genes as single control endogenous reference genes in MCF-7 cell line should be avoided, if possible.

### NormFinder analysis of MCF-7 sub-clones

**Fig. 3:**
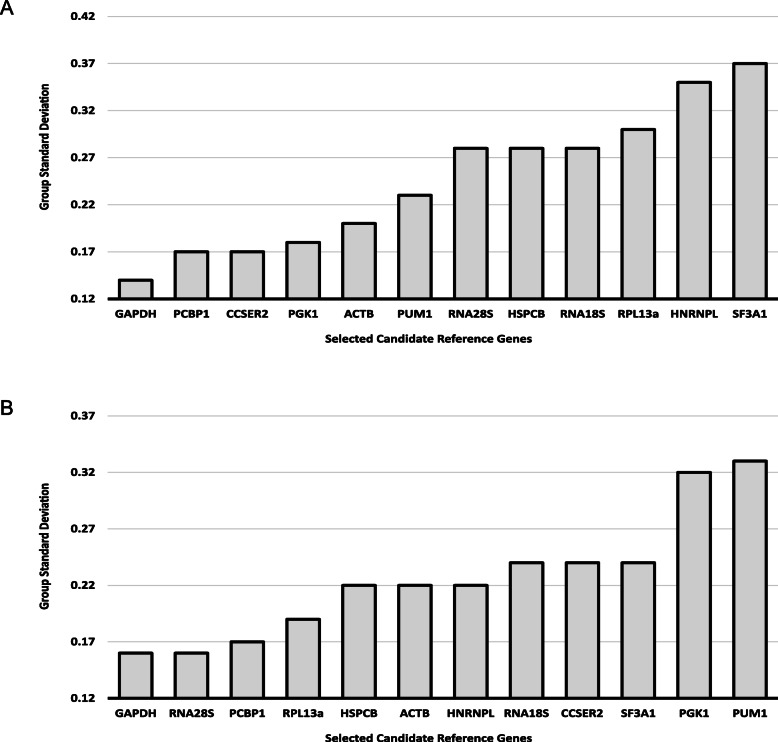
NormFinder analysis for both cultures (**a**) A1 and (**b**) A2 shown as group standard deviations (S.D)

*GAPDH* showed the highest expression stability in both cultures A1 and A2 (S.D = 0.14 and 0.16 respectively) as shown in Fig. 3. It was followed by *PCBP1* and *CCSER2* in culture A1 (S.D = 0.17) while in culture A2, it was matched by *RNA18S* (S.D = 0.16). The algorithm was further used to evaluate the pair of genes best suited to work together as reference genes as shown in Supplementary Table S2 (see Additional file 1) (only the top 10 pairs for both the cultures with their pairwise standard deviations shown). The algorithm ranked *ACTB* and *PUM1* as the most stable pair (S.D = 0.07) in culture A1, while in culture A2, two pairs of genes were equally most stable– *RPL13A – SF3A1* and *GAPDH – SF3A1* (S.D = 0.07).

### geNorm analysis and determination of optimal number of genes needed for normalization of dataset

**Fig. 4:**
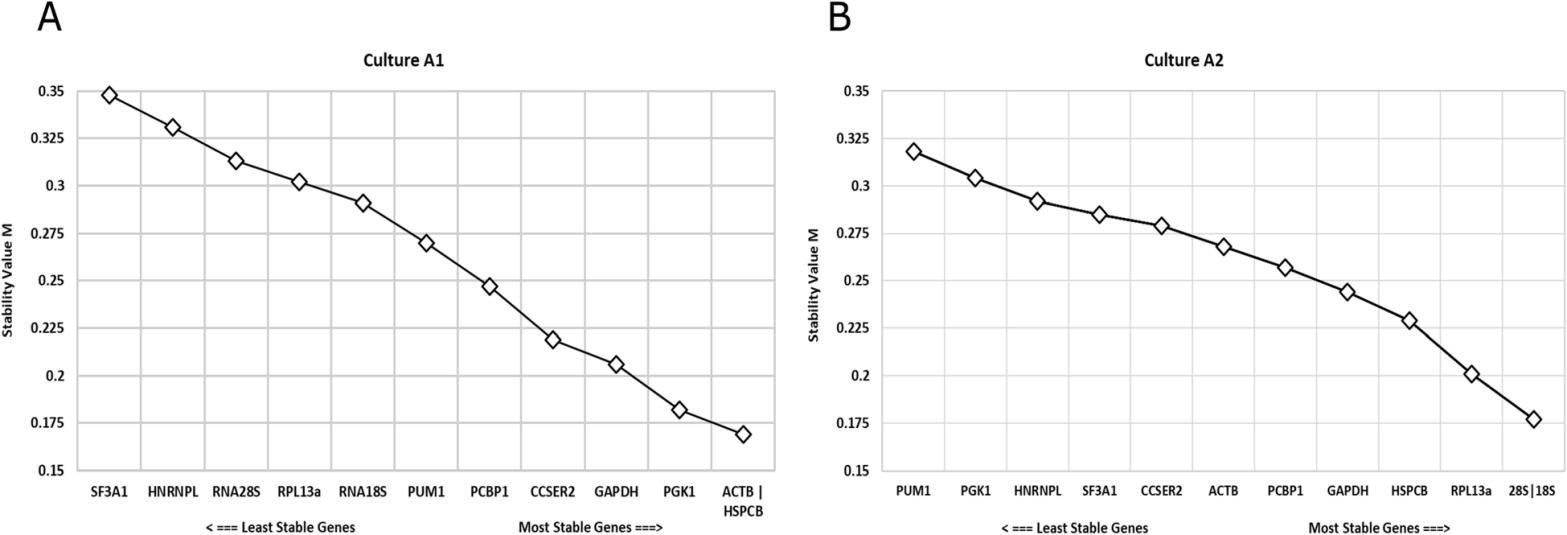
geNorm analysis of the selected candidate genes in both cultures (**a**) A1 and (**b**) A2 indicated as M values (stability values) on the Y-axis and genes on the X-axis. The lower the stability value, the more stable the gene and vice-versa. M value of less than 1 is considered appropriate for candidature as reference gene. 18S and 28S refers to *RNA18S* and *RNA28S* respectively

**Fig. 5:**
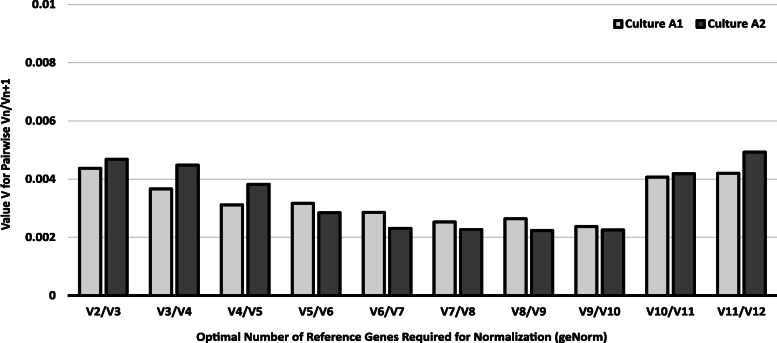
Determination of optimal number of reference genes needed for normalization (geNorm) for both cultures using pairwise V_n/n + 1_ analysis. The recommended cutoff is the lowest V_n/n + 1_ below the threshold of 0.15. In the present study, both the cultures had V_2/3_ less than 0.15, indicating addition of a third reference gene would not affect normalization results

As shown in Fig. 4, there were noticeable differences in the obtained results for both cultures. In culture A1, *ACTB-HSPCB* was the most stable gene (M = 0.169; Fig. 4a) while in culture A2, *RNA18S-RNA28S* tied for the most stable gene (M = 0.177; Fig. 4b). geNorm was further used to determine the optimal number of reference genes needed for an accurate estimation of normalization of expression data, as shown in Fig. 5. For both the cultures A1 and A2, the V_2/3_ (0.00437 and 0.00468 respectively) was less than the recommended cutoff (V_n_/V_n + 1_ < 0.15), indicating the addition of a third reference gene would not make a difference in the normalization results.

### BestKeeper analysis

**Fig. 6:**
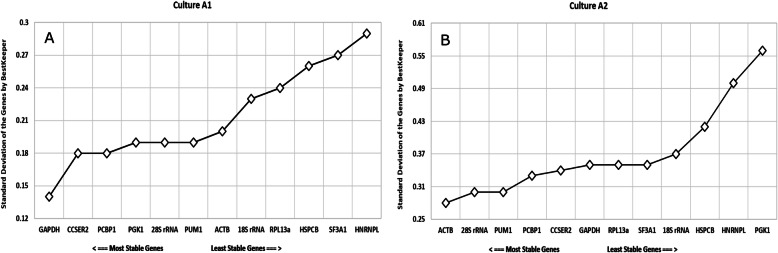
BestKeeper results for standard deviation with crossing points (S.D ± C.P) on the Y axis with the selected candidate genes on the X-axis for (**a**) culture A1 and (**b**) culture A2. The most stable genes are considered to have a S.D as close as possible to 1 with not greater than 1. 18S rRNA and 28S rRNA refers to *RNA18S* and *RNA28S* respectively

In the present study, first we considered the standard deviation with crossing points (C.P) for both cultures (Fig. 6a and b). As seen in the two previous methods (NormFinder and geNorm), BestKeeper also showed deviations in results for both cultures A1 and A2. While *GAPDH* (S.D = 0.14) and *CCSER2* (S.D = 0.18) were the top two genes in culture A1, *ACTB* (S.D = 0.28) and *RNA28S* (S.D = 0.30) were top two genes in culture A2. The standard deviation with changes in x-fold were also examined as shown in Supplementary Table S3 (see Additional file 1). The minimum and maximum fold change (x-fold) obtained from BestKeeper were in concordance with our results from relative mean fold changes (Figs. 1 and 2).

**Fig. 7:**
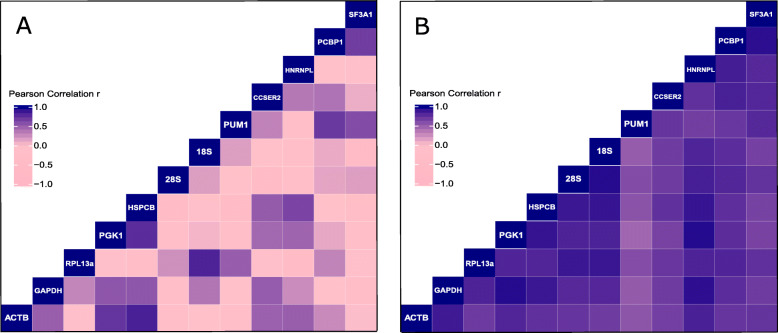
Pearson correlation of the pairwise gene expression stability as obtained from BestKeeper algorithm. The correlation was assessed for all candidate genes in both (**a**) culture A1 and (**b**) culture A2. The darker the blue, the higher the positive correlation between the genes as seen in the legend. 18S and 28S refers to *RNA18S* and *RNA28S* respectively

Finally, coefficient of correlation (r) given as Pearson’s correlation by BestKeeper was evaluated to look for pairwise gene expression stability in both cultures (Fig. 7a and b). The top five gene pairs with high positive coefficient of correlation (r > 0.5) in culture A1 (Fig. 7a) were *HSPCB-ACTB* (r = 0.833), *RPL13A-RNA18S* (r = 0.825), *PGK1-HSPCB* (r = 0.775), *PUM1-PCBP1* (r = 0.704) and *PUM1-SF3A1* (r = 0.598). Similarly, upon analysis of gene pairs in culture A2 (Fig. 7b), the top five gene pairs with r > 0.5 were, *PGK1-HNRNPL* (r = 0.948), *RNA28S- RNA18S* and *PGK1-GAPDH* (both pairs with r = 0.946), *PCBP1-SF3A1* (r = 0.933) and *PGK1-HSPCB* (r = 0.908).

### Pairwise comparative ΔCt analysis of the MCF-7 sub-clones

In culture A1, *GAPDH*, *CCSER2* and *PCBP1* were the top three genes ranked by the algorithm while in culture A2, *RNA28S*, *GAPDH* and *PCBP1* were the top three genes (Supplementary Table S4, see Additional file 1). It is interesting to note that in culture A1, the three top genes ranked by Comparative ΔCt were ranked in the same position and order by BestKeeper (Fig. 6a). In culture A2, only *RNA28S* was reported in the top three by both these algorithms (Supplementary Table S4; Fig. 6a).

### RefFinder analysis

RefFinder is a comprehensive, user friendly, web based tool (https://www.heartcure.com.au/reffinder/) that uses NormFinder, geNorm, BestKeeper and Comparative ΔCt to rank and compare candidate reference genes. The rankings from RefFinder are summarized in Supplementary Table S5 (see Additional file 1). The top three genes in culture A1 were reported to be *GAPDH*, *CCSER2* and *PCBP1*. For culture A2, *RNA28S*, *GAPDH* and *PCBP1* were the top three most stable genes.

### Moving towards selection of reference gene/s based on an integrated approach

Based on the results from above reported algorithms and approaches, we conclude that for culture A1, *GAPDH* and *CCSER2* were suitable reference genes as they were both constantly ranked in top three in NormFinder (Fig. 3a), BestKeeper (Fig. 6a), Comparative ΔCt (Supplementary Table S4, see Additional file 1) and RefFinder (Supplementary Table S5, see Additional file 1). Furthermore, *CCSER2* had no significant relative mean expression change over successive passages (Fig. 2c) while *GAPDH* had shown the least CV% (Table 2) in culture A1. Also, the Pearson Correlation between *GAPDH-CCSER2* (r = 0.515) was significant (*P* = 0.004), thereby indicating their positive correlation (Fig. 7a) and corroborating their selection. Based on a similar approach, for culture A2, *GAPDH* and *RNA28S* were selected as suitable reference genes. Interestingly, to avoid excluding a potential candidate as a reference gene, as a result of the strict selection of only two reference genes, as suggested by geNorm (Fig. 5) an experimental candidate with consistent high rankings in all the algorithms for both cultures was also chosen. *PCBP1* was identified as a strong contender and hence was included in the validation of selected reference genes. *PCBP1* was one of two genes to have no significant relative expression changes in culture A1 (Fig. 2e) and claimed the top spot in NormFinder (Fig. 3), BestKeeper (Fig. 6), Comparative ΔCt (Supplementary Table S4) and RefFinder (Supplementary Table S5). It also consistently ranked in the top five genes as per the geNorm analysis for both cultures (Fig. 4a and b).

### Generation of data for two genes of interest (GOIs) & validation of reference genes using normalization

To further evaluate the four selected reference genes (*GAPDH*, *RNA28S*, *CCSER2* and *PCBP1*) and their utility as reference genes, two simulated datasets were created. The genes simulated were referred to as *Gene of Interests (GOI) 1* and *2.* Both were assigned some random Cq values (triplicates for 3 lysates over 4 passages for culture A1 and over 5 passages for culture A2). The criteria for the assignment of Cq values, as well as the datasets are presented in Supplementary Table S6 and S7 (see Additional file 1).

**Fig. 8:**
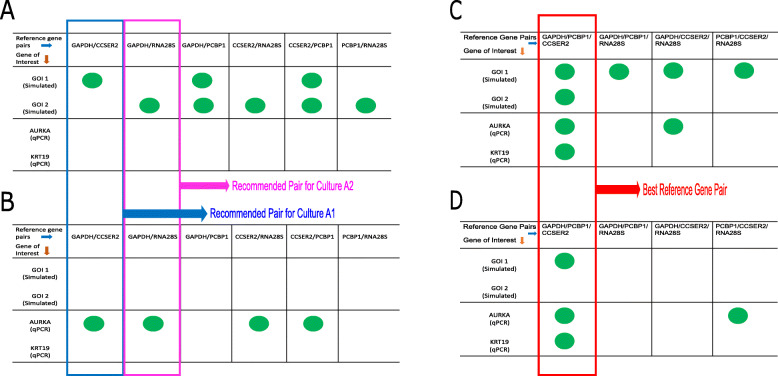
Summary of Normalization of *GOI 1, GOI 2, AURKA* and *KRT19* by different recommended reference genes pairs. Green circles indicate successful normalization of the gene of interest by respective reference gene pair (**a**) Normalization of the four genes of interest by selected reference genes in pairs of 2 in culture A1 (**b**) Normalization of the four genes of interest by selected reference genes in pairs of 2 in culture A2 (**c**) Normalization of the four genes of interest by selected reference genes in pairs of 3 in culture A1. (**d**) Normalization of the four genes of interest by selected reference genes in pairs of 3 in culture A2. Blue box indicates normalization by *GAPDH-CCSER2* pair (recommended pair in culture A1) while Pink box indicates normalization by *GAPDH-RNA28S* pair (recommended pair in culture A2). Red box indicates the best reference gene triplet (GAPDH-CCSER2-PCBP1)

For *GOI 1* in culture A1, as suggested by the algorithms used in this study, the *GAPDH – CCSER2* pair proved its utility when used for normalization of the simulated *GOI 1* (Supplementary Fig. S1A, see Additional file 2 and Fig. 8a). After normalization with two other pairs, namely *GAPDH – PCBP1* and *CCSER2 – PCBP1*, there were no statistically significant fold changes (*ANOVA P* > 0.05), in culture A1, *GOI 1*, as seen in Fig. 8a (also see Supplementary Fig. S1A, Additional file 2). In culture A2, the most stable pair suggested was *GAPDH-RNA28S*, which when used for normalization of *GOI 1* showed statistically significant fold changes (*ANOVA P* < 0.05) for passage 25/28 and passage 25/30. Furthermore, all other reference genes pairs in culture A2 showed statistically significant fold changes when used for normalization (Supplementary Fig. S1B, see Additional file 2 and Fig. 8b).

Normalization was then performed in pairs of three reference genes to investigate whether any pairing can be used for normalization, as shown in Supplementary Table S8 (see Additional file 1) and Fig. 8c. In culture A1, all the different combinations of reference genes possible showed successful normalization while in culture A2, only *GAPDH-PCBP1-CCSER2* triplet achieved successful normalization (Fig. 8d and Supplementary Table S8, Additional file 1).

On similar lines, for *GOI 2* in culture A1, *GAPDH – CCSER2* pair produced statistically significant results in fold change (*ANOVA P* = 0.035) after normalization of *GOI 2*, indicating the pair may not after all be able to handle large differences in Cq values (greater than +/ 0.5 Cq) as seen in Fig. 8a and Supplementary Fig. S2A (see Additional file 2). Interestingly, all the other reference gene pairs proved their utility and produced insignificant changes (*ANOVA P* > 0.05) after normalization of *GOI 2* in culture A1 (Fig. 8a and Supplementary Fig. S2A, see Additional file 2). In this instance, for culture A2, *GAPDH-RNA28S* pair produced statistically significant fold changes (*ANOVA P* = 0.07), indicating it not to be a suitable pair for normalization. For all pairs in culture A2, statistically significant changes were recorded in only one passage, p25/p29, as seen in Fig. 8b (also see Supplementary Fig. S2B, Additional file 2), and hence further normalization with three reference genes was undertaken in a bid for successful normalization.

After performing further normalization of *GOI 2* for cultures A1 and A2 in pairs of three reference genes (Supplementary Table S9, see Additional file 1), our analysis showed no suitable pair of reference genes for *GOI 2* in culture A2 (Fig. 8d) but for culture A1, *GAPDH-PCBP1-CCSER2* was the only triplet to yield successful normalization (Fig. 8c).

### Validation of selected reference genes with normalization of qPCR data

To support and continue perusing the selected reference gene pairs, two genes of interest were also used, namely *AURKA* (Aurora Kinase A) and *KRT19* (Keratin 19). The qPCR was performed, and the dataset was normalized using the reference gene pairs as shown in Supplementary Fig. S3-S4 (see Additional file 2). Statistical significance and methodology were the same as for *GOI 1* and *GOI 2*. The Cq range for *AURKA* was from 23.36 (min) to 24.51 (max) with mean Cq of 23.92 ± 0.39 for culture A1 while for culture A2, the Cq range was from 23.19 (min) to 24.32 (max) with mean Cq of 23.74 ± 0.31. The Cq range for *KRT19* was from 18.90 (min) to 20.13 (max) with mean Cq of 19.29 ± 0.29 for culture A1 while for culture A2, the Cq range was from 18.65 (min) to 21.19 (max) with mean Cq of 19.96 ± 0.77.

Upon normalization of *AURKA*, none of the gene pairs in culture A1 were able to normalize *AURKA* adequately (*P* < 0.05) as seen in Fig. 8a and Supplementary Fig. S3A (see Additional file 2). In culture A2, however, four reference gene pairs were able to normalize the *AURKA* (Fig. 8b; Supplementary Fig. S3B, see Additional file 2). For *KRT19*, in both cultures A1 and A2, no gene pair could yield successful normalization (Fig. 8a and b; Supplementary Fig. S4A and S4B, see Additional file 2). Again, further investigations were done using genes in triplets.

For *AURKA*, in culture A1, *GAPDH-PCBP1-CCSER2* and *GAPDH-CCSER2-RNA28S* yielded successful normalization (Fig. 8c and Supplementary Table S10, see Additional file 1). In culture A2, *GAPDH-PCBP1-CCSER2* and *PCBP1-CCSER2-RNA28S* pairs showed adequate normalization of AURKA (Fig. 8d and Supplementary Table S10, see Additional file 1). For *KRT19*, in both cultures A1 and A2, only *GAPDH-PCBP1-CCSER2* triplet showed successful normalization (Fig. 8c and d; Supplementary Table S11, see Additional file 1).

### Combined analysis of Dataset from MCF-7 sub-clones A1 and A2: analysis of MCF-7 cell line

From the extensive analysis provided above, the biological replicate cultures of MCF-7 (sub-clones) does not necessarily depict similar phenotypic characteristics and gene expression and hence, the reproducibility of the results among sub-clones may not be 100% efficient. Therefore, to further investigate the reference gene/s which show least variance overall in the MCF-7 cell line, the dataset from both sub-clones A1 and A2 were combined and analyzed. CV% was determined to evaluate the stability of reference genes (Table 3). As before, comprehensive analysis was conducted for the combined dataset using NormFinder, geNorm, BestKeeper, Comparative ΔCt and RefFinder. The gene rankings were determined using each of these algorithms as summarized in Table 3.

**Table 3:** Comprehensive analysis of candidate reference gene ranking with dataset from both culture A1 and A2 combined

**CV%**			**NormFinder**			**geNorm**		
**Candidate Gene**	**CV%**	**Rank**	**Candidate Gene**	**Group S.D**	**Rank**	**Candidate Gene**	**Stability value M**	**Rank**
*PCBP1*	22.13	1	*GAPDH*	0.15	1	*CCSER2*	0.23	1
*ACTB*	22.28	2	*PCBP1*	0.16	2	*PCBP1*	0.23	1
*RNA28S*	22.74	3	*RNA28S*	0.21	3	*GAPDH*	0.25	2
*PUM1*	22.99	4	*CCSER2*	0.21	3	*ACTB*	0.26	3
*CCSER2*	23.17	5	*ACTB*	0.21	3	*HSPCB*	0.28	4
*GAPDH*	23.62	6	*HSPCB*	0.24	4	*RNA28S*	0.29	5
*SF3A1*	26.49	7	*PGK1*	0.27	5	*PGK1*	0.30	6
*RNA18S*	27.97	8	*HNRNPL*	0.28	6	*HNRNPL*	0.31	7
*RPL13A*	28.11	9	*PUM1*	0.29	7	*SF3A1*	0.32	8
*HSPCB*	28.65	10	*RPL13A*	0.29	7	*PUM1*	0.33	9
*HNRNPL*	35.10	11	*RNA18S*	0.30	8	*RPL13A*	0.34	10
*PGK1*	35.61	12	*SF3A1*	0.30	8	*RNA18S*	0.35	11
**BestKeeper**			**Comparative ΔCt**			**RefFinder**		
**Candidate Gene**	**S.D**	**Rank**	**Candidate Gene**	**Average S.D**	**Rank**	**Candidate Gene**	**Geomean**	**Rank**
*RNA28S*	0.25	1	*GAPDH*	0.30	1	*GAPDH*	1.97	1
*PUM1*	0.26	2	*PCBP1*	0.30	1	*PCBP1*	2.00	2
*ACTB*	0.26	3	*CCSER2*	0.33	2	*CCSER2*	2.91	3
*PCBP1*	0.27	4	*RNA28S*	0.33	2	*RNA28S*	2.91	3
*GAPDH*	0.27	4	*ACTB*	0.33	2	*ACTB*	4.16	4
*CCSER2*	0.28	5	*HSPCB*	0.34	3	*PUM1*	6.34	5
*SF3A1*	0.32	6	*PGK1*	0.36	4	*HSPCB*	6.51	6
*RPL13A*	0.33	7	*HNRNPL*	0.37	5	*PGK1*	7.84	7
*RNA18S*	0.34	8	*PUM1*	0.38	6	*HNRNPL*	8.85	8
*HSPCB*	0.35	9	*SF3A1*	0.38	6	*SF3A1*	9.32	9
*PGK1*	0.41	10	*RPL13A*	0.38	6	*RPL13A*	9.92	10
*HNRNPL*	0.43	11	*RNA18S*	0.38	6	*RNA18S*	10.93	11

As reported in Table 3, CV% analysis revealed that the least variable gene is *PCBP1* (CV% = 22.13%) closely followed by *ACTB* and *RNA28S* (CV% = 22.28 and 22.74% respectively). NormFinder, Comparative ΔCt and RefFinder had almost identical rankings where *GAPDH* and *PCBP1* were consistently reported as the most stable genes. Furthermore, in Comparative ΔCt and RefFinder, both *CCSER2* and *RNA28S* were shown to be the next most stable genes. In contrast, NormFinder ranked *RNA28S* before *CCSER2*, however both were reported in top four genes. geNorm on the other hand ranked *CCSER2 – PCBP1* duo as the most stable genes (M value = 0.23). It further ranked *GAPDH* as the third most stable gene. geNorm was further used to calculate V_n_/V_n + 1_ to estimate the number of reference genes required for normalization of GOIs. geNorm reported that two reference genes should be used (V_2/3_ = 0.005 is less than the cutoff of 0.15). BestKeeper, however ranked *RNA28S* and *PUM1* as the two most stable genes whilst ranking *PCBP1* as the fourth most stable gene.

Finally, BestKeeper was used to report the Pearson’s correlation (r) among genes. A high positive correlation was reported for *PCBP1-CCSER* (r = 0.757). A moderate positive correlation was reported for *PCBP1-GAPDH* and *CCSER-GAPDH* pairs (r = 0.643 and 0.666 respectively). Whilst *RNA28S* showed moderate positive correlation with *GAPDH* (r = 0.623) and *PCBP1* (r = 0.686), it portrayed a low positive correlation with *CCSER2* (r = 0.496). Two genes that were constantly ranked as the least stable (most variable) were *RNA18S* and *RPL13A* except by CV% and BestKeeper.

### Transcriptomic analysis of the reference genes from TCGA database

The TCGA (The Cancer Genome Atlas) database was used for transcriptomic validation of the expression of the reference genes analysed in the study. Based on the *PAM50 BRCA* subtype classifications, data of only Luminal A subtype tumor patients were analysed (others like Normal, Luminal B, Undertermined, Basal etc. sub-types were removed). In the dataset, selected reference genes were looked for. Normalised gene expression data was available for 9 of the 12 reference genes (data not available for *ACTB*, *RNA18S* and *RNA28S*) as presented in Supplementary Table S12 (see Additional file 1). For better data visualization and understanding gene expression of the reference genes, the *“normalized_count”* was converted to the log scale using log2 (normalized_count + 1) as presented in Supplementary Fig. S5 (see Additional file 2). *CCSER2* is the most stable gene (from our selected reference genes) identified in the database for Luminal A sub-type breast cancer. Furthermore, *PCBP1* was found to be quite stably expressed. Interestingly, *GAPDH* which was identified as the most stable gene in both cultures, was identified as the least stable gene in the transcriptomic analysis.

To evaluate the TPM (transcripts per million), the *TCGAbiolinks* was used again to retrieve the clinical, morphological and expression data of Luminal A sub-type breast cancer patients. In case of TPM, the data was available for 10 of the 12 selected candidate reference genes (except for *RNA28S* and *RNA18S*) as shown in Supplementary Table S13 (see Additional file 1). For better visualization and data analysis, the TPM values were also converted to logarithmic scale using log2(TPM).

All the selected reference genes from the database showed low variance with a S.D. (log2 TPM) of less than 1. Also, they all showed medium to high expression level since the mean (log2 TPM) was greater than 5 for all genes (except for *CCSER2*, which showed lower expression levels). The log2 (TPM) ranges of all the genes are shown in Supplementary Fig. S6 (see Additional file 2).

### Relationship between Cq values from RT-qPCR and log2 (TPM) of TCGA RNA-Seq

To facilitate an estimation of the relationship between the Cq values obtained from RT-qPCR for both cultures A1 and A2 and the log2 (TPM) values obtained from TCGA database, a correlation analysis was performed, as seen in Supplementary Fig. S7 (see Additional file 2). Although there exists no formal relationship or formula between Cq and Log2 (TPM), the formulas (y intercept and R^2^) presented in Supplementary Fig. S7, provide an estimation of the Ct for each reference gene prior to RT-qPCR experiments based on RNA-Seq data of Luminal A sub-type breast cancer which may be extended to other Luminal A cell lines. Pearson’s correlation was also calculated between Cq values from both cultures A1 and A2 (and combined mentioned as MCF-7) and log2 (TPM) as seen in Table 4. Statistical significance was set at *P* < 0.05. No significant correlation was found in any of the gene in both cultures when Cq values were compared with Log2 (TPM) RNA-Seq values.

**Table 4:** Pearson’s Correlation between Cq values from culture A1 and A2 from RT-qPCR and log2 (TPM)

Candidate Reference Gene	Pearson Correlation r (Culture A1 vs log2 TPM)	Pearson Correlation r (Culture A2 vs log2 TPM)	Pearson Correlation r (MCF-7 vs log2 TPM)
***ACTB***	0.215 (*P* = 0.253)	- 0.028 (*P* = 0.852)	0.115 (*P* = 0.323)
***GAPDH***	- 0.316 (*P* = 0.088)	- 0.315 (*P* = 0.837)	0.078 (*P* = 0.504)
***RPL13A***	- 0.018 (*P* = 0.923)	- 0.132 (*P* = 0.385)	- 0.071 (*P* = 0.272)
***PGK1***	- 0.308 (*P* = 0.097)	0.002 (*P* = 0.988)	0.028 (*P* = 0.812)
***HSPCB***	- 0.053 (*P* = 0.780)	- 0.013 (*P* = 0.927)	- 0.004 (*P* = 0.970)
***PUM1***	0.229 (*P* = 0.221)	0.119 (*P* = 0.432)	0.083 (*P* = 0.478)
***CCSER2***	- 0.015 (*P* = 0.936)	- 0.049 (*P* = 0.747)	- 0.029 (*P* = 0.798)
***HNRNPL***	- 0.150 (*P* = 0.428)	0.079 (*P* = 0.601)	0.069 (*P* = 0.554)
***PCBP1***	0.061 (*P* = 0.745)	- 0.126 (*P* = 0.407)	- 0.045 (*P* = 0.702)
***SF3A1***	0.310 (*P* = 0.095)	0.261 (*P* = 0.082)	0.132 (*P* = 0.256)

### Nutrient stress – fold changes of reference genes

To demonstrate and validate the reference gene triplet (*GAPDH-CCSER2-PCBP1*), samples from MCF-7 cells cultured in four different culturing conditions were analyzed. The cell cultures were named as B5, D5, E5 and R5. We first calculated the fold change in expression of reference genes in control (cultures A1 and A2) versus nutrient stress (cultures B5, D5, E5 and R5). Additionally, we examined the potential differences in fold change between control cultures. We selected p28 from culture A1 and p25 from culture A2 for comparisons of fold change as shown in Supplementary Table S14 (see Additional file 1). A well defined threshold for acceptable range of tolerable fold change does not exist, however, a commonly suggested arbitary limit of ≥ 2x fold change was used in the present study to decide whether or not the reference gene was a good internal control [38]. Accordingly, our analysis revealed that there was more than 2x fold change for *ACTB* (all nutrient stress cultures), *PGK1* (B5, D5 and E5), *GAPDH* (E5) and *HNRNPL* (E5) when compared with control MCF-7 cell line (A1 + A2).

### Nutrient stress – expression differences of GOIs when normalized using GAPDH-CCSER2-PCBP1

**Fig. 9:**
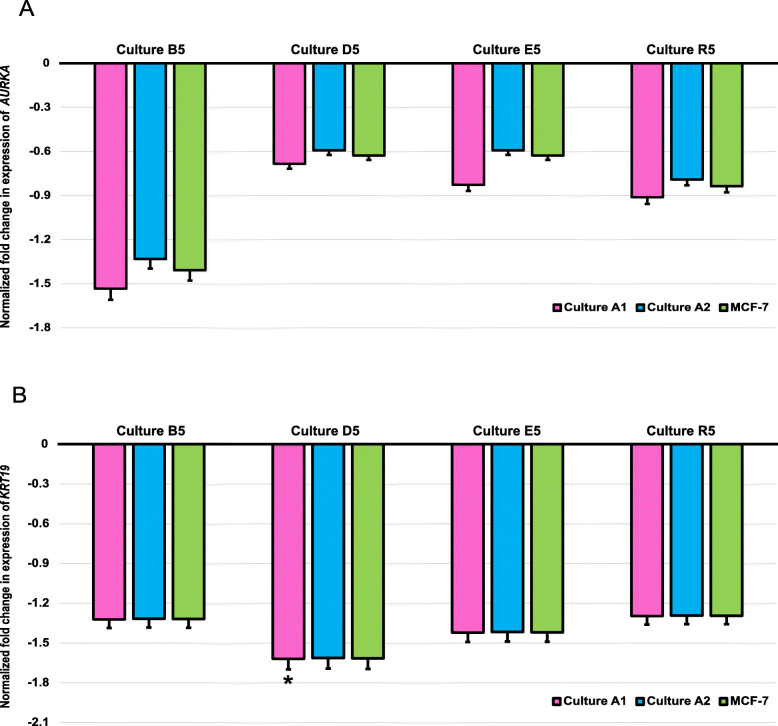
Normalized fold change in expression of genes of interest (**a**) *AURKA* and (**b**) *KRT19* in stress cultures (B5, D5, E5 and R5) when compared with control cultures (A1 and A2). Pink boxes indicate normalized fold change in expression when compared with p28 (culture A1); Blue boxes indicate normalized fold change in expression when compared with p25 (culture A2) and Green boxes indicate normalized fold change in expression when compared with control MCF-7 cell line (p28 + p25 from A1 and A2). * Indicates significant fold change (*P* < 0.005) as calculated using Mann-Whitney *U* test

Finally, the normalized fold change in expression for *AURKA* and *KRT19* was evaluated in all four nutrient stress cultures and normalized against 3 controls – p28 from culture A1, p25 from culture A2 and p25 with p28 for overall MCF-7 cell line as shown in Fig. 9. The triplet successfully normalized the expression fold change for both genes of interest in all stress cultures except for B5/p28 (culture A1), where, although the fold change was < 2, it was found to be statistically significant (*P* = 0.045). Fold change due to nutrient stress varied from − 0.593 (D5 and E5) to − 1.533 (B5) for *AURKA* and from − 1.292 (R5) to − 1.615 (D5) for *KRT19*.

## Discussion

The human breast adenocarcinoma cell line, MCF-7 has been a standard model among researchers for about five decades, serving as a laboratory tool for in vitro studies as well as a model for investigation of key cancer driven processes that directly impact patient care and treatment plan [39]. Despite publication of extensive evidence [9–15], the genetic and phenotypic variance in MCF-7 sub-clones and subpopulations is often not accounted for in the laboratory protocols and is hence overlooked. The main reason for this oversight usually stems from assumptions that by using cells obtained from same batch and same cell bank and by standardizing protocols and limiting the number of passages, laboratories can ensure that their sub-clones will “behave” with sufficient stability and reproducibility [16].

Furthermore, laboratories can argue that by adhering strictly to the recommendations from Good Cell Culture Practice (GCCP) [40] and employing SNP/STR cell authentication techniques, one can reproduce their results with MCF-7 sub-clones. However, this may not be specifically related to the MCF-7 cells. In fact, MCF-7 estrogen disruptor assay failed to get international validation in 2016 by US NICEATM (US National Toxicology Program Interagency Center for the Evaluation of Alternative Toxicological Methods). The main reason cited for failure was centered on concerns regarding the inter-laboratory reproducibility of results [41].

The accuracy in reproducibility of results with MCF-7 cells has been questioned previously [42], but no substantial proof that dealt with variations in reference gene expression tendencies was reported. The present study addresses this issue for the first time and reports evidence of a heterogenous reference gene expression in sub-clones of MCF-7 cell line which are cultured identically. Therefore, it is pivotal to validate and test the reference genes before their use amongst the sub-clones in order to avoid inaccurate normalization of genes of interest.

Our analysis revealed that among the two biological replicate cultures (sub-clones), there are stark differences in the expression patterns and tendencies of the endogenous reference genes. *GAPDH- CCSER2* were identified as potential genes for culture A1, while *GAPDH-RNA28S* were identified for culture A2 using various algorithms. However, when they were employed for cross-normalization of genes of interests in both cultures, both gene pairs were unable to provide adequate results (Fig. 8). The addition of a third gene *PCBP1* to *GAPDH-CCSER2* pair helped to yield successful normalization for all 4 genes of interest and in both cultures A1 and A2 (except for *GOI 2* in Culture A2; this can be attributed most likely to limitation of *GOI 2* as being a simulated gene).

The TCGA (The Cancer Genome Atlas Program) represents a joint venture of National Cancer Institute (NCI) and National Human Genome Research Institute (NGGRI) which began in 2006 as a pilot project with three cancer types (lung, ovarian and glioblastoma). This was later expanded to present 33 tumor types encompassing a comprehensive dataset describing the molecular changes that occur in cancer [43]. Two different approaches were employed to analyze the legacy dataset and compare the gene expression, namely normalized_count (file extension rsem.genes.normalized_results) and TPM obtained from scaled_estimate (file extension rsem.genes.results). Both approaches revealed *CCSER2* to be the most stably expressed. The CV% was estimated at 17.49% from TCGA analysis which was between the range of 15.55% (culture A1) and 25.65% (culture A2) obtained from RT-qPCR (Table 2) in present study. *PCBP1* was also expressed quite stably with CV% estimated at 3.96% from TCGA analysis. The RT-qPCR range for *PCBP1* obtained was from 14.89% (culture A1) and 24.90% (culture A2). Finally, CV% for *GAPDH* from TCGA was 6.44% while RT-qPCR Cq values ranged from 12.35% (culture A1) to 26.84% (culture A2) (Table 2).

Whilst TCGA analysis of *CCSER2* and *PCBP1* supported our findings in the present study and bolsters their selection, contrastingly *GAPDH* was revealed to be unstably expressed. It is important to point out that the difference in results could be attributed to various underlying limitations. Firstly, TCGA requires all malignancies in its database to be primary, untreated tumors (except cutaneous melanoma) [44]. Furthermore, all specimens deposited were garnered from available frozen materials, from different institutes, thereby introducing bias in institutional biorepository collections, stemming from institutional research interests, operative protocols, or patient populations [44]. In addition, metastatic diseases or aggressive primary tumors are usually subject to neoadjuvant therapies which make their inclusion in TCGA database difficult because of limited availability of untreated specimens [44]. However, all these limitations should not diminish the fact that TCGA remains one of the richest source of clinical and research importance, especially in developing an integrated picture of commonalities, differences, and emergent themes across tumors.

Use of *GAPDH* as reference gene in qPCR normalization has been a matter of controversy in its own right. *GAPDH* has been shown to have increased expression in cancers from other body regions specially from cervix, prostate, pancreas and lungs [45–48]. Furthermore, it has been reported that *GAPDH* is overexpressed in MCF-7 cells treated with estradiol [49]. Hence, many studies suggest not to use *GAPDH* as a control gene to study breast cancer or they rank *GAPDH* as the least stable gene [25, 32, 33, 49–51]. As reported by Liu et al. [25], almost half of the publications in PubMed database used *GAPDH* as a single reference gene for normalization of gene expression analyses with RT-qPCR. Even with the contradiction regarding consideration of *GAPDH* as a suitable or non-suitable candidate, attention should be paid to its selection based on the experimental conditions and study design [33].

*CCSER2* is described as a “novel housekeeping gene” (nHKG) by Tilli et al. [27], who provided evidence of its use as a reference gene in breast cancer studies. They demonstrated that expression of *CCSER2* is expected to be like the other nHKGs they had identified that could increase the assessment consistency of normalization. Correspondingly, *CCSER2* was ranked highly in culture A1 in our analysis and was confirmed by transcriptomic validation to be the least variable in expression, therefore it can be hence, used for future analysis and experiments. In addition, *PCBP1*, the most trustworthy gene in our analysis with a stable ranking across all platforms shows promising candidature as an endogenous reference gene as reported also by Jo et al. [28].

Another gene that came to light in the analysis for culture A2 was *RNA28S*. The gene has not been reported much in the context for MCF-7 cell line and was added as an in-house suggestion in the present study. However, drawbacks to the use of *RNA18S* or *RNA28S* as reference genes have been reported such as an absence of purified mRNA samples and their high abundance compared to target mRNA transcripts making it difficult to accurately subtract the baseline value in RT-PCR data analysis [20]. Furthermore, the use of these genes as control genes is not suggested due to the imbalance between mRNA and rRNA fractions in these molecules [52]. In addition, it has also been shown that certain drugs and biological factors may also affect the rRNA transcription [53, 54].

*ACTB*, another widely used reference gene, has also been previously verified as a candidate stable reference gene for breast cancer tissue and normal tissues [32, 55]. *ACTB* and *HSPCB* were the best reference genes identified by Liu et al. [25] for ER+ breast cancer cell lines including MCF-7. They further reported that *RNA18S* and *ACTB* were the best pair of genes across all breast cancer cell lines [25]. Despite the widespread acceptance of *ACTB* for normalization among a set of human breast cancer cell lines of increasing metastatic potential, limitations have been reported as well [29]. Jacob et al. [33] identified *HSPCB* as one of the suitable genes across a variety of cancer cell lines including MCF-7. Our analysis revealed contrasting results. *ACTB, HSPCB, RPL13A* and *RNA18S* were ranked outside of the top three in both cultures. Furthermore, *ACTB* and *HSPCB* reported high CV% and showed significant fold changes (2^-Cq^ analysis). The difference between our results and previously reported studies is suggestive of the fact that inter-laboratory replication of results with MCF-7 cell line is not very accurate.

Nonetheless, the authors caution the readers that the results obtained in the present study must be seen in the light of some limitations. Firstly, the reference genes were validated for their expression and utility in in-vitro only and hence further validation is needed to replicate our findings in-vivo, like MCF-7 derived xenograft models. Secondly, the present study tested reference genes in four different nutrient stress conditions, however, other conditions like hypoxia, drug treatment etc. still need to be tested to validate the stability of *GAPDH-CCSER2-PCBP1* triplet. Finally, given the heterogeneity of the MCF-7 cell line, it remains to be tested if the results from our study could be replicated in MCF-7 cell line that has been obtained from a different batch number or source i.e. inter-laboratory validation of the results is still needed.

An interesting observation was made regarding passage p28 from culture A2 (Figs. 1 and 2). Lower Cq values were obtained for all housekeeping genes when compared to other passages in the same culture. A plausible explanation for this observation cannot be provided at this point. Good Cell Culturing Practices (GCCP)  were strictly adhered to and the cell culturing, RNA isolation, quality control using PCR and RT-PCR were all performed by the same single operator to minimize technical errors. Furthermore, the RNA isolation for all passages (and all lysates) for both the cultures was executed in a single batch, on the same day. The same was also true for RT-PCR, thereby effectively minimizing any chance of human error.

A more detailed discussion of the results obtained, and their implications is presented in Additional file 3. The study, together with its results, aims to provide the readers and researchers an evidence-based recommendation of the most suitable reference genes in routinely cultured MCF-7 cell line with extensive research and investigations. Furthermore, we report the possible existence of a heterogenous differential behavior of endogenous reference genes within the sub-clones of the MCF-7 cell line. The authors suggest that more detailed and diverse studies should be undertaken to explore more about the differential expression of the endogenous reference genes. Future studies could be aimed at investigating the reference gene expression amongst other sub-populations of MCF-7 and amongst other breast cancer cell lines.

Lastly, given the widespread use of cancer cell lines not only for basic research but also for drug development and regulatory decision making, ensuring that sub-clones among the cell line are adequately standardized in their expression behavior tends to represent a challenging path forward [16]. A strategy outlined by Tilli et al. [27], whereby experiments are normalized with a panel of reference genes whose expressions have been proven to be as minimally variable as possible and as robust as possible under varying conditions gains our support as well. Although geNorm recommended to use two reference genes, we would recommend that three reference genes should be employed to better overcome and handle any reference gene expression variability in the samples that may be present in the sub-clones. Triplet of *GAPDH-CCSER2-PCBP1* should provide a potential alternative to traditionally used reference genes for reference gene matrix in MCF-7 cells. We are optimistic that MCF-7 cell line will continue to contribute eminently towards improved and novel treatment modalities for breast cancer patients.

## Conclusions

Genetic and phenotypic heterogeneity showcased by MCF-7 cell line poses a conundrum with some unanswered questions. Performing reference gene analysis may not be feasible for every sub-clone and hence, based on our results and extensive validation, we suggest using *GAPDH-CCSER2-PCBP1* triplet for normalization of genes of interest in MCF-7 cell line whilst keeping in mind the limitations of the present study. Furthermore, we suggest avoiding the use of *ACTB, GAPDH* and *PGK1* as single internal controls.

## Methods

### Culture and seeding conditions of MCF-7 biological replicate cultures (sub-clones A1 and A2)

Samples were collected from MCF-7 cell line (ATCC, HTB-22) that has been used in our laboratory for previous studies [56]. Samples from different passages were cryopreserved during culturing at different time points. Frozen stocks were taken from MCF-7 cells grown in culturing conditions as described below. For this study, two aliquots from samples that were previously cryopreserved were thawed at the same time. Culture A1 was cryopreserved after passage 27 (p27) and culture A2 was cryopreserved after passage 24 (p24). They were then cultured in the same conditions simultaneously over multiple successive passages. From each culture, three samples were collected from each passage. Culture A1 was cultured from passage 28 (p28) till passage 32 (p32) while culture A2 was cultured from passage 25 (p25) till passage 30 (p30). Cells were cultured in Dulbecco Modified Eagle’s medium with Ham’s F12 nutrient supplement DMEM/F12 (1:1) with 10% FBS (fetal bovine serum), supplemented with 1% penicillin/streptomycin (Thermo Fisher Scientific) in 37 °C, 5% CO_2_ and the growth medium was replaced every 2–3 days. Cell passaging was performed using 1x TrypLE solution (Thermo Fisher Scientific). Cells were grown to 80–100% confluence in T-25 cm^2^ flasks. Cell count and viability was estimated using a cell counting chamber (Improved Neubauer Hemocytometer). For further consecutive passages, cells were seeded at a density of 5000 cells/cm^2^. Triplicates (3 lysates/samples) of 1 × 10^6^ cells each from each passage from both cultures were taken for isolation of total RNA.

### Culturing conditions of MCF-7 cultures in nutrient stress

The MCF-7 samples from previous study in our laboratory [56] were used to validate the reference gene triplet in cells undergoing different nutrient conditions, i.e. cultured in different media. Two lysates from our cryobank were used for RNA extraction and further analysis. Culture **B5** was cultured in Medium 199 (M199) with 5% FBS; culture **D5** in DMEM/F12 (1:3) with 5% FBS; culture **E5** in DMEM/F12 (1:1) with 5% FBS and culture **R5** in Roswell Park Memorial Institute (RPMI) 1640 with 5% FBS. A detailed description of growth media composition is presented in Additional file 6. Similar to cultures A1 and A2, these cultures were also grown in 37 °C, 5% CO_2_ with growth media replaced every 2–3 days. Identical protocol was followed for cell count and viability as described in previous section.

### RNA extraction and cDNA synthesis

Total RNA was extracted using Trizol reagent (Thermo Fisher Scientific) according to the manufacturer’s protocol. The concentration and quality of the RNA was assessed by Nanodrop 2000 with the mean absorption ratios A260/280 and A260/230 checked to ensure RNA purity. RNA integrity was checked using 1.8% agarose gel electrophoresis. The RNA was further examined for DNA contamination by PCR for *ACTB* and *GAPDH*. The PCR reaction was performed in the presence of both positive and negative controls. No amplified PCR product was found on the agarose gel after PCR and electrophoresis of the RNA samples (except for positive controls). The cDNA synthesis reaction was carried out using the High Capacity cDNA Reverse transcription kit (Thermo Fisher Scientific), in accordance with the manufacturer’s protocol and guidelines and was stored at − 20 °C until further analysis.

### Selection of candidate reference gene and primer design

In total, 12 candidate housekeeping genes were selected by perusing relevant literature related to breast cancer and/or MCF-7 cell line and literature that reported transcriptomic data based on TCGA (The Cancer Genome Atlas) database [25–28, 33–37]. All selected genes are shown in Additional file 4. The selected genes have been time and again reported as suitable internal controls or have been described with controversial findings. The studies span over almost two decades from 2000 to 2019 and have never been collectively investigated in MCF-7 cell line. Hence, the present study took these findings from previous studies into account while selecting the genes.

The sources of gene primers and primer sequence used for the reference genes are shown in Additional file 4. The present study employed the primers that were reported before in the literature to maintain inter-reliability and inter-connectivity with the previous studies. Primers for *RNA28S* and *PGK1* were designed using Primer3Plus [57]. The melting curves of all selected gene primers are presented in Additional file 4. The primer sequences (designed using Primer3Plus) for genes of interest, *AURKA* and *KRT19* are also presented in Additional file 4.

### Real time quantitative PCR (RT-qPCR)

Real time quantitative PCR (qPCR) was performed using 10 ng of cDNA per reaction on ViiA 7 RT-PCR thermocycler (Thermo Fisher Scientific). Triplicate reactions of each sample were performed using HOT FIREPol EvaGreen qPCR Supermix (Solis Biodyne) on 384 well plates (Thermo Fisher Scientific). The cycling parameters were: 95 °C for 4 mins followed by 40 cycles of amplification at 95 °C for 30 s and 58 °C for 20 s followed by 72 °C for 30 s with melting curve. All assays were performed with a non-template control (NTC).

### Algorithms and statistical analysis

A detailed description of criteria and methodology with suitable illustrations and examples is shown in Additional file 5. Statistical significance was set at *P* < 0.05 (unless otherwise stated) and was calculated using Analysis of Variance (one-way ordinary *ANOVA* (with relevant multiple comparisons test and corrections) where data was distributed normally. For non-normally distributed data (checked using Shapiro-Wilk test), the non-parametric *ANOVA* (*Kruskal-Wallis ANOVA*) with respective corrections and multiple comparisons was used. The comparisons were made to compare the gene expression at successive passages to the gene expression at the smallest passage number. Data Management and storage along with descriptive statistics were done using MS Excel (Microsoft Office 365). The statistical analysis was done using *JASP* (Jeffery’s Amazing Statistical Program) v.0.10.2.0 and *R Studio* v3.6.3.

### TCGA Transcriptomic bioinformatics

The R based package < TCGAbiolinks > [58–60] was used to retrieve the TCGA gene expression data. The TCGA legacy archive was accessed for open access patient data. The legacy archive has unmodified copy of data that was previously stored in CGhub and TCGA data portal and uses GRCh37 (hg 19) as references. After downloading and preparing the data (GDCdownload and GDCprepare function), the data was exported to Excel for further analysis.

## Supplementary information

**Additional file 1:** Supplementary Tables.

**Additional file 2:** Supplementary Figures.

**Additional file 3:** Extended Discussion.

**Additional file 4:** Primer Design & Sequence.

**Additional file 5:** Calculations & Algorithms.

**Additional file 6:** Control & Stress Media.

## Data Availability

The datasets generated for Genes of Interest GOI 1 and 2 are available as Supplementary Tables S6 and S7 respectively (see Additional file 1). The primer sequences for all genes studied in the study are available in Additional file 4. Suitable illustrations and examples of calculations performed in the present study are shown in Additional file 5. List of reagents used, and their manufacturers are enlisted in Additional file 6. TCGA dataset is available for download from TCGAbiolinks package (R studio) or TCGA repository. Data is available from the corresponding author upon reasonable request via email provided (inese.cakstina@rsu.lv).

## Abbreviations

MCF-7: Michigan Cancer Foundation – 7
RGs: Reference genes
RT-PCR: Real time – polymerase chain reaction
p: Passage
GOI: Gene of Interest
CV%: Coefficient of Variation %
S.D.: Standard Deviation
TPM: Transcripts per Million
BRCA: Breast Invasive Carcinoma
TCGA: The Cancer Genome Atlas
HUGO: Human Genome Organisation
Lum A: Luminal A Molecular Subtype Breast Cancer
FBS: Fetal Bovine Serum
*ACTB*: Actin Beta
*GAPDH*: Glyceraldehyde-3-phosphate Dehydrogenase
*RPL13A*: Ribosomal Protein L13a
*PGK1*: Phosphoglycerate Kinase 1
*HSPCB*: Heat Shock Protein 90 Alpha family Class B member 1
*RNA18S*: RNA, 18S ribosomal
*RNA28S*: RNA, 28S ribosomal
*PUM1*: Pumilio RNA Binding family member
*CCSER2*: Coiled-Coil Serine Rich protein 2
*HNRNPL*: Heterogenous Nuclear Ribonucleoprotien L
*PCBP1*: Poly(rC) Binding protein 1
*SF3A1*: Splicing factor 3a subunit 1
*AURKA*: Aurora Kinase A
*KRT19*: Keratin 19
