# Supplementary material for: Selecting suitable reference genes for qPCR normalization: a comprehensive analysis in MCF-7 breast cancer cell line
Source: BMC Mol Cell Biol. 2020 Sep 25;21:68. doi: 10.1186/s12860-020-00313-x (PMC7519550; doi:10.1186/s12860-020-00313-x)
Supplement: Supplementary file 1 — Additional file 1: Supplementary Tables. [file 12860_2020_313_MOESM1_ESM.pdf]

ADDITIONAL FILE 1: SUPPLEMENTARY TABLES

Selecting Suitable Reference Genes for qPCR Normalization: A Comprehensive Analysis in MCF-7 Breast Cancer Cell Line

Authors: Nityanand Jain, Dina Nitisa, Valdis Pirsko and Inese Cakstina*

*** For Correspondence:**

Laboratory of Molecular Genetics
Institute of Oncology
Riga Stradins University
16 Dzirciema street
Riga
Latvia (LV-1007)

Email: inese.cakstina@rsu.lv

INDEX

1) Supplementary Table S1 – Relative Mean Changes in Expression Profiles (*Section 2.3*)
2) Supplementary Table S2 – NormFinder Analysis (*Section 2.4*)
3) Supplementary Table S3 – BestKeeper Standard Deviation with change in x-folds (*Section 2.6*)
4) Supplementary Table S4 – Comparative ΔCt Analysis (*Section 2.7*)
5) Supplementary Table S5 – RefFinder Analysis (*Section 2.8*)
6) Supplementary Table S6 – Gene of Interest 1 (*GOI 1*) Simulated dataset (*Section 2.10*)
7) Supplementary Table S7 – Gene of Interest 2 (*GOI 2*) Simulated dataset (*Section 2.10*)
8) Supplementary Table S8 – Normalization of *GOI 1* using 3 reference genes (*Section 2.10*)
9) Supplementary Table S9 – Normalization of *GOI 2* using 3 reference genes (*Section 2.10*)
10) Supplementary Table S10 – Normalization of *AURKA* using 3 reference genes (*Section 2.11*)
11) Supplementary Table S11 – Normalization of *KRT19* using 3 reference genes (*Section 2.11*)
12) Supplementary Table S12 – TCGA – normalized count for reference genes (*Section 2.13*)
13) Supplementary Table S13 – TCGA – transcripts per million (TPM) for reference genes (*Section 2.13*)
14) Supplementary Table S14 – Expression fold change in control vs stress cultures (*Section 2.15*)

Supplementary Table S1. *ANOVA P* values showing comparisons between mean expression changes in both the cultures over successive passages

Candidate Gene	Replicate Culture A1	Replicate Culture A2
ACTB	$P < 0.001$***	$P < 0.001$***
GAPDH	$P = 0.003$**	$P < 0.001$***
RPL13A	$P < 0.001$***	$P < 0.001$***
PGK1	$P < 0.001$***	$P < 0.001$***
HSPCB	$P < 0.001$***	$P < 0.001$***
RNA28S	$P = 0.038$*	$P < 0.0001$***
RNA18S	$P < 0.001$***	$P < 0.001$***
PUM1	$P = 0.004$**	$P = 0.011$*
CCSER2	$P = 0.224$	$P = 0.015$*
HNRNPL	$P < 0.001$***	$P < 0.001$***
PCBP1	$P = 0.710$	$P < 0.001$***
SF3A1	$P = 0.008$**	$P < 0.001$***

* P value is significant at $P < 0.05$. ** P value is significant at $P < 0.01$. *** P value is significant at $P < 0.001$.

Supplementary Table S2. Most stable pair of genes with their standard deviations for both replicate cultures as determined by NormFinder

Replicate Culture A1			Replicate Culture A2		
**Gene Pair**	**Standard Deviation (S.D)**	**Rank**	**Gene Pair**	**Standard Deviation (S.D)**	**Rank**
ACTB + PUM1	0.07	1	*RPL13A + SF3A1*	0.07	1
GAPDH + PCBP1	0.08	2	*GAPDH + SF3A1*	0.07	1
PGK1 + PUM1	0.08	2	*RPL13A + HSPCB*	0.08	2
PGK1 + CCSER2	0.08	2	*ACTB + SF3A1*	0.08	2
PGK1 + PCBP1	0.09	3	*HSPCB + PCBP1*	0.09	3
GAPDH + PUM1	0.09	3	*HSPCB + SF3A1*	0.09	3
GAPDH + CCSER2	0.10	4	*RPL13A + PCBP1*	0.09	3
PUM1 + PCBP1	0.21	5	*RPL13A + CCSER2*	0.09	3
ACTB + PCBP1	1.83	6	*GAPDH + CCSER2*	0.09	3
CCSER2 + PCBP1	2.46	7	*GAPDH + PCBP1*	0.10	4

Supplementary Table S3. BestKeeper ranking of reference genes based on standard deviation (S.D) with changes in x-folds for both replicate cultures

Gene	Rank		S.D ± x-fold		Minimum x-fold		Maximum x-fold	
	**A1**	**A2**	**A1**	**A2**	**A1**	**A2**	**A1**	**A2**
ACTB	4	1	1.15	1.22	-1.52	-1.50	1.37	1.42
GAPDH	1	4	1.11	1.27	-1.27	-1.56	1.31	1.53
RPL13A	5	5	1.18	1.28	-1.25	-1.52	1.52	1.94
PGK1	3	9	1.14	1.47	-1.23	-1.73	1.60	2.04
HSPCB	6	7	1.19	1.33	-1.30	-1.65	1.49	2.55
RNA28S	3	2	1.14	1.23	-1.59	-1.44	1.28	2.34
RNA18S	5	6	1.18	1.29	-1.40	-1.59	1.46	2.99
PUM1	3	2	1.14	1.23	-1.33	-1.66	1.45	2.21
CCSER2	2	4	1.13	1.27	-1.46	-1.45	1.29	1.69
HNRNPL	8	8	1.23	1.42	-1.53	-1.91	1.72	2.12
PCBP1	2	3	1.13	1.26	-1.28	-1.43	1.55	1.71
SF3A1	7	4	1.20	1.27	-1.32	-1.66	1.84	2.25

Supplementary Table S4. Comparative ΔCt results with average Standard deviation (S.D) of genes in both cultures

Culture A1			Culture A2		
**Candidate Gene**	**Average S.D**	**Rank**	**Candidate Gene**	**Average S.D**	**Rank**
GAPDH	0.29	1	*RNA28S*	0.28	1
CCSER2	0.30	2	*GAPDH*	0.28	1
PCBP1	0.30	2	*PCBP1*	0.28	1
PGK1	0.31	3	*RPL13A*	0.30	2
ACTB	0.32	4	*HSPCB*	0.31	3
PUM1	0.33	5	*HNRNPL*	0.32	4
HSCPB	0.36	6	*ACTB*	0.32	4
RNA18S	0.37	7	*RNA18S*	0.32	4
RNA28S	0.37	7	*SF3A1*	0.32	4
RPL13A	0.37	7	*CCSER2*	0.33	5
HNRNPL	0.42	8	*PGK1*	0.38	6
SF3A1	0.43	9	*PUM1*	0.39	7

Supplementary Table S5. Ranking of the candidate genes by RefFinder for both cultures A1 and A2

Culture A1			Culture A2		
**Candidate Gene**	**Geomean**	**Rank**	**Candidate Gene**	**Geomean**	**Rank**
GAPDH	1.41	1	*RNA28S*	1.41	1
CCSER2	2.78	2	*GAPDH*	2.89	2
PCBP1	3.22	3	*PCBP1*	3.83	3
ACTB	3.64	4	*ACTB*	4.14	4
PGK1	3.72	5	*RPL13A*	4.43	5
HSPCB	4.86	6	*RNA18S*	4.90	6
PUM1	6.24	7	*HSPCB*	5.62	7
RNA28S	7.49	8	*CCSER2*	7.75	8
RNA18S	8.24	9	*HNRNPL*	8.24	9
RPL13A	9.49	10	*SF3A1*	8.35	10
HNRNPL	11.24	11	*PUM1*	8.49	11
SF3A1	11.74	12	*PGK1*	11.24	12

CRITERIA FOR ASSIGNMENT OF Cq VALUES FOR *GOI 1*

While assigning the Cq values for *GOI 1 (simulated to have stable gene expression)*, it was considered that the difference between Cq values doesn't exceed +/- 0.5 Cq. To randomize the Cq values and minimize human intervention, the values were generated using an online random fraction generator tool (https://onlinerandomtool.com/generate-random-fractions). The *GOI 1* was then normalized using ΔΔCt method to the selected reference genes for both cultures. Significance of fold change was estimated using $P < 0.05$ (indicating significant change) from one-way *ANOVA* and respective post-hoc tests (with Bonferroni *P* value correction).

Supplementary Table S6. Simulated dataset containing Cq values created for Gene of Interest *GOI 1.* The Cq values were created in triplicates for 3 lysates over 4 passages (culture A1) and over 5 passages (culture A2) to resonate with the dataset like the one for the reference genes used in this study

Passage	Lysate 1	Lysate 2	Lysate 3
Culture A1			
p28	9.85	9.65	*
p28	9.35	9.82	*
p28	9.41	9.39	*
p30	10.11	9.99	9.87
p30	9.85	9.85	10.23
p30	9.69	9.46	10.01
p31	10.02	9.57	*
p31	9.99	9.68	*
p31	9.35	9.89	*
p32	10.19	9.76	10.25
p32	9.85	9.65	10.21
p32	9.88	9.99	9.49
Culture A2			
p25	9.36	9.89	10.33
p25	9.45	10.25	9.64
p25	9.53	9.68	9.78
p26	10.10	9.45	10.23
p26	9.63	9.35	10.18
p26	9.86	9.76	10.06

p28	9.89	9.46	9.68
p28	9.65	10.26	9.55
p28	9.36	10.10	9.69
p29	9.68	9.99	10.32
p29	9.56	10.25	9.58
p29	9.78	10.14	9.46
p30	9.35	9.66	10.30
p30	9.65	9.88	9.50
p30	9.79	10.21	9.42
Median (and Range) of Cq values		9.85 (9.35 - 10.35)	
Mean ± S.D for Culture A1		9.81 ± 0.27	
Mean ± S.D for Culture A2		9.79 ± 0.31	

*Lysate 3 for both p28 and p31 were removed due to DNA presence in RNA in the original dataset of reference genes and hence to resonate this dataset with our original qPCR results, here as well the lysates were removed.

CRITERIA FOR ASSIGNMENT OF Cq VALUES FOR *GOI 2*

While assigning the Cq values for *GOI 2 (simulated to have unstable gene expression)*, it was considered that the difference between Cq values should exceed +/- 0.5 Cq. To randomize the Cq values and minimize human intervention, the values were generated using an online random fraction generator tool (https://onlinerandomtool.com/generate-random-fractions). The *GOI 2* was then normalized using ΔΔCt method to the selected reference genes for both cultures. Significance of fold change was estimated using $P < 0.05$ (indicating significant change) from one-way *ANOVA* and respective post-hoc tests (with Bonferroni *P* value correction).

Supplementary Table S7. Simulated dataset containing Cq values created for Gene of Interest *GOI 2.* The Cq values were created in triplicates for 3 lysates over 4 passages (culture A1) and over 5 passages (culture A2) to resonate with the dataset like the one for the reference genes used in this study

Passage	Lysate 1	Lysate 2	Lysate 3
Culture A1			
p28	20.10	20.69	*
p28	21.45	21.43	*
p28	20.60	20.36	*
p30	21.25	20.98	22.01
p30	22.12	21.56	21.87
p30	20.43	20.66	21.05
p31	21.12	22.05	*
p31	20.96	21.75	*
p31	20.36	21.65	*
p32	22.85	20.68	22.18
p32	22.65	20.96	21.63
p32	21.55	21.16	21.89
Culture A2			
p25	20.05	21.72	21.12
p25	22.79	22.53	22.04
p25	22.44	22.57	20.91
p26	21.59	22.14	21.43
p26	21.80	20.85	22.81
p26	21.07	22.65	21.94

p28	20.72	21.67	22.05
p28	21.35	21.82	22.28
p28	22.85	22.56	20.97
p29	21.25	20.37	21.06
p29	22.46	20.33	21.44
p29	21.00	20.76	20.49
p30	21.51	21.96	21.22
p30	21.34	22.23	20.84
p30	20.66	22.59	21.99
Median (and Range) of Cq values		21.50 (20.02- 22.90)	
Mean ± S.D for Culture A1		21.33 ± 0.69	
Mean ± S.D for Culture A2		21.60 ± 0.76	

*Lysate 3 for both p28 and p31 were removed due to DNA presence in RNA in the original dataset of reference genes and hence to resonate this dataset with our original qPCR results, here as well the lysates were removed.

Supplementary Table S8. Changes in gene expression levels expressed as fold change of *GOI 1* after normalization with pair of three reference genes calculated using ΔΔCt method for culture A1 and A2

Passage	*GAPDH/PCBP1/ CCSER2*	*GAPDH/PCBP1/ RNA28S*	*GAPDH/CCSER2/ RNA28S*	*PCBP1/CCSER2/ RNA28S*
**Culture A1**				
**p30**	1.1736	1.3004	1.2049	1.3005
**p31**	1.0588	1.0852	1.0713	1.3301
**p32**	1.2801	1.3058	1.2924	1.2979
**Culture A2**				
**p26**	0.9708	1.0606	1.0223	0.9752
**p28**	0.5701	0.5593*	0.5604*	0.5673*
**p29**	0.7941	0.7927	0.8376	0.7660
**p30**	0.7541	0.7441	0.7627	0.8025

* Significant fold expression change at $P < 0.05$ (ANOVA with Post Hoc and Bonferroni P value correction) in comparison with p28 for Culture A1 and p25 for Culture A2 when *GOI 1* is normalized with the specific pair of 3 reference genes.

Supplementary Table S9. Changes in gene expression levels expressed as fold change of *GOI 2* after normalization with pair of 3 reference genes calculated using ΔΔCt method for culture A1 and A2

**Passage**	*GAPDH/PCBP1/ CCSER2*	*GAPDH/PCBP1/ RNA28S*	*GAPDH/CCSER2/ RNA28S*	*PCBP1/CCSER2/ RNA28S*
**Culture A1**				
**p30**	1.3828	1.5323	1.4197	1.5318
**p31**	1.3699	1.4041	1.3861	1.4659
**p32**	1.9613	2.0079*	1.9802*	1.9886*
**Culture A2**				
**p26**	0.9312	1.0127	0.9762	0.9312
**p28**	0.5866	0.5755	0.5766	0.5806
**p29**	0.4335*	0.4327*	0.4572*	0.4352*
**p30**	0.6626	0.6538	0.6701	0.6726

* Significant fold expression change at $P < 0.05$ (ANOVA with Post Hoc and Bonferroni P value correction) in comparison with p28 for Culture A1 and p25 for Culture A2 when *GOI 2* is normalized with the specific pair of 3 reference genes.

Supplementary Table S10. Changes in gene expression levels expressed as fold change of *AURKA* after normalization with pair of three reference genes calculated using ΔΔCt method for culture A1 and A2

**Passage**	***GAPDH/PCBP1/ CCSER2***	***GAPDH/PCBP1/ RNA28S***	***GAPDH/CCSER2/ RNA28S***	***PCBP1/CCSER2/ RNA28S***
**Culture A1**				
**p30**	1.8341	2.0345*	1.8834	2.0317*
**p31**	1.1912	1.2209	1.2053	1.2747
**p32**	1.1792	1.2029	1.1906	1.1956
**Culture A2**				
**p26**	1.1744	1.2774*	1.2313*	1.1745
**p28**	0.9803	0.9619	0.9637	0.9756
**p29**	0.9208	0.9192	0.9713	0.8882
**p30**	1.0090	0.9955	1.0205	1.0737

* Significant fold expression change at $P < 0.05$ (ANOVA with Post Hoc and Bonferroni P value correction) in comparison with p28 for Culture A1 and p25 for Culture A2 when *AURKA* is normalized with the specific pair of 3 reference genes.

Supplementary Table S11. Changes in gene expression levels expressed as fold change of *KRT19* after normalization with pair of three reference genes calculated using ΔΔCt method for culture A1 and A2

Passage	*GAPDH/PCBP1/ CCSER2*	*GAPDH/PCBP1/ RNA28S*	*GAPDH/CCSER2/ RNA28S*	*PCBP1/CCSER2/ RNA28S*
**Culture A1**				
**p30**	1.3446	1.4899*	1.3804	1.4894
**p31**	1.2947	1.3270*	1.3100*	1.3854*
**p32**	1.0207	1.0412	1.0305	1.0349
**Culture A2**				
**p26**	1.6928	1.8414	1.7749	1.6931
**p28**	2.4361	2.4829*	2.4408*	2.4709
**p29**	2.1627	2.1590	2.2813*	2.0863
**p30**	2.7040	2.6678	2.7347	2.8774*

* Significant fold expression change at $P < 0.05$ (ANOVA with Post Hoc and Bonferroni P value correction) in comparison with p28 for Culture A1 and p25 for Culture A2 when *KRT19* is normalized with the specific pair of 3 reference genes.

TCGA DATASET RETRIEVAL DESCRIPTION (via R)

R (via R Studio) was used to retreive the data with the help of *TCGAbiolinks* package (available on Bioconductor). Clinical, Morphological and Expression data of 1215 patients (open access) was retrieved under the project "TCGA-BRCA"(Platform - Illumina HiSeq; file.type - rsem.genes.normlised_results). The "*normalised_count*" is a simple transformation of the "*raw_count*" (available in the file with extension rsem.genes.results). The "*raw_count*" values are divided by the 75th percentile and then multiplied by 1000 to obtain "*normalised_count*" to make the values comparable between experiments.

Supplementary Table S12. Descriptive analysis of the normalized expression count as obtained from TCGA database (Lum A BRCA)

Candidate Reference Gene	Minimum (RSEM)	Median (RSEM)	Maximum (RSEM)	Difference (Max-Min)
GAPDH	12851.20	41483.10	458078.40	445227.20
RPL13A	3437.64	14,620.59	96084.24	92646.60
PGK1	1770.74	7272.16	50,683.05	48912.31
HSPCB**	13752.21	31398.41	119980.80	106228.59
PUM1	642.36	2374.92	4575.92	3933.56
CCSER2	204.81	1141.41	4501.78	4296.97
HNRNPL	2995.01	5132.91	11451.14	8456.13
PCBP1	3732.21	7157.66	13467.68	9735.47
SF3A1	856.04	2854.47	5524.61	4668.57

** *HSPCB* was retrieved as *HSP90AB1* (HUGO gene nomenclature committee, 2020) from the database. RSEM refers to RNA-Seq by Expectation Maximization and is used by the TCGA RNASeqV2 pipeline for normalization of raw counts.

TCGA DATASET RETRIEVAL DESCRIPTION (via R)

R (via R Studio) was used to retreive the data with the help of *TCGAbiolinks* package (available on Bioconductor). Clinical, Morphological and Expression data of 1215 patients (open access) was retrieved under the project "TCGA-BRCA" (Platform - Illumina HiSeq; file.type - rsem.genes.results). The *"scaled_estimate"* (estimated frequency of gene/transcripts among the total number of transcripts that were sequenced) values were obtained. TPM is obtained by multiplying *"scaled_estimate"* values by a factor of 1 million (10^6).

Supplementary Table S13. Mean and CV% of the log2TPM (transcripts per million) data as obtained from TCGA database

Candidate Reference Gene	Mean (log2 TPM)	S.D. (log2 TPM)	CV% (S.D/Mean)
ACTB	12.15	0.52	4.30%
GAPDH	11.78	0.76	6.44%
RPL13A	10.32	0.64	6.18%
PGK1	8.14	0.73	8.93%
HSPCB**	10.18	0.58	5.66%
PUM1	5.27	0.57	10.90%
CCSER2**	3.97	0.69	17.49%
HNRNPL	7.92	0.33	4.22%
PCBP1	8.52	0.33	3.96%
SF3A1	5.48	0.55	10.08%

** *HSPCB* was retrieved as *HSP90AB1* while *CCSER2* was retrieved as *FAM190B* (HUGO gene nomenclature committee, 2020) from the database.

Supplementary Table S14. Fold change in reference gene expression in control (A1 and A2) versus nutrient stress (B5, D5, E5 and R5) cultures

	Culture A1 (p28)	Culture A2 (p25)	MCF-7 (A1 + A2)
ACTB			
B5	2.286	2.018	2.126
D5	2.213	1.953	2.057
E5	3.281	2.896	3.050
R5	2.396	2.115	2.228
GAPDH			
B5	1.077	1.285	1.202
D5	0.865	1.032	0.965
E5	2.129	2.541	2.376
R5	0.967	1.154	1.080
RPL13A			
B5	0.459	0.702	0.605
D5	0.458	0.700	0.603
E5	0.548	0.838	0.722
R5	0.762	1.166	1.005
PGK1			
B5	1.947	2.772	2.442
D5	1.773	2.525	2.224
E5	1.748	2.489	2.193
R5	1.575	2.243	1.976
HSPCB			
B5	0.775	0.921	0.863
D5	1.320	1.570	1.470
E5	1.555	1.849	1.731
R5	0.996	1.185	1.109
RNA28S			
B5	1.020	1.279	1.176
D5	1.098	1.377	1.266
E5	0.859	1.077	0.990
R5	1.244	1.560	1.433
RNA18S			
B5	0.653	0.763	0.719
D5	0.685	0.799	0.753
E5	0.740	0.865	0.815
R5	0.810	0.947	0.892
PUM1			

B5	1.190	1.336	1.278
D5	1.114	1.250	1.196
E5	1.688	1.895	1.812
R5	0.982	1.102	1.054
CCSER2			
B5	0.970	1.551	1.081
D5	0.785	0.935	0.875
E5	1.046	1.246	1.166
R5	0.833	0.992	0.929
HNRNPL			
B5	0.840	1.117	1.006
D5	1.101	1.463	1.318
E5	1.677	2.230	2.009
R5	1.162	1.544	1.391
PCBP1			
B5	0.789	1.011	0.922
D5	0.728	0.933	0.851
E5	0.842	1.078	0.984
R5	0.666	0.853	0.778
SF3A1			
B5	0.921	0.924	0.923
D5	0.848	0.852	0.849
E5	1.031	1.034	1.033
R5	0.851	0.854	0.853
