# Supplementary material for: Selecting suitable reference genes for qPCR normalization: a comprehensive analysis in MCF-7 breast cancer cell line
Source: BMC Mol Cell Biol. 2020 Sep 25;21:68. doi: 10.1186/s12860-020-00313-x (PMC7519550; doi:10.1186/s12860-020-00313-x)
Supplement: Supplementary file 2 — Additional file 2: Supplementary Figures. [file 12860_2020_313_MOESM2_ESM.pdf]

ADDITIONAL FILE 2: SUPPLEMENTARY FIGURES

Selecting Suitable Reference Genes for qPCR Normalization: A Comprehensive Analysis in MCF-7 Breast Cancer Cell Line

Authors: Nityanand Jain, Dina Nitisa, Valdis Pirsko and Inese Cakstina*

*** For Correspondence:**

Laboratory of Molecular Genetics
Institute of Oncology
Riga Stradins University
16 Dzirciema street
Riga
Latvia (LV-1007)

Email: inese.cakstina@rsu.lv

INDEX

1) Supplementary Figure S1 – Normalization of *GOI 1* using pairs of 2 reference genes (*Section 2.10*)
2) Supplementary Figure S2 – Normalization of *GOI 2* using pairs of 2 reference genes (*Section 2.10*)
3) Supplementary Figure S3 – Normalization of *AURKA* using pairs of 2 reference genes (*Section 2.11*)
4) Supplementary Figure S4 – Normalization of *KRT19* using pairs of 2 reference genes (*Section 2.11*)
5) Supplementary Figure S5 – TCGA (normalized_count+1) for reference genes (*Section 2.13*)
6) Supplementary Figure S6 – TCGA (log2 TPM) for reference genes (*Section 2.13*)
7) Supplementary Figure S7 – Relationship b/w Cq (qPCR) and log2 (TPM) from TCGA (*Section 2.14*)

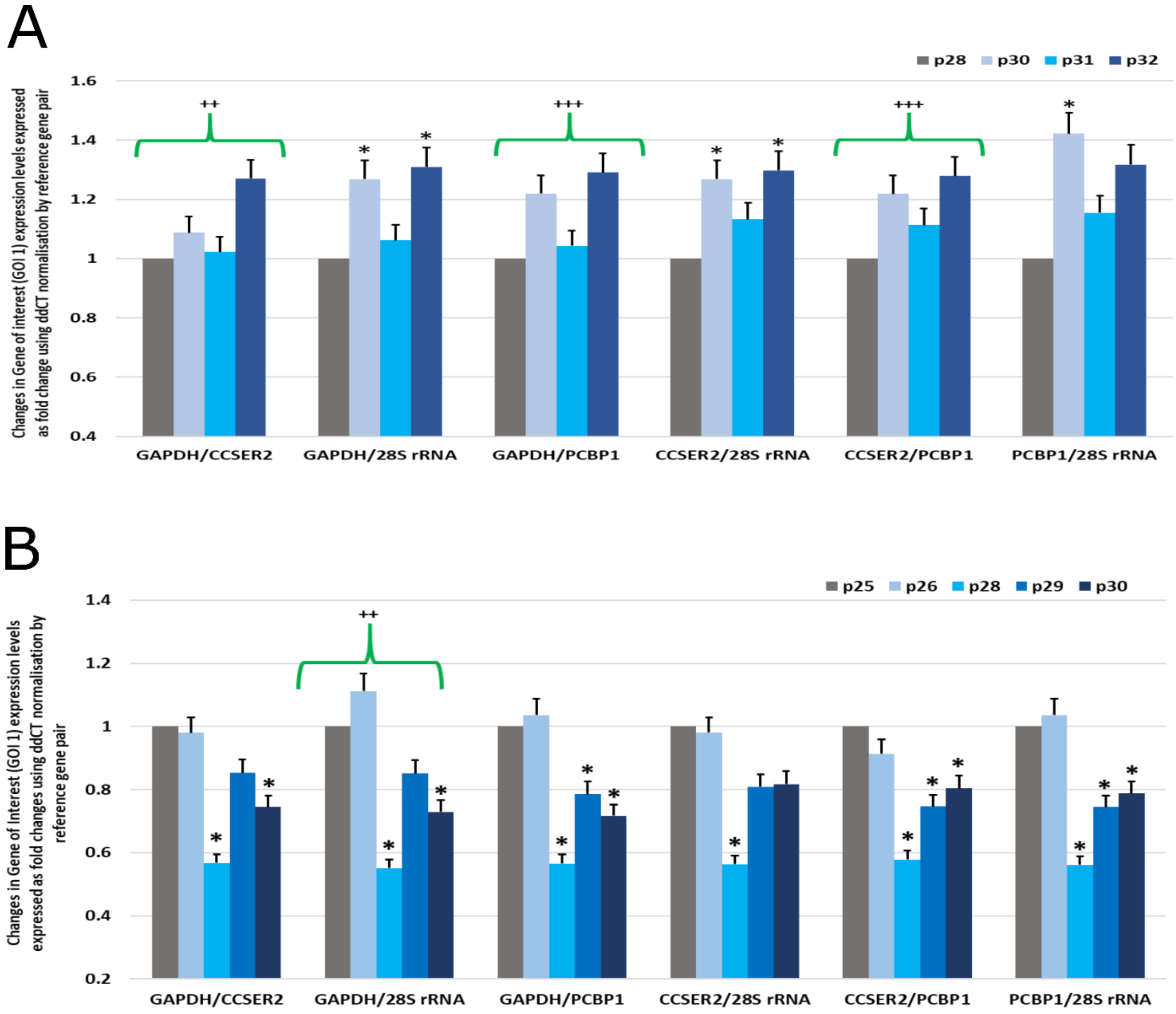

Supplementary Figure S1. Changes in Gene expression level (GOI 1) expressed as fold changes (calculated using ΔΔCt method) when normalized by reference gene pair (on x axis) and passage 28 for (**A**) culture A1 and passage 25 for (**B**) culture A2. * fold change is significant at $P < 0.05$ when compared with initial passage of respective culture (after normalization with reference gene pair). ++ gene pair considered to be the most stable as calculated by algorithms used in the study. +++ gene pairs which have no significant fold changes after normalization and are potential candidates. 28S rRNA refers to RNA28S.

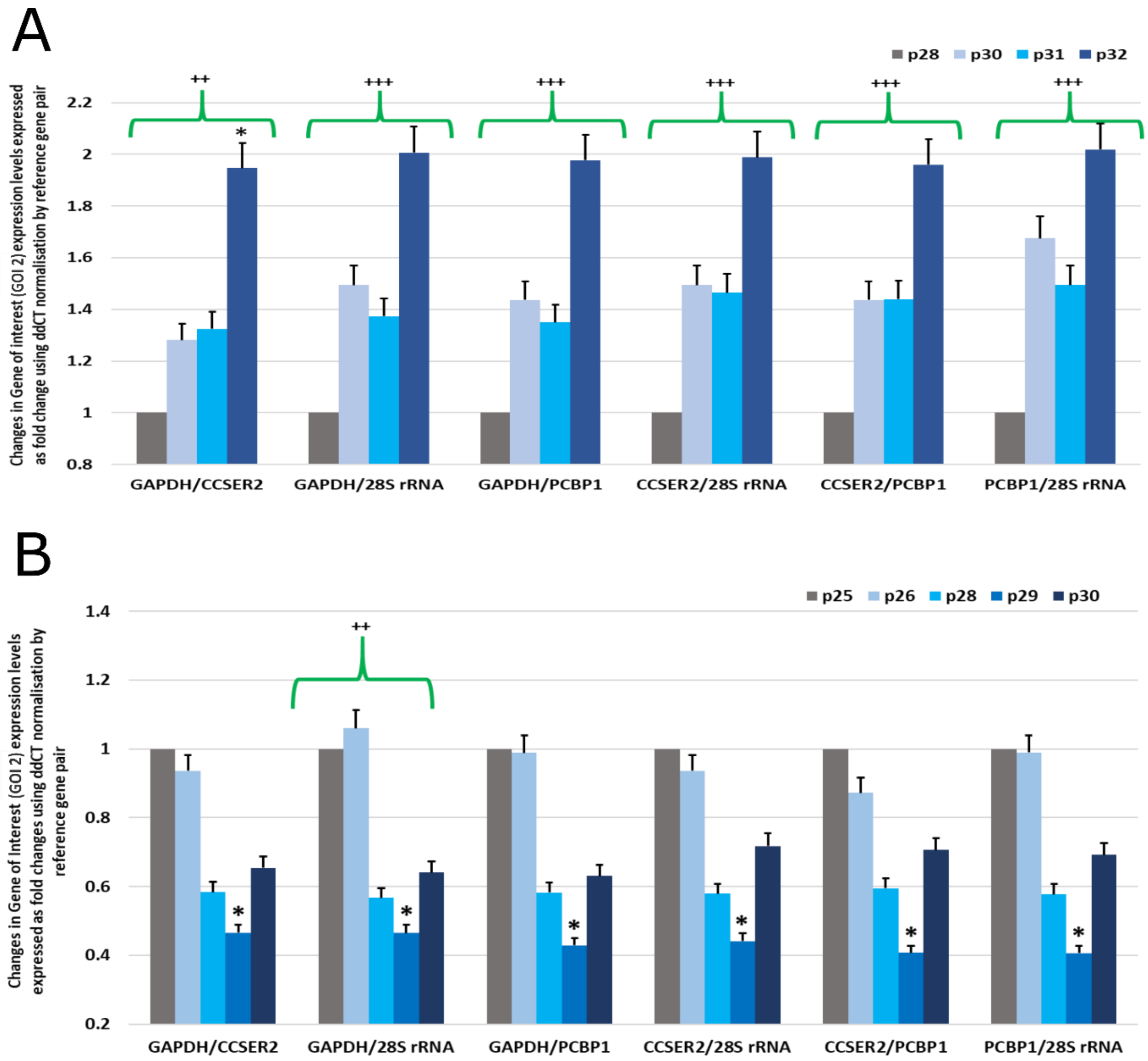

Supplementary Figure S2. Changes in Gene expression level (GOI 2) expressed as fold changes (calculated using ΔΔCt method) when normalized by reference gene pair (on x axis) and passage 28 for (**A**) culture A1 and passage 25 for (**B**) culture A2. * fold change is significant at $P < 0.05$ when compared with initial passage of respective culture (after normalization with reference gene pair). ++ gene pair considered to be the most stable as calculated by algorithms used in the study. +++ gene pairs which have no significant fold changes after normalization and are potential candidates. 28S rRNA refers to RNA28S.

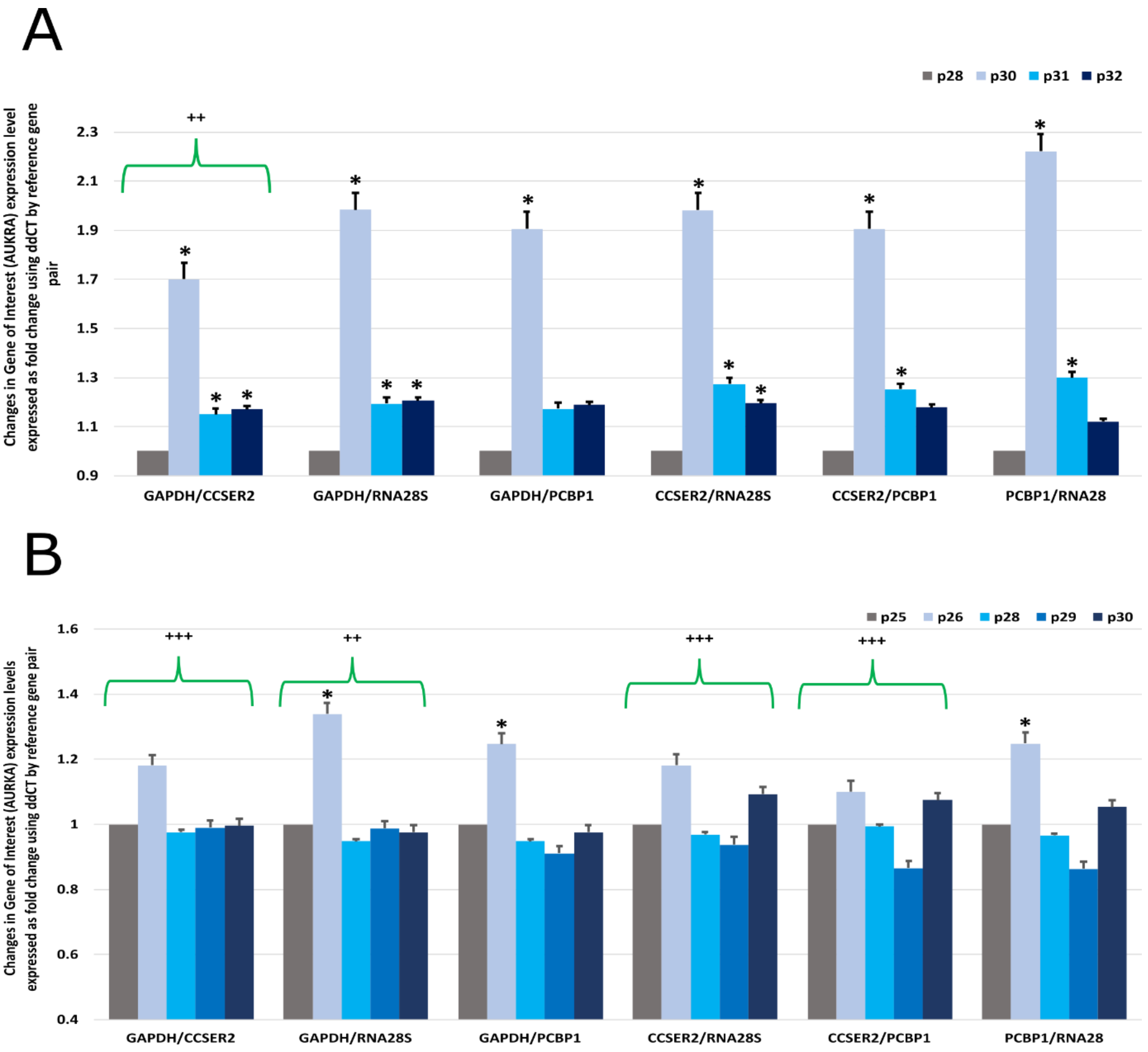

Supplementary Figure S3. Changes in Gene expression level (AURKA) expressed as fold changes (calculated using ΔΔCt method) when normalized by reference gene pair (on x axis) and passage 28 for (**A**) culture A1 and passage 25 for (**B**) culture A2. * fold change is significant at $P < 0.05$ when compared with initial passage of respective culture (after normalization with reference gene pair). ++ gene pair considered to be the most stable as calculated by algorithms used in the study. +++ gene pairs which have no significant fold changes after normalization and are potential candidates.

A

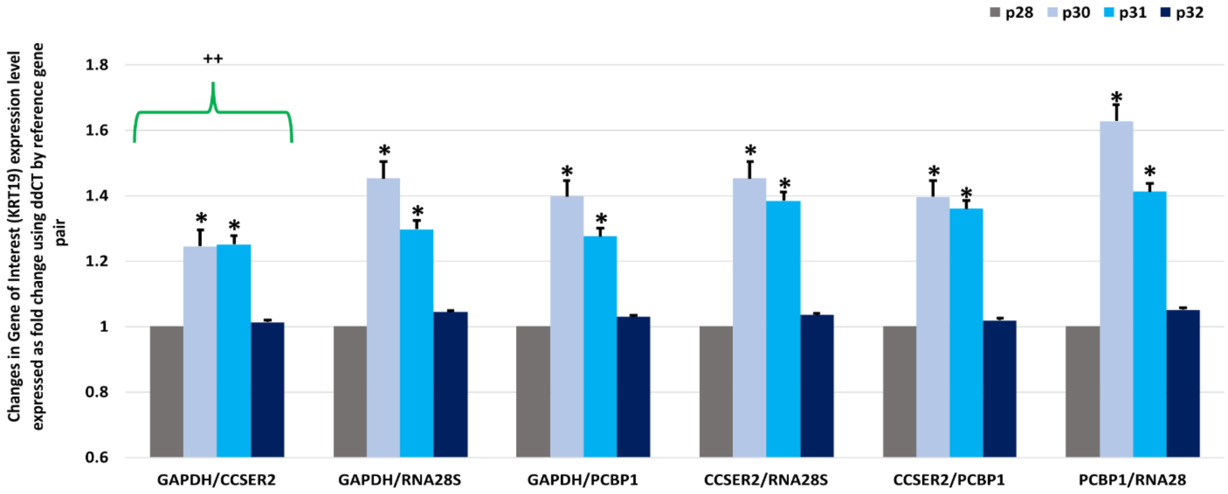

B

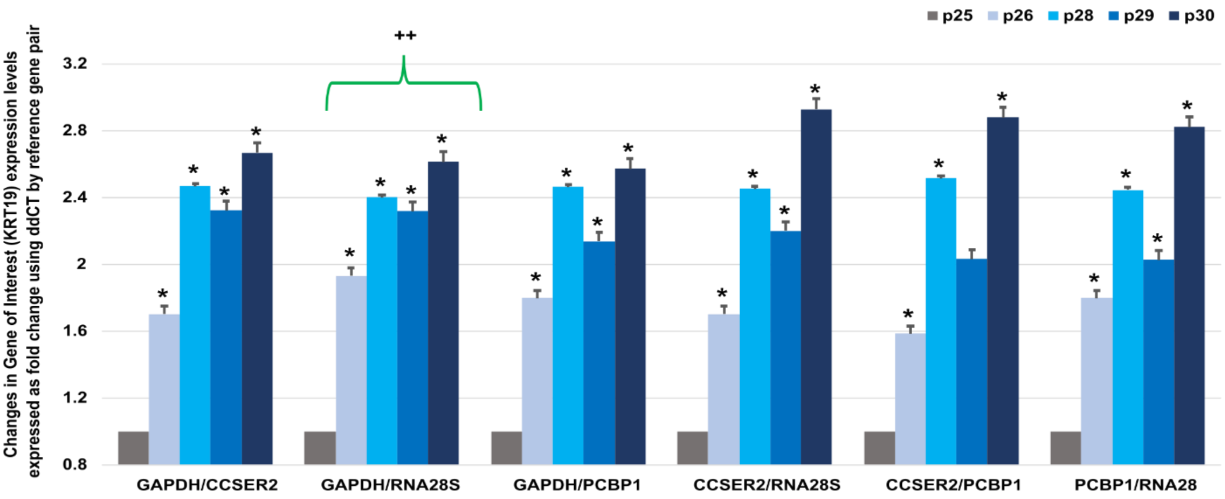

Supplementary Figure S4. Changes in Gene expression level (KRT19) expressed as fold changes (calculated using ΔΔCt method) when normalized by reference gene pair (on x axis) and passage 28 for (**A**) culture A1 and passage 25 for (**B**) culture A2. * fold change is significant at $P < 0.05$ when compared with initial passage of respective culture (after normalization with reference gene pair). ++ gene pair considered to be the most stable as calculated by algorithms used in the study. +++ gene pairs which have no significant fold changes after normalization and are potential candidates.

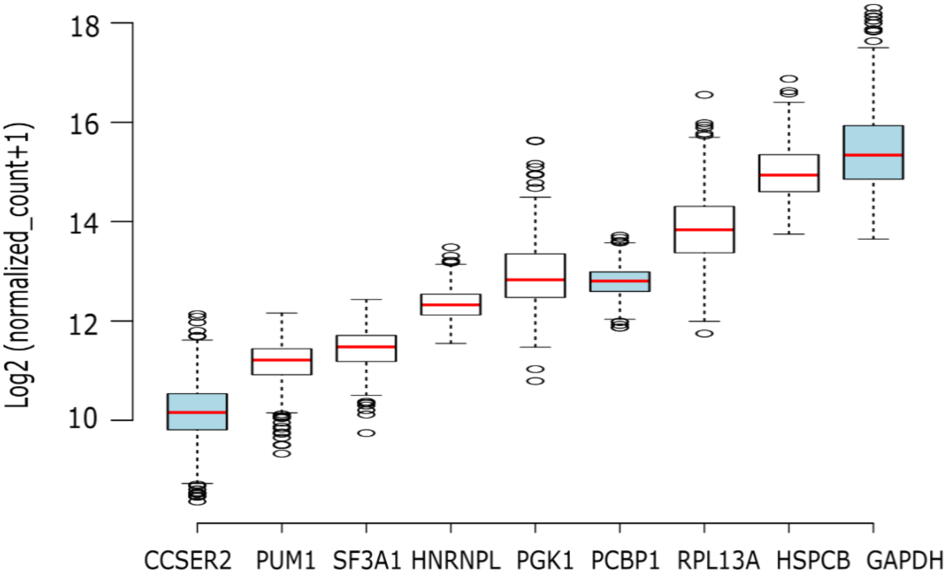

Supplementary Figure S5. Normalized Gene Expression data (RSEM) obtained from TCGA database for Luminal A subtype BRCA cancer. The data values have been converted to log scale on y axis for better visualization of data. Red lines in boxplot represents the median quartile. Blue boxplots represent the 3 reference genes identified as the most stable triplet in the present study (*CCSER2-PCBP1-GAPDH*). *HSPCB* was retrieved as *HSP90AB1* (HUGO gene nomenclature committee, 2020) from the database.

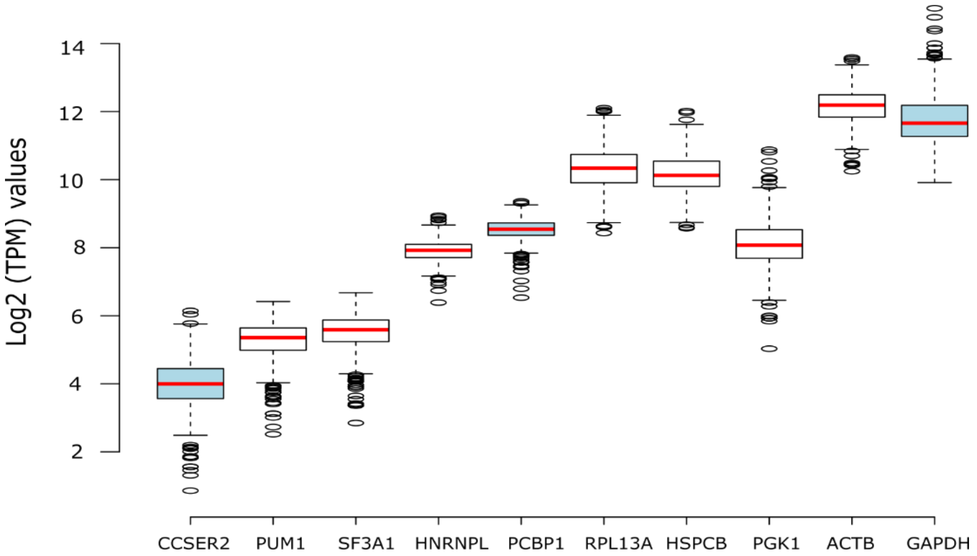

Supplementary Figure S6. TPM (transcripts per million) values obtained from TCGA database for Luminal A subtype BRCA cancer. The data values have been converted to log scale on y axis for better visualization of data. Red lines in boxplot represents the median quartile. Blue boxplots represent the 3 reference genes identified as the most stable triplet in the present study (*CCSER2-PCBP1-GAPDH*). *HSPCB* was retrieved as *HSP90AB1* while *CCSER2* was retrieved as *FAM190B* (HUGO gene nomenclature committee, 2020) from the database.

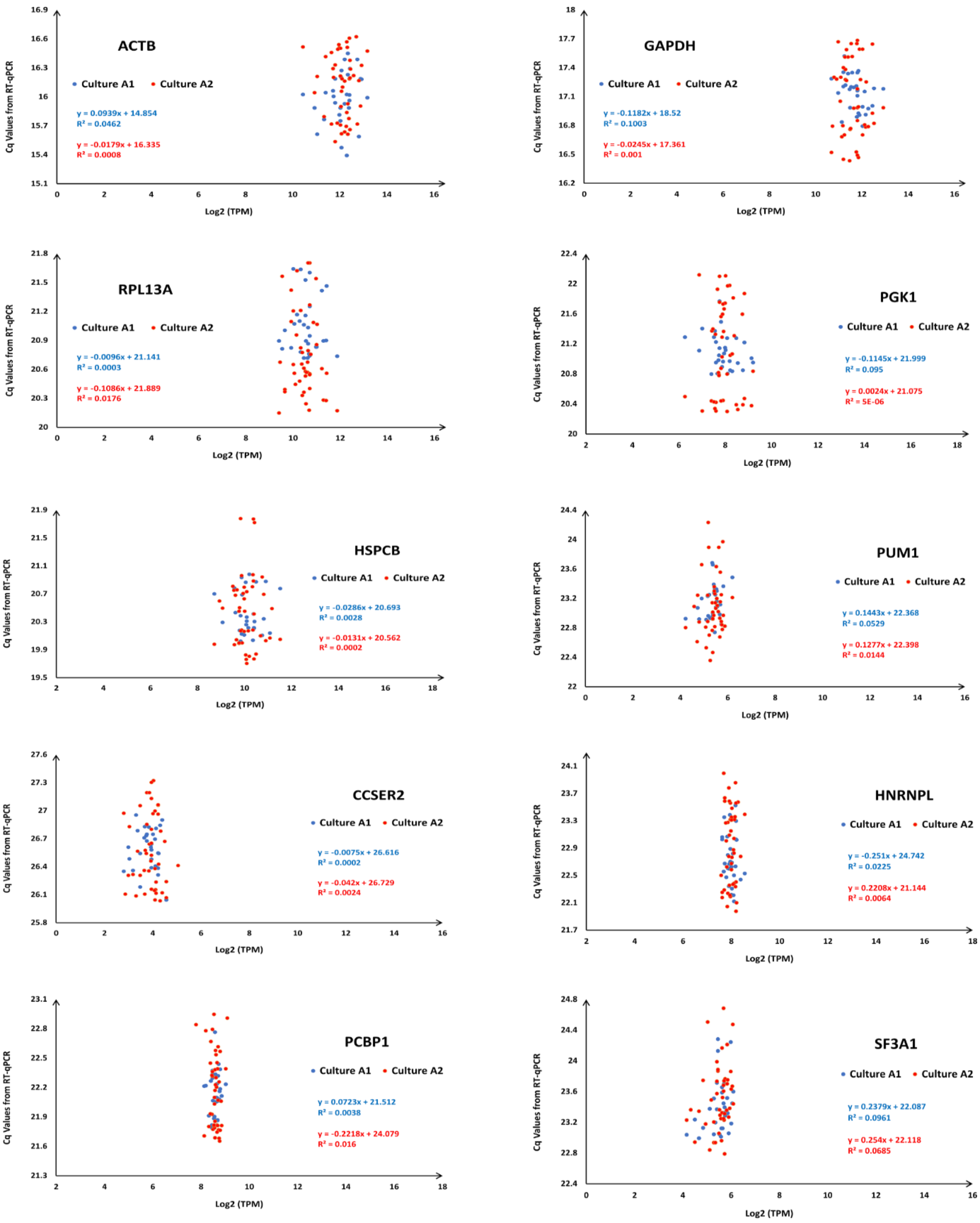

Supplementary Figure S7. Gene expression correlation shown by Log2 (TPM) vs Cq value scattered plots for all 10 reference genes. Cq values were obtained from the RT-qPCR while Log2 (TPM) values were obtained from the TCGA database. The blue dots show values from culture A1 while red dots show values from culture A2. The intercept values as well as R^2 values are shown (blue for culture A1 while red for culture A2). There is no formal relationship formula between Cq and Log2 (TPM), however, the formula presented here provides an estimation of the Ct prior to RT-qPCR experiments based on RNA-Seq data of Luminal A sub-type breast cancer.
