# Supplementary material for: Selecting suitable reference genes for qPCR normalization: a comprehensive analysis in MCF-7 breast cancer cell line
Source: BMC Mol Cell Biol. 2020 Sep 25;21:68. doi: 10.1186/s12860-020-00313-x (PMC7519550; doi:10.1186/s12860-020-00313-x)
Supplement: Supplementary file 3 — Additional file 3: Extended Discussion. [file 12860_2020_313_MOESM3_ESM.pdf]

ADDITIONAL FILE 3: EXTENDED DISCUSSION

Selecting Suitable Reference Genes for qPCR Normalization: A Comprehensive Analysis in MCF-7 Breast Cancer Cell Line

Authors: Nityanand Jain, Dina Nitisa, Valdis Pirsko and Inese Cakstina*

*** For Correspondence:**

Laboratory of Molecular Genetics
Institute of Oncology
Riga Stradins University
16 Dzirciema street
Riga
Latvia (LV-1007)

Email: inese.cakstina@rsu.lv

INDEX

1) Extended Discussion – *Sections 1.1 to 1.7*
2) Additional Tables 1-6
3) References

1.1. Quantification Cycle (Cq) and the Need for Lineralization

The Cq (quantification cycle) values (previously referred to as Ct – threshold cycle or Cp – crossing point) represent the number of cycles that are required for the fluorescent signal to cross the threshold. The raw values obtained from and used by the PCR machine are not necessarily suggestive of the absolute quantity of template in terms of intergenic/inter-run comparisons, but in a simplified way, reflects the abundance of the individual template mRNA in wells [1,2]. As Livak et al., described, the raw Cq values are determined from a log linear plot of PCR signal vs the cycle number, thereby making Cq an exponential term rather than a linear term [3]. Ergo, for any gene expression analysis or comparisons it is necessary to convert the Cq values to a linear scale using the 2^{-Cq} method [3].

It is interesting to note that on an initial analysis of Table 1 (*Section 2.1 of main text*), one could argue that the mean Cq values of all genes in both cultures are very comparable to each other and hence the gene expression is stable for all genes in both cultures. However, equal raw Cq values does not necessarily imply that the gene expression is also uniform, it merely implies presence of nearly equal template mRNA in the wells. A 2x-3x fold significant difference between gene expression may still exist once the raw Cq values are converted to a linear scale.

As shown by CV% analysis (Table 2; *Section 2.2 of main text*), the variance between gene expression in both cultures is evident and could otherwise have been overlooked in a case where raw Cq values were only considered. Furthermore, the 2^{-Cq} analysis (Figure 1 and 2) also shows significant fold change for many genes over multiple passages (Supplementary table S1, see Additional file 1). The raw Cq values can, however, give information regarding the intragroup and intergroup variation, as a gene with low variations, both inter- and intra-group would have less Cq dispersion [4]. Also, the use of Cq values can be done on semi-quantitative basis whereby a comparison simply indicates that there were fewer/more copies of gene A than gene B in a well.

1.2. Selecting the Algorithms and Calculations for Validation and Selection of Reference Genes

A pivotal aspect in any gene expression related study is the selection of the most appropriate and accurate reference gene/s. Given the widespread availability of tools and algorithms including web-based platforms (RefFinder) and stand-alone or R-based algorithms (geNorm, BestKeeper, NormFinder etc.), it becomes difficult for researchers without in-depth mathematical background to choose the appropriate software based on their experimental needs.

Each algorithm has been reported to have their own advantages and disadvantages that could render variable results depending on the specific experimental conditions. Considering the limitations and merits of all the algorithms, Sundaram et al. [5], suggested to use an approach that involves using CV% analysis (calculated from 2^{-Cq} linearized values) along with 2^{-Cq} analysis (with calibrator) supported with *ANOVA* (or Kruskal Wallis test) to roughly estimate the stable reference genes. They also suggest using NormFinder to analyze the stability of the genes and conclude the best pair/s of reference genes. Lastly, it was

recommended following the selected pair/s further by analyzing the normalized profile of the target gene/s (gene of interest) to validate the selection.

Since determining suitable reference genes for every sub-clone or sub-type of MCF-7 cell line is not practically and economically viable, we suggest that the strategy outlined by Sundaram et al. [5] can be employed. We suggest supplementing the methodology with TCGA analysis for bolstering the selection.

1.3. Choosing the Workflow for TCGA Analysis

Most samples in TCGA were originally aligned against the Genome Reference Consortium build GRCh37 (hg19) or the "legacy" dataset [6]. However, with advances in technology and drop in sequencing costs, GDC (Genomic Data Commons – conceived by NCI) undertook harmonization efforts to align the data to GRCh38 (hg38) build ("harmonized" dataset). The workflow for generating RNA-Seq data in both legacy and harmonized dataset differs substantially [6] leading to introduction of bias between the hg19 and hg38 abundance estimates. However, Gao et al., [6] demonstrated that excellent concordance exists between the two workflows in relation to BRCA PAM50 subtypes. Furthermore, they reported that relative change between conditions is preserved across all subtypes of BRCA PAM50. Hence, in the present study, the legacy dataset was accessed and analyzed for comparing the expression profiles of the reference genes.

1.4. Normalization in Nutrient Stress

In the present study we compared the fold change in expression of reference genes when the MCF-7 cells are cultured in different growth media, enriched with different supplements. Additional Tables 1-4 enlist the stability ranking of the reference genes in the four stress cultures – B5, D5, E5 and R5, respectively. The least variable genes in these cultures were *CCSER2-PUM1-SF3A1* in B5, *PUM-SF3A1-HNRNPL* in D5, *HSPCB-PGK1-RNA28S* in E5 and *PGK1- HSPCB-SF3A1* in R5. Clearly at the other end of the spectrum, the genes to be constantly ranked most variable included *ACTB* and *GAPDH* in B5, D5 and R5. In E5, *CCSER2* and *HNRNPL* were ranked most variable. The ranking obtained from the nutrient stress shows that the stability of the reference genes is not uniform in varying growth media supplements.

ACTB, a commonly employed reference gene is certainly not the best reference gene in MCF-7 cell line and should not be employed for gene of interest normalization, at least not as a single internal control. It was found to be close to or violating the cutoff for CV% analysis (CV< 50%), rendering it out as a choice for internal control. Our analysis revealed CV% of 48.35 (B5), 58.71 (D5) and 46.12 (R5) for *ACTB*. Furthermore, the fold change for *ACTB* violated the < 2-fold change cutoff in all culturing conditions (Supplementary Table S14; see Additional File 1). *GAPDH*, another commonly used internal control also seems to follow the footsteps of *ACTB*. It violated the CV% cutoff in cultures B5 (51.99) and D5 (58.71) with > 2-fold change in culture E5. Indeed, a strong case for variability of gene expression of *GAPDH* is evident from the present analysis and we strictly advice against using *GAPDH* as a single internal control (unless tested experimentally using comprehensive reference gene matrix).

Although *GAPDH* is not a suitable candidate alone, our analysis in triplet pair of *GAPDH-PCBP1-CCSER2* (as shown in Figure 9), showed successful normalization of fold change in gene expression of both *KRT19* and *AURKA* (genes of interest), indicating potential application of *GAPDH* in association with other reference genes. Furthermore, the normalized fold change for both genes of interest was under the suggested cutoff (< 2) and was found to be statistically non-significant, bolstering the wide range ability of the triplet pair to successfully handle variations in gene expression.

Albeit out of the scope of the present study, a non-exhaustive list of the effects of different supplements on cell growth/ culturing is shown in Additional Table 5 to provide the readers with a broader image of the role these supplements can play in influencing reference gene expression and stability. Further in-depth and comprehensive studies are required to observe the individual effects of these supplements on reference genes (a work in progress by various research groups) to postulate or conclude an explanation for the variance in gene expression obtained in the present study.

1.5. Insight into Other Culturing Conditions (Literature Review)

Parab et al. [7] studied the gene expression of five reference genes in three breast cancer cell lines (MCF-7, MDA-MB-231 and BT474) which underwent progesterone treatment. They concluded *PUM1* and *PSMB2* (Proteosome 20S Subunit β2) as the most stable gene pair across all three cell lines. Use of *PUM1* as an internal control has been recommended by many authors in the past [7-9]. However, it is important to point out that the Parab et al. study lacked control cultures (without progesterone treatment) with differences in growth media and supplements when compared with the present study. Furthermore, the reference genes identified were not employed for normalization of genes of interest. In our analysis, *PUM1* was found to be amongst top 5 when evaluated in culture A1/A2 and was not found to violate the fold change in all nutrient stress cultures (although its fold change between passages in cultures A1/A2 was found to be significant). In our analysis, genes clearly better than *PUM1* had been identified, but we could recommend *PUM1* as another potential candidate (although its stability would need to be validated first before use).

In a different treatment, reference genes were identified in apoptosis induced MCF-7 cells treated with salicylic acid, either in the presence or absence of a heat shock [10]. They identified *HIST* (Histone 2A) and *YWHAZ* (Tyrosine 3-monooxygenase/trytophan 5 monooxygenase activation protein, 14-3-3) as the most stable gene pair. They identified *ACTB* as a third potential candidate however, reported against the use of *GAPDH* as an internal control. Their study did have a negative control (untreated MCF-7 cells), but didn't report on individual ranking (only cumulative ranking was provided). Furthermore, they suggested against *RNA18S* as an internal control, a suggestion that gains our support as well.

Gene expression in Doxorubicin (chemotherapy drug) resistant MCF-7 cells has also been investigated [11]. The researchers found that many phase I/II metabolizing genes (including CYPs) are upregulated along with drug efflux pump genes. Meanwhile, cell cycle associated genes, DNA repair genes and p53 signaling pathway genes were found to be downregulated [11], showcasing the wide spectrum of adaptability shown by MCF-7 cells.

Manipulating the availability of serum for varying time periods is a common laboratory practice in molecular biology. It may serve as the impetus for increasing homogeneity of culture and uniformity of growing conditions when preparing cells for experiment or could constitute an experiment *per se* [12,13]. Hence, effects of FBS (serum) availability (serum free and 10% FBS) on reference gene expression has also been studied in other cell lines like colon adenocarcinoma [13]. The authors concluded *PPIA*/*RPLP0*/*SDHA* (Peptidylprolyl isomerase 1/ Ribosomal protein lateral stalk subunit P0/ Succinate dehydrogenase complex flavoprotein subunit A) as the most stable triplet for normalization in colon adenocarcinoma cell lines whilst ranking *ACTB* and *PGK1* amongst the least stable genes.

Das et al. [14] identified endogenous controls for miRNA quantification in six cancer cell lines including MCF-7 cell line (others included cervical, colon, lung, etc.). They identified *miR-25-3p* as the single most suitable reference gene across the cell lines whilst *miR-25-3p-miR-93-5p* pair was reported as the best endogenous group. In another study Karimi et al. [15] studied the effects of silver nanotoxicity on reference gene expression in MCF-7 cells. They concluded *TBP-GAPDH* (TATA box binding protein) as the most stable pair for normalization of gene expression in MCF-7 cells that underwent silver nanotoxicity. Effects of Melatonin hormone (N-acetyl-5-methoxytryptamine) on MCF-7 cells have also been investigated [16]. *ACTB-GAPDH* pair had been identified as the most stable pair in MCF-7 cells that were treated with melatonin [16].

UBC (Ubiquitin C) and *YWHAZ* have been identified as the most suitable reference genes in MCF-7 cells cultured in both control and treatment conditions (treated with DNA demethylating agent, 5-aza-deoxycytidine, and a histone deacetylase inhibitor, Trichostatin A) [17]. They found that both *GAPDH-ACTB* are not stably expressed in treatment groups. Finally, Lazaro and Kostarelos [18] reported the change in reference gene expression in MCF-7 cells when exposed to graphene oxide sheets. They reported that exposure to graphene oxide sheets altered reference gene expression, including that of *ACTB* and *GAPDH* and concluded with the importance of performing a reference gene matrix analysis that is specifically determined according to defined experimental conditions. These may include, but are not be limited to, physicochemical characteristics of the material, dose, exposure conditions and cell type of interest [18]. However, it is interesting to note that preservation of *GAPDH* expression upon altered conditions has been reported for human umbilical vein endothelial cells (HUVECs) grown under hyperglycemic conditions [19], chondrocytes cultured at different temperatures [20] or blood cells subjected to radiation [21].

1.6. Validation of Expression Fold Change at Different Time Points

To further provide proof of concept, we analyzed the fold change in the expression of select reference genes from our 12 genes in control conditions (A1/A2) using RT-qPCR at different time points and essentially in two different plates (using 2^{-Cq} method; refer to Additional File 5 – Calculations for Section 2.15). Apart from *GAPDH, PCBP1, CCSER2* we analyzed *RNA28S* (because it was one of the stable genes in culture A2), *ACTB* (commonly used reference gene), *RNA18S* and *RPL13A* (both because they were ranked consistently at most variable in cultures A1 and A2). As shown in Additional Table 6, all reference

genes upon repeat qPCR reaction managed to stay below the cutoff of < 2-fold change (illustrating the fact that cDNA isolated was of sufficient quality and preserved appropriately and ergo, the changes in reference gene expression reported didn't arise from methodological errors).

PCBP1 and *CCSER2* showed the least variations in expression fold change (F.C. = 0.812 and 0.931 respectively). *GAPDH* was outranked by other genes – *RNA18S*, *RPL13A* and *ACTB* in both culture A2 and overall MCF-7 cell line however, in culture A2, *GAPDH* was ranked as third least variable gene. Finally, we looked upon the expression fold change of the triplet *GAPDH-PCBP1-CCSER2*. The triplet showed a minimum fold change in expression i.e. 1.01x-fold change which shows that the triplet overall remains very stable in terms of gene expression even if the qPCR reactions are repeated at different time points.

1.7. Generalizability of the Results from Present Study

The present study reports an exhaustive and extensive overview of the stability of reference genes studied over multiple passages in two biological sub-clone (replicate) cultures as well MCF-7 cells cultured in four different nutrient stress conditions. It is evident from prior studies that even the slightest change in culturing conditions can lead to substantial and significant changes in reference gene expression in MCF-7 cell line. Hence, a universal gene pair that is optimized to handle the expression variances in the cell line is yet to be determined and proved. With the present study, *GAPDH-PCBP1-CCSER2* triplet pair proves its utility in optimizing the expression of genes of interest in both control and different nutrient stress environments. Nevertheless, more detailed analysis is required (despite the detailed analysis of the present study) to test the gene pairs in different culturing conditions like hypoxia, melanin exposure, etc. The triplet needs to be tested in established drug resistant MCF-7 sub-lines as well as an inter-laboratory assay to confirm the results from our study. These are also recommended as topics for further exploration. Finally, as stated by Lazaro and Kostarelos [18], "the magnitude and nature of the changes induced (by different culturing conditions) vary between different cell types and therefore reference gene validation cannot be extrapolated but must be specifically determined according to defined experimental conditions" gains our support as well.

Additional Table 1. Comprehensive analysis of candidate reference gene ranking in Culture B5 (Nutrient Stress)

CV%			NormFinder			GeNorm		
**Candidate Gene**	**CV%**	**Rank**	**Candidate Gene**	**Group S.D**	**Rank**	**Candidate Gene**	**Stability value M**	**Rank**
CCSER2	3.552	1	*CCSER2*	0.034	1	*CCSER2*	0.068	1
PUM1	4.788	2	*PUM1*	0.034	1	*PUM1*	0.068	1
SF3A1	5.169	3	*SF3A1*	0.045	2	*SF3A1*	0.083	2
HNRNPL	8.631	4	*HNRNPL*	0.077	3	*HNRNPL*	0.100	3
PCBP1	10.113	5	*PCBP1*	0.134	4	*PCBP1*	0.114	4
RPL13A	11.823	6	*RPL13A*	0.138	5	*RNA18S*	0.148	5
HSPCB	18.615	7	*HSPCB*	0.289	6	*RNA28S*	0.171	6
RNA18S	19.450	8	*RNA18S*	0.289	6	*RPL13A*	0.192	7
RNA28S	20.172	9	*RNA28S*	0.223	7	*HSPCB*	0.238	8
PGK1	21.151	10	*PGK1*	0.314	8	*PGK1*	0.267	9
ACTB	48.383	11	*ACTB*	0.829	9	*ACTB*	0.364	10
GAPDH	51.989	12	*GAPDH*	0.839	10	*GAPDH*	0.448	11

BestKeeper			Comparative ΔCt			RefFinder		
**Candidate Gene**	**S.D**	**Rank**	**Candidate Gene**	**Average S.D**	**Rank**	**Candidate Gene**	**Geomean**	**Rank**
CCSER2	0.041	1	*CCSER2*	0.300	1	*CCSER2*	1.000	1
PUM1	0.051	2	*SF3A1*	0.300	1	*PUM1*	1.860	2
SF3A1	0.060	3	*PUM1*	0.310	2	*SF3A1*	2.710	3
HNRNPL	0.096	4	*PCBP1*	0.330	3	*HNRNPL*	4.230	4
RPL13A	0.099	5	*HNRNPL*	0.330	3	*PCBP1*	4.950	5
PCBP1	0.116	6	*RPL13A*	0.360	4	*RPL13A*	6.610	6
RNA28S	0.188	7	*RNA18S*	0.410	5	*RNA18S*	7.420	7
HSPCB	0.243	8	*RNA28S*	0.410	5	*RNA28S*	7.710	8
RNA18S	0.247	9	*HSPCB*	0.440	6	*HSPCB*	8.210	9
PGK1	0.265	10	*PGK1*	0.460	7	*PGK1*	10.000	10
ACTB	0.665	11	*ACTB*	0.850	8	*ACTB*	11.000	11
GAPDH	0.682	12	*GAPDH*	0.870	9	*GAPDH*	12.000	12

Additional Table 2. Comprehensive analysis of candidate reference gene ranking in Culture D5 (Nutrient Stress)

CV%			NormFinder			geNorm		
**Candidate Gene**	**CV%**	**Rank**	**Candidate Gene**	**Group S.D**	**Rank**	**Candidate Gene**	**Stability value M**	**Rank**
PUM1	3.564	1	*PCBP1*	0.061	1	*HNRNPL*	0.046	1
SF3A1	3.717	2	*SF3A1*	0.062	2	*SF3A1*	0.046	1
HNRNPL	4.654	3	*CCSER2*	0.073	3	*PUM1*	0.058	2
PCBP1	7.712	4	*PUM1*	0.090	4	*PCBP1*	0.092	3
RNA28S	10.741	5	*HNRNPL*	0.094	5	*RNA28S*	0.136	4
CCSER2	11.494	6	*RNA28S*	0.240	6	*RPL13A*	0.158	5
RPL13A	12.125	7	*RPL13A*	0.301	7	*PGK1*	0.180	6
PGK1	16.579	8	*RNA18S*	0.316	8	*HSPCB*	0.193	7
HSPCB	17.284	9	*PGK1*	0.384	9	*CCSER2*	0.208	8
RNA18S	31.062	10	*HSPCB*	0.399	10	*RNA18S*	0.262	9
GAPDH	52.211	11	*GAPDH*	0.735	11	*GAPDH*	0.363	10
ACTB	58.705	12	*ACTB*	0.795	12	*ACTB*	0.441	11

BestKeeper			Comparative ΔCt			RefFinder		
**Candidate Gene**	**S.D**	**Rank**	**Candidate Gene**	**Average S.D**	**Rank**	**Candidate Gene**	**Geomean**	**Rank**
PUM1	0.034	1	*PUM1*	0.310	1	*SF3A1*	1.680	1
SF3A1	0.041	2	*SF3A1*	0.310	1	*PUM1*	1.860	2
HNRNPL	0.053	3	*HNRNPL*	0.310	1	*HNRNPL*	2.570	3
PCBP1	0.082	4	*PCBP1*	0.320	2	*PCBP1*	2.830	4
RNA28S	0.129	5	*CCSER2*	0.340	3	*CCSER2*	5.330	5
CCSER2	0.135	6	*RNA28S*	0.360	4	*RNA28S*	5.480	6
RPL13A	0.145	7	*RPL13A*	0.380	5	*RPL13A*	6.740	7
PGK1	0.212	8	*PGK1*	0.430	6	*PGK1*	7.970	8
HSPCB	0.224	9	*HSPCB*	0.440	7	*HSPCB*	8.970	9
RNA18S	0.356	10	*RNA18S*	0.480	8	*RNA18S*	9.460	10
ACTB	0.734	11	*GAPDH*	0.780	9	*GAPDH*	11.240	11
GAPDH	0.734	11	*ACTB*	0.830	10	*ACTB*	11.740	12

Additional Table 3. Comprehensive analysis of candidate reference gene ranking in Culture E5 (Nutrient Stress)

CV%			NormFinder			geNorm		
**Candidate Gene**	**CV%**	**Rank**	**Candidate Gene**	**Group S.D**	**Rank**	**Candidate Gene**	**Stability value M**	**Rank**
RPL13A	16.378	1	*PGK1*	0.025	1	*PCBP1*	0.051	1
HSPCB	16.937	2	*PCBP1*	0.043	2	*PUM1*	0.051	1
RNA28S	17.234	3	*PUM1*	0.050	3	*PGK1*	0.060	2
GAPDH	17.718	4	*ACTB*	0.058	4	*RNA28S*	0.068	3
PGK1	19.787	5	*RNA28S*	0.065	5	*ACTB*	0.074	4
RNA18S	20.420	6	*SF3A1*	0.097	6	*RPL13A*	0.084	5
PUM1	21.169	7	*HSPCB*	0.102	7	*HSPCB*	0.093	6
ACTB	21.615	8	*RPL13A*	0.108	8	*RNA18S*	0.098	7
PCBP1	22.403	9	*RNA18S*	0.115	9	*SF3A1*	0.107	8
SF3A1	26.201	10	*CCSER2*	0.139	10	*GAPDH*	0.116	9
CCSER2	28.651	11	*GAPDH*	0.142	11	*CCSER2*	0.125	10
HNRNPL	30.186	12	*HNRNPL*	0.185	12	*HNRNPL*	0.137	11

BestKeeper			Comparative ΔCt			RefFinder		
**Candidate Gene**	**S.D**	**Rank**	**Candidate Gene**	**Average S.D**	**Rank**	**Candidate Gene**	**Geomean**	**Rank**
GAPDH	0.201	1	*PGK1*	0.100	1	*PGK1*	2.060	1
HSPCB	0.208	2	*PCBP1*	0.110	2	*PCBP1*	2.450	2
RPL13A	0.210	3	*PUM1*	0.110	2	*PUM1*	2.820	3
RNA28S	0.224	4	*RNA28S*	0.110	2	*RNA28S*	4.230	4
RNA18S	0.241	5	*ACTB*	0.120	3	*HSPCB*	4.920	5
PGK1	0.262	6	*HSPCB*	0.140	4	*ACTB*	5.320	6
PUM1	0.279	7	*SF3A1*	0.140	4	*RPL13A*	5.830	7
ACTB	0.283	8	*RPL13A*	0.140	4	*GAPDH*	5.900	8
PCBP1	0.294	9	*RNA18S*	0.140	4	*RNA18S*	7.540	9
SF3A1	0.346	10	*CCSER2*	0.170	5	*SF3A1*	7.840	10
HNRNPL	0.380	11	*GAPDH*	0.170	5	*CCSER2*	10.720	11
CCSER2	0.381	12	*HNRNPL*	0.200	6	*HNRNPL*	11.740	12

Additional Table 4. Comprehensive analysis of candidate reference gene ranking in Culture R5 (Nutrient Stress)

CV%			NormFinder			GeNorm		
**Candidate Gene**	**CV%**	**Rank**	**Candidate Gene**	**Group S.D**	**Rank**	**Candidate Gene**	**Stability value M**	**Rank**
HSPCB	2.725	1	*PGK1*	0.028	1	*SF3A1*	0.011	1
PGK1	5.855	2	*HSPCB*	0.032	2	*HNRNPL*	0.011	1
RNA28S	9.954	3	*RNA28S*	0.116	3	*PCBP1*	0.048	2
PUM1	11.058	4	*PUM1*	0.178	4	*CCSER2*	0.061	3
SF3A1	13.820	5	*RNA18S*	0.203	5	*PUM1*	0.075	4
HNRNPL	14.104	6	*SF3A1*	0.225	6	*HSPCB*	0.117	5
PCBP1	14.473	7	*HNRNPL*	0.231	7	*PGK1*	0.138	6
RNA18S	15.072	8	*PCBP1*	0.239	8	*RNA28S*	0.170	7
CCSER2	16.024	9	*CCSER2*	0.268	9	*RNA18S*	0.208	8
GAPDH	19.299	10	*GAPDH*	0.278	10	*GAPDH*	0.237	9
RPL13A	19.937	11	*RPL13A*	0.313	11	*RPL13A*	0.263	10
ACTB	46.120	12	*ACTB*	0.634	12	*ACTB*	0.329	11
**BestKeeper**			**Comparative ΔCt**			**RefFinder**		
**Candidate Gene**	**S.D**	**Rank**	**Candidate Gene**	**Average S.D**	**Rank**	**Candidate Gene**	**Geomean**	**Rank**
HSPCB	0.030	1	*HSPCB*	0.240	1	*HSPCB*	1.860	1
PGK1	0.064	2	*PGK1*	0.250	2	*PGK1*	2.300	2
RNA28S	0.090	3	*PUM1*	0.270	3	*SF3A1*	3.500	3
PUM1	0.140	4	*RNA28S*	0.280	4	*PUM1*	3.940	4
SF3A1	0.179	5	*SF3A1*	0.280	4	*HNRNPL*	3.980	5
HNRNPL	0.184	6	*HNRNPL*	0.280	4	*RNA28S*	4.120	6
PCBP1	0.188	7	*PCBP1*	0.290	5	*PCBP1*	5.860	7
RNA18S	0.197	8	*CCSER2*	0.310	6	*CCSER2*	7.140	8
CCSER2	0.206	9	*RNA18S*	0.320	7	*RNA18S*	7.540	9
GAPDH	0.217	10	*GAPDH*	0.370	8	*GAPDH*	10.000	10
RPL13A	0.244	11	*RPL13A*	0.390	9	*RPL13A*	11.000	11
ACTB	0.512	12	*ACTB*	0.660	10	*ACTB*	12.000	12

Additional Table 5. Effects of supplements in cell growth and/or gene expression

Supplement**	Effects in Cell Culture and Growth***	Studied in MCF-7 Cell Line	References
FBS	• Increase in number of viable cells<br>• Drives cells into S phase of cell cycle	Yes	22
Pen/Strep	• Antibiotic effect (to maintain sterility)<br>• Modest decrease in cell viability (≤ 2 FC)<br>• Cytotoxic effect on mammalian cells (even at recommended concentrations)<br>• Affects cell differentiation<br>• Can induce global change in gene expression<br>• Downregulates mainly chaperons, turnover and cytoskeletal proteins<br>• Downregulates *CFL-1* (cofilin) & *ACTB*	Yes	23 – 28
Insulin (I)	• Increases viability, proliferation of cells<br>• Enhances invasion by cells<br>• Upregulates IRS1<br>• Activates RAS/Raf/ERK pathway<br>• Sensitizes cells to cytotoxic therapy<br>• Lowers levels of ERs in cells without affecting binding affinity<br>• Causes loss of nuclear MIER1α (transcriptional tumor repressor)<br>• Stimulates phosphoprotein synthesis<br>• Shortens G_1 phase of cell cycle	Yes<br><br>MCF-7 cell line is dependent on insulin for propagation (Soule et al., 1973)	29 – 36
Hydrocortisone	• Augments insulin effects on total protein and RNA accumulation	Yes	37
Epidermal G.F. (EGF)	• Stimulates cell growth<br>• Increases S-phase recruitment<br>• Increases intracellular GSH	Yes	38 – 45

	• Stimulates DNA synthesis (?); high concentrations shown to inhibit DNA synthesis completely<br>• Induces cell motility<br>• Induces multi-drug resistance<br>• Upregulates pS2 and Cathepsin-D gene expression (*TFF1* and *CTSD* respectively)<br><br>***Long-term exposure effects (in-vitro) of EGF and/or 17β estradiol (E2):***<br>• Enhanced cell proliferation<br>• Mammo-sphere formation<br>• Increased CD44+/CD24- cell ratio<br>• Altered responses to short term treatment with EGF, E2 etc.		
17β – Estradiol	• Direct mitogenic effect on MCF-7 cells<br>• Responsiveness dependent on secretion of autocrine factor activating IGF-IR<br>• Strain dependent response<br>• Induces proliferation and migration<br>• Attenuates expression of mfn2 (mitofusion 2)	Yes	46 – 50
HEPES	• Is up taken in intracellular compartment<br>• Removal can require > 4 days from intracellular compartment<br>• Can alter intracellular pH (?)<br>• Can be used for enhancing protein transfection<br>• Compensates reduced buffer capacity in low serum media<br>• Independent of CO2 atmosphere<br><br>***Adverse effects (not investigated in MCF-7):***<br>• Forms H2O2 on light exposure<br>• Stimulates cytotoxic O2 metabolites formation	Yes	51 – 60

	• Can interact with anion channels<br>• Induces vacuolization<br>• Activates lysosomal transcription factors		
Transferrin	• Stimulatory effects (although less pronounced when compared with insulin)<br>• Stimulatory effect may be due to hormonal contaminant in preparations of transferrin (?)	Yes	61 – 62
T3	• Stimulates cell proliferation (?)<br>• Dose dependent effects<br>• Potentiates effects of E2<br>• Has been shown to induce apoptosis in MCF-7 cells by repressing *SMP30* gene<br>• Upregulates expression of *TGFA* and *HIF1A*	Yes	63 – 68
Se	• Inhibits cell growth and proliferation<br>• Low conc. – increases level of antioxidant GSH<br>• High conc. – increases level of ROS<br>• Pro-oxidant catalyst<br>• Can cause beclin-1 transcriptional inhibition linked to HSP90 and nuclear factor kappa B<br>• Decreases amount and mRNA of ER-α<br>• Increases conc. of PR and pS2 mRNA expression	Yes	69 – 74
Cholera toxin	• Increases intracellular cAMP<br>• Enhanced cellular proliferation (?)<br>• Synergism with 17β-Estradiol (?)	Yes	75 – 76

* (?) – indicates conflicting or unproved hypothesis/reports in the literature. **FBS – Fetal Bovine Serum; Pen/Strep – Penicillin/Streptomycin; Epidermal G.F. – Epidermal Growth Factor; HEPES - (4-(2-hydroxyethyl)-1-piperazineethanesulfonic acid); T3 - 3,3′,5-triiodo-l-thyronine; Se—sodium selenite. ***FC – fold change; IRS1 – Insulin Receptor Substrate 1; ERs – Estrogen Receptor; GSH – Glutathione; SMP30 – Senescence marker protein 30 ; TGFA – transforming growth factor α; HIF1A – Hypoxia inducing factor; ROS – reactive oxygen species; PR- progesterone receptor

Additional Table 6. Expression fold change of select reference genes when qPCR reaction is repeated at different time point

Reference gene	Culture A1		Culture A2		MCF-7 Cell Line (A1+A2)	
	**Expression fold change**	**Ranking**	**Expression fold change**	**Ranking**	**Expression fold change**	**Ranking**
ACTB	1.331	5	1.263	4	1.291	4
GAPDH	1.565	7	1.198	3	1.316	6
CCSER2	0.799	1	1.040	2	0.931	2
PCBP1	1.006	2	0.719	1	0.812	1
RNA18S	1.124	3	1.286	6	1.208	3
RNA28S	1.482	6	1.552	7	1.523	7
RPL13A	1.315	4	1.280	5	1.292	5
GAPDH/ CCSER2/ PCBP1	1.077	-	0.972	-	1.010	-

2. *References*

1. Kosuth J., Farkasovska M., Mochnacky F., Daxnerova Z. and Sevc J. Selection of Reliable reference genes for analysis of gene expression in spinal cord during rat postnatal development and after injury. Brain Sci. December 2019. 10(1):6. Doi: 10.3390/brainsci10010006.
2. Boda E., Pini A., Hoxha E., Parolisi R and Tempai F. Selection of reference genes for quantitative real time RT-PCR studies in mouse brain. J Mol Neurosci. Humana Press Inc. 2009. 37:238-253. PMID:18607772.
3. Livak K-J. and Schmittgen T-D. Analysis of Relative gene expression data using real-time quantitative PCR and the $2^{-\Delta\Delta CT}$ method. Methods. 2001. 25; 402-408. DOI: 10.1006/meth.2001.1262.
4. Piazza V.G., Bartke A., Miquet J.G. and Sotelo A.I. Analysis of Different approaches for the selection of reference genes in RT-qPCR experiments: A case study in skeletal muscle of growing mice. Int J Mol Sci. May 2017. 18(5):1060. Doi: 10.3390/ijms18051060. PMID: 28509880.
5. Venkat K.S, Nirmal K.S., Charbel M., Julien G. Optimal Use of statistical methods to validate reference gene stability in longitudinal studies. Plos One. 2019. 14(7): e0219440.
6. Gao G.F., Parker JS., Reynolds SM., Silva TC., Wang LB., Zhou W., Akbani R., et al. Before and After: Comparison of Legacy and harmonized TCGA Genomic Data Common's data. Cell Systems. July 2019. 9(1):24-34. e10.
7. Parab, Aniket. and Kambli, Jaykumar. and Hake, Sujata. and Joshi, Narendra. Evaluation of housekeeping genes for studies in breast cancer cell lines treated with progesterone. Biomedical Research Journal. 2018; 5(1): 6-21. Doi: 10.4103/2349-3666.240301.
8. Bhat S, Gardi N, Hake S, Kotian N, Sawant S, Kannan S. Impact of intra-tumoral IL17A and IL32 gene expression on T-cell responses and lymph node status in breast cancer patients. J Cancer Res Clin Oncol 2017;143(9):1745–1756.
9. Lyng MB, Laenkholm AV, Pallisgaard N, Ditzel HJ. Identification of genes for normalization of real-time RT-PCR data in breast carcinomas. BMC Cancer 2008;8:20.
10. Ferreira, E., Cronjé, M.J. Selection of Suitable Reference Genes for Quantitative Real-Time PCR in Apoptosis-Induced MCF-7 Breast Cancer Cells. Mol Biotechnol. 2012; 50: 121–128. https://doi.org/10.1007/s12033-011-9425-3.
11. AbuHammad, S., & Zihlif, M. Gene expression alterations in doxorubicin resistant MCF7 breast cancer cell line. Genomics. 2013; 101(4), 213–220. doi:10.1016/j.ygeno.2012.11.009
12. Pirkmajer S, Chibalin AV. Serum starvation: caveat emptor. Am J Physiol Cell Physiol; 2011; 301:C272–C279.
13. Krzystek-Korpacka, M., Hotowy, K., Czapinska, E. et al. Serum availability affects expression of common house-keeping genes in colon adenocarcinoma cell lines: implications for quantitative real-time PCR studies. Cytotechnology. 2016; 68: 2503–2517. https://doi.org/10.1007/s10616-016-9971-4.
14. Das, Mrinal and Andreassen, Rune and Haugen, Trine and Furu, Kari. Identification of Endogenous Controls for Use in miRNA Quantification in Human Cancer Cell Lines. Cancer genomics and Proteomics. 2015; 13(1):63-68.
15. Karimi, Z., Hashemi, A. Validation of Reference Genes for Silver Nanotoxicity Analysis in MCF-7 Cell Line. J Clust Sci. 2018; 29: 159–166. https://doi.org/10.1007/s10876-017-1313-y.
16. Donmez H., Kocak N., Hitit M., Celik F-S., et al. Selection of Reliable Reference Genes for qPCR Analysis on MCF7 Cells with Melatonin treated. Poster presented at: 7th International Molecular Biology and Biotechnology Congress. April 2018.
17. Chua, S.L., See Too, W.C., Khoo, B.Y. et al. UBC and YWHAZ as suitable reference genes for accurate normalisation of gene expression using MCF7, HCT116 and HepG2 cell lines. Cytotechnology. 2011; 63: 645–654. https://doi.org/10.1007/s10616-011-9383-4.
18. de Lázaro, I., Kostarelos, K. Exposure to graphene oxide sheets alters the expression of reference genes used for real-time RT-qPCR normalization. Sci Rep. 2019; 9: 12520. https://doi.org/10.1038/s41598-019-48970-z.

19. Bakhashab S, Lary S, Ahmed F, Schulten HJ, Bashir A, Ahmed FW, Al-Malki AL, Jamal HS, Gari MA, Weaver JU. Reference genes for expression studies in hypoxia and hyperglycemia models in human umbilical vein endothelial cells. G3 (Bethesda), 2014; 4:2159–2165.
20. Ito A, Aoyama T, Tajino J, Nagai M, Yamaguchi S, Iijima H, Zhang X, Akiyama H, Kuroki H. Evaluation of reference genes for human chondrocytes cultured in several different thermal environments. Int J Hyperthermia. 2014; 30:210–216.
21. Vaiphei ST, Keppen J, Nongrum S, Chaubey RC, Kma L, Sharan RN. Evaluation of endogenous control gene(s) for gene expression studies in human blood exposed to 60Co γ-rays ex vivo. J Radiat Res. 2015; 56:177–185.
22. Teo, G.-Y., Rasedee, A., AL-Haj, N. A., Yee Beh, C., Wun How, C., Sulaiman Rahman, H., et al. Effect of fetal bovine serum on erythropoietin receptor expression and viability of breast cancer cells. Saudi Journal of Biological Sciences. 2020; 27(2):653-658. doi:10.1016/j.sjbs.2019.11.032.
23. Martinez-Liarte, J. H., Solano, F., & Lozano, J. A. Effect of Penicillin-Streptomycin and Other Antibiotics on Melanogenic Parameters in Cultured B16/F10 Melanoma Cells. Pigment Cell Research. 1995; 8(2): 83–88. doi:10.1111/j.1600-0749.1995.tb00646.x.
24. Ryu, A.H., Eckalbar, W.L., Kreimer, A. et al. Use antibiotics in cell culture with caution: genome-wide identification of antibiotic-induced changes in gene expression and regulation. Sci Rep. 2017; 7: 7533. https://doi.org/10.1038/s41598-017-07757-w.
25. Llobet L, Montoya J, López-Gallardo E, Ruiz-Pesini E. Side Effects of Culture Media Antibiotics on Cell Differentiation. Tissue Eng Part C Methods. 2015 Nov;21(11):1143-7. doi: 10.1089/ten.TEC.2015.0062. Epub 2015 Jul 8. PMID: 26037505.
26. Schwarze PE, Seglen PO. Effects of antibiotics on protein synthesis and degradation in primary cultures of rat hepatocytes. In Vitro. 1981 Jan;17(1):71-6. doi: 10.1007/BF02618033. PMID: 7216241.
27. Goldstein AL, Johnson PR. Primary culture of adipoblasts from obese and lean Zucker rat adipose tissue. Metabolism: Clinical and Experimental. 1982 Jun;31(6):601-607. DOI: 10.1016/0026-0495(82)90099-3.
28. Mathieson W, Kirkland S, Leonard R, Thomas GA. Antimicrobials and in vitro systems: antibiotics and antimycotics alter the proteome of MCF-7 cells in culture. J Cell Biochem. 2011 Aug;112(8):2170-8. doi: 10.1002/jcb.23143. PMID: 21480367.
29. Wei ML, Duan P, Wang ZM, Ding M, Tu P. High glucose and high insulin conditions promote MCF 7 cell proliferation and invasion by upregulating IRS1 and activating the Ras/Raf/ERK pathway. Mol Med Rep. 2017 Nov;16(5):6690-6696. doi: 10.3892/mmr.2017.7420. Epub 2017 Aug 31. PMID: 28901503; PMCID: PMC5865785.
30. Chappell J., Leitner J.W., Solomon S., Golovchenko I., Goalstone M.L., and Draznin B. Effect of Insulin on Cell Cycle Progression in MCF-7 Breast Cancer Cells DIRECT AND POTENTIATING INFLUENCE. The Journal of Biological Chemistry. 2001; 276: 38023-38028.
31. Agrawal S, Łuc M, Ziółkowski P, Agrawal AK, Pielka E, Walaszek K, Zduniak K, Woźniak M. Insulin-induced enhancement of MCF-7 breast cancer cell response to 5-fluorouracil and cyclophosphamide. Tumour Biol. 2017 Jun;39(6):1010428317702901. doi: 10.1177/1010428317702901. PMID: 28631569.
32. Butler W-B., Kelsey W-H., and Goran N. Effects of Serum and Insulin on the Sensitivity of the Human Breast Cancer Cell Line MCF-7 to Estrogen and Antiestrogens. Cancer Res. 1981; 41:82-88.
33. Li, S., Paterno, G.D. & Gillespie, L.L. Insulin and IGF-1, but not 17β-estradiol, alter the subcellular localization of MIER1α in MCF7 breast carcinoma cells. BMC Res Notes. 2015; 8: 356. https://doi.org/10.1186/s13104-015-1336-0.
34. Linebaugh, B. E., & Rillema, J. A. Actions of insulin on MCF-7 cells that are synchronized with hydroxyurea. Molecular and Cellular Endocrinology. 1987; 52(3): 227–233. doi:10.1016/0303-7207(87)90048-7.
35. Ashihara, T. and Baserga, R. In: Methods in Enzymology, Vol. 58, Eds.: W.B. Jakoby and J.H. Pastan (Academic Press, New York) 1979; pp. 248-262.
36. Soule HD, Vazguez J, Long A, Albert S, Brennan M. A human cell line from a pleural effusion derived from a breast carcinoma. J Natl Cancer Inst. 1973 Nov;51(5):1409-16. doi: 10.1093/jnci/51.5.1409. PMID: 4357757.
37. Linebaugh B.E., and Rillema J.A. Hydrocortisone enhancement of insulin's action on macromolecular synthesis in MCF-7 cells. Mol and Cell Endocrinology. 1977; 7(4):335-343. Doi: https://doi.org/10.1016/0303-7207(77)90034-X.

38. Wollman, R., Yahalom, J., Mary, R., Pinto, J., & Fuks, Z. Effect of epidermal growth factor on the growth and radiation sensitivity of human breast cancer cells in vitro. International Journal of Radiation Oncology*Biology*Physics. 1994; 30(1): 91–98. doi:10.1016/0360-3016(94)90523-1.
39. Fitzpatrick, S. L.: LaChance, M. P.: Schultz, G. S. Characterization of epidermal growth factor receptor and action on human breast cancer cells in culture. Cancer Res. 1984; 44: 3442-3447
40. Karey, K. P.: Sirbasku. D. A. Differential responsiveness of human breast cancer cell lines MCF-7 and T47D to growth factors and l7B-estradiol. Cancer Res. 1988; 48:4083-409.
41. Osborne, C. K.; Hamilton, B.: Titus, G.; Livingston, R. B. Epidermal growth factor stimulation of human breast cancer cells in culture. Cancer Res. 1980; 40:236 l-2366.
42. Cunha, S. I., Jia, M., Souchelnytskyi, S. Exposure to EGF and 17β-estradiol irreversibly affects the proliferation and transformation of MCF7 cells but is not sufficient to promote tumor growth in a xenograft mouse model upon withdrawal of exposure. Int J of Mol Medicine. 2018; 42(3): 1615-1624. Doi: 10.3892/ijmm.2018.3737.
43. Imai Y., Leung C-KH., Friesen H-G., and Shiu R-PC. Epidermal Growth Factor Receptors and Effect of Epidermal Growth Factor on Growth of Human Breast Cancer Cells in Long-Term Tissue Culture. Cancer Res. 1982; 42:4394-4398.
44. Ruben Garcia, Richard A. Franklin & James A. McCubrey. EGF Induces Cell Motility and Multi-Drug Resistance Gene Expression in Breast Cancer Cells, Cell Cycle, 2006; 5(23): 2820-2826. DOI: 10.4161/cc.5.23.3535.
45. Cavailles V., Garcia M., and Rochefort H. Regulation of Cathepsin-D and pS2 Gene Expression by Growth Factors in MCF7 Human Breast Cancer Cells. Molecular Endocrinology. 1989; 3(3):552-558.
46. Furuya Y., Kohno N., Fujiwara Y., and Saitoh Y. Mechanisms of Estrogen Action on the Proliferation of MCF-7 Human Breast Cancer Cells in an Improved Culture Medium. Cancer Res. 1989; 49: 6670-6674.
47. Hamelers IH, Van Schaik RF, Sussenbach JS, Steenbergh PH. 17beta-Estradiol responsiveness of MCF-7 laboratory strains is dependent on an autocrine signal activating the IGF type I receptor. Cancer Cell Int. 2003 Jul 11;3(1):10. doi: 10.1186/1475-2867-3-10. PMID: 12890289; PMCID: PMC169177.
48. Ma, L., Liu, Y., Geng, C., Qi, X., Jiang, J.Estrogen receptor β inhibits estradiol-induced proliferation and migration of MCF-7 cells through regulation of mitofusin 2 Corrigendum in /10.3892/ijo.2016.3695. International Journal of Oncology 2013; 42(6): 1993-2000. https://doi.org/10.3892/ijo.2013.1903.
49. Hyeseong Cho, Peter A. NG, and Katzenellenbogen B-S. Differential Regulation of Gene Expression by Estrogen in Estrogen Growth-Independent and -Dependent MCF-7 Human Breast Cancer Cell Sublines. Molecular Endocrinology. 1991; 5(9): 1323-1330.
50. Liao XH, Lu DL, Wang N, et al. Estrogen receptor α mediates proliferation of breast cancer MCF-7 cells via a p21/PCNA/E2F1-dependent pathway. The FEBS Journal. 2014 Feb;281(3):927-942. DOI: 10.1111/febs.12658.
51. Depping, R., & Seeger, K. 1H-NMR spectroscopy shows cellular uptake of HEPES buffer by human cell lines—an effect to be considered in cell culture experiments. Analytical and Bioanalytical Chemistry. 2018; 411(4): 797–802. doi:10.1007/s00216-018-1518-4.
52. Chen S-H., Chao A., Tsai C-L., et al. Utilization of HEPES for Enhancing Protein Transfection into Mammalian Cells. Molecular therapy: Methods and Clinical Development. 2019; 13: 99-111. Doi: 10.1016/j.omtm.2018.12.005.
53. Shipman C. Evaluation of 4-(2-hydroxyethyl)-1- piperazineëthanesulfonic acid (HEPES) as a tissue culture buffer. Proc Soc Exp Biol Med. 1969;130(1):305–10. https://doi.org/10. 3181/00379727-130-33543.
54. Zigler JS, Lepe-Zuniga JL, Vistica B, Gery I. Analysis of the cytotoxic effects of light-exposed hepes-containing culture medium. In Vitro Cellular and Developmental Biology. 1985;21(5):282–7. https://doi.org/10.1007/BF02620943.
55. Bowman CM, Berger EM, Butler EN, Toth KM, Repine JE. HEPES may stimulate cultured endothelial cells to make growthretarding oxygen metabolites. In Vitro Cellular and Developmental Biology. 1985;21(3):140–2. https://doi.org/10.1007/BF02621350.
56. Hanrahan JW, Tabcharani JA. Inhibition of an outwardly rectifying anion channel by HEPES and related buffers. J Membr Biol. 1990;116(1):65–77. https://doi.org/10.1007/bf01871673.

57. Yamamoto D, Suzuki N. Blockage of chloride channels by HEPES buffer. Proc R Soc Lond Ser B Biol Sci. 1987;230(1258):93–100. https://doi.org/10.1098/rspb.1987.0011.
58. Poole CA, Reilly HC, Flint MH. The adverse effects of HEPES, TES, and BES zwitterion buffers on the ultrastructure of cultured chick embryo epiphyseal chondrocytes. In Vitro. 1982;18(9):755– 65. https://doi.org/10.1007/bf02796499.
59. Verdery RB, Nist C, Fujimoto WY, Wight TN, Glomset JA. Reversible ultrastructural changes in human fibroblasts grown in hepes buffered MCDB-104 supplemented with human serum. In Vitro. 1981;17(11):956–64. https://doi.org/10.1007/ bf02618420.
60. Tol MJ, van der Lienden MJC, Gabriel TL, Hagen JJ, Scheij S, Veenendaal T, et al. HEPES activates a MiT/TFE-dependent lysosomal-autophagic gene network in cultured cells: a call for caution. Autophagy. 2018;14(3):437–49. https://doi.org/10.1080/ 15548627.2017.1419118.
61. Barnes, D., & Sato, G. Methods for growth of cultured cells in serum-free medium. Analytical Biochemistry, 1980; 102(2): 255–270. doi:10.1016/0003-2697(80)90151-7.
62. Barnes, D., & Sato, G. Serum-free cell culture: a unifying approach. Cell. 1980; 22(3): 649–655. doi:10.1016/0092-8674(80)90540-1.
63. Burke R-E., and McGuire W-L. Nuclear Thyroid Hormone Receptors in a Human Breast Cancer Cell Line. Cancer Res. 1978; 38:3769-3773.
64. Hall LC, Salazar EP, Kane SR, Liu N. Effects of thyroid hormones on human breast cancer cell proliferation. J Steroid Biochem Mol Biol. 2008 Mar;109(1-2):57-66. doi: 10.1016/j.jsbmb.2007.12.008. Epub 2007 Dec 7. PMID: 18328691.
65. Sar P, Peter R, Rath B, Mohapatra AD, Mishra SK. 3, 3′5 Triiodo L Thyronine Induces Apoptosis in Human Breast Cancer MCF-7cells, Repressing SMP30 Expression through Negative Thyroid Response Elements. PLOS ONE 2011; 6(6): e20861. https://doi.org/10.1371/journal.pone.0020861.
66. Silva, Tabata M., Moretto, Fernanda C. F., Sibio, Maria T. De, et al. Triiodothyronine (T3) upregulates the expression of proto-oncogene TGFA independent of MAPK/ERK pathway activation in the human breast adenocarcinoma cell line, MCF7. Archives of Endocrinology and Metabolism, 2019; 63(2): 142-147. Epub March 21, 2019. https://dx.doi.org/10.20945/2359-3997000000114.
67. Moretto, F. C. F., De Sibio, M. T., Luvizon, A. C., Olimpio, R. M. C., de Oliveira, M., et al. Triiodothyronine (T 3) induces HIF1A and TGFA expression in MCF7 cells by activating PI3K. Life Sciences. 2016; 154: 52–57. doi:10.1016/j.lfs.2016.04.024.
68. Zhang, L., Zhang, F., Li, Y. et al. Triiodothyronine Promotes Cell Proliferation of Breast Cancer via Modulating miR-204/Amphiregulin. Pathol. Oncol. Res.2019; 25: 653–658. https://doi.org/10.1007/s12253-018-0525-2.
69. Jiang Q, Wang Y, Li T, Shi K, Li Z, Ma Y, Li F, Luo H, Yang Y, Xu C. Heat shock protein 90-mediated inactivation of nuclear factor-κB switches autophagy to apoptosis through becn1 transcriptional inhibition in selenite-induced NB4 cells. Mol Biol Cell. 2011 Apr 15;22(8):1167-80. doi: 10.1091/mbc.E10-10-0860. Epub 2011 Feb 23. PMID: 21346199; PMCID: PMC3078072.
70. Spallholz J. E. On the nature of selenium toxicity and carcinostatic activity. Free Radical Biology and Medicine. 1994;17(1):45–64. doi: 10.1016/0891-5849(94)90007-8.
71. Spallholz J. E. Free radical generation by selenium compounds and their prooxidant toxicity. Biomedical and Environmental Sciences. 1997;10(2-3):260–270.
72. Lipinski B. Rationale for the treatment of cancer with sodium selenite. Medical Hypotheses. 2005;64(4):806–810. doi: 10.1016/j.mehy.2004.10.012.
73. Badr DM, Hafez HF, Agha AM, Shouman SA. The Combination of α-Tocopheryl Succinate and Sodium Selenite on Breast Cancer: A Merit or a Demerit? Oxid Med Cell Longev. 2016;2016:4741694. doi: 10.1155/2016/4741694. Epub 2016 Mar 29. PMID: 27127548; PMCID: PMC4834195.
74. Stoica A, Pentecost E, Martin MB. Effects of selenite on estrogen receptor-alpha expression and activity in MCF-7 breast cancer cells. J Cell Biochem. 2000 Aug 2;79(2):282-92. doi: 10.1002/1097-4644(20001101)79:2<282::aid-jcb110>3.0.co;2-v. PMID: 10967555.

75. Sheffield, L. G., & Welsch, C. W. Cholera-toxin-enhanced growth of human breast cancer cell lines in vitro and in vivo: Interaction with estrogen. International Journal of Cancer. 1985;36(4):479–483. doi:10.1002/ijc.2910360411.
76. Cho-Chung, Y.S., Clair, T., Shepheard, C., and Berhoffer, B., Arrest of hormone-dependent mammary cancer growth in vivo and in vitro by cholera toxin. Cancer Res. 1983; 43:1473-1476.
