# Supplementary material for: Selecting suitable reference genes for qPCR normalization: a comprehensive analysis in MCF-7 breast cancer cell line
Source: BMC Mol Cell Biol. 2020 Sep 25;21:68. doi: 10.1186/s12860-020-00313-x (PMC7519550; doi:10.1186/s12860-020-00313-x)
Supplement: Supplementary file 4 — Additional file 4: Primer Design & Sequence. [file 12860_2020_313_MOESM4_ESM.pdf]

ADDITIONAL FILE 4: PRIMER DESIGN AND SEQUENCE

Selecting Suitable Reference Genes for qPCR Normalization: A Comprehensive Analysis in MCF-7 Breast Cancer Cell Line

Authors: Nityanand Jain, Dina Nitisa, Valdis Pirsko and Inese Cakstina*

*** For Correspondence:**

Laboratory of Molecular Genetics
Institute of Oncology
Riga Stradins University
16 Dzirciema street
Riga
Latvia (LV-1007)

Email: inese.cakstina@rsu.lv

INDEX

1) Reference Gene Selection Criteria
2) Genes of Interest Selection Criteria
3) Additional Table 1 – Description of selected reference genes and genes of interest
4) Additional Table 2 – Primer sequences of selected reference genes and genes of interest
5) Additional Figures P1 – P14 – Melting curves of reference genes and genes of interest
6) References

REFERENCE GENE SELECTION CRITERIA

GAPDH and *ACTB* are two of the most used single control genes reported in more than 90% of the cases in high impact journals [1]. Further these two genes are commonly included in the commercially available cancer pathway kits like those from Qiagen, Life technologies and are also included in Oncotype DX test arrays. Life technologies kit also includes genes like *RNA18S* and *PGK1* along with several others [2]. *RNA18S-ACTB* has also been identified as an appropriate gene pair across all breast cancer cell lines [3]. *RPL13A* was described by De Jonge et al., as a novel candidate reference gene with enhanced stability among a magnitude of different cell types across varying experimental conditions [4]. *HSPCB* has been reported previously to be one of the most stable reference gene for ER+ breast cancer cells [3,5], thereby influencing its selection in the present study.

PUM 1 and *CCSER2* were identified by Tilli et al., using transcriptomic analysis across breast cancer cell lines as less variable and more accurate for research in breast cancer cell lines and tissue samples in comparison to the traditional housekeeping genes [2]. Previously, *HNRNPL* and *PCBP1* have been reported to be among the most highly expressed genes across breast cancer samples and performed better than both *ACTB* and *GAPDH* as supported by TCGA transcriptomic validation reported by Jo et al. [6]. *ATCB* and *SF3A1* were identified by using high throughput analysis of micro-assay datasets with subsequent validation by RT-qPCR as reported by Maltseva et al. [7]. They further reported these genes as more efficient for analysis of breast cancer samples when compared with the reference gene panel provided by Oncotype DX assay. Finally. *RNA28S* was added to the selected genes as an experimental in-house suggestion.

GENES OF INTEREST SELECTION CRITERIA

AURKA (Aurora Kinase A) has been known to be associated with playing a key role in centrosome duplication and chromosome segregation during mitosis [8]. Further, it has been reported at many instances to be amplified/mutated in several human cancers [9,10,11,12,13,14]. In breast cancer, mixed evidence has been reported with some reporting its association with basal like phenotype [15-16] whilst others suggesting it to be a marker for progression and outcome of luminal like subtype [17]. Keeping this in mind *AURKA* made a perfect candidate as gene of interest. Although when reference genes were taken in pairs of two, only *GAPDH-CCSER2* normalized *AURKA* expression in culture A2, it was adequately normalized by *GAPDH-CCSER2-PCBP1* triplet in both the cultures A1 and A2.

The other gene of interest selected was *KRT19* (Keratin 19). It belongs to the *KRT* (keratin) family which serves as important markers in RT-qPCR mediated detection of tumors in lymph nodes, peripheral blood and bone marrows of breast cancer patients [18]. *KRT19* is one of the smallest intermediate filament KRT protein [19] and has been shown to regulate breast cancer properties [20]. Using Oncomine database and RT-PCR, Saha et al. [21] evaluated expression of *KRT19* in MCF-7 and other breast cancer cell lines. They reported that *KRT19* was significantly overexpressed in MCF-7, MDA-MB-231 and SKBR3, ergo validating its choice in the present study. None of the reference gene pairs in two cultures could normalize *KRT19* when they were employed in groups of 2. However, in both the cultures, only *GAPDH-PCBP1-CCSER2* yielded successful normalization thereby proving the ability of the triplet pair to handle genes of interest.

Additional Table 1. Description of the selected candidate Reference genes and Genes of Interest for the study

Gene Symbol	Gene Name	Molecular Function	Accession Number	Chromosomal Localisation
ACTB	β – Actin	Cytoskeleton (Contractile apparatus)	NM_001101	7p22 – p12
GAPDH	Glyceraldehyde 3-phosphate dehydrogenase	Glycolytic enzyme	NM_002046	12p13.31
RPL13A	Ribosomal protein L13a	Ribosome subunit, translation	NM_012423	19q13.33
PGK1	Phosphoglycerate kinase 1	Glycolytic enzyme	NM_000291	Xp21.1
HSPCB	Heat Shock protein 90kDa beta	Signal transduction, Protein folding	NM_007355	6p21.1
RNA28S	28S ribosomal RNA	Ribosome subunit, translation	NR_003287	Unknown
RNA18S	18S ribosomal RNA	Ribosome subunit, translation	NR_003286	Unknown
PUM1	Pumilio RNA binding family member 1	RNA binding protein encoding	NM_001020658	1p35.2
CCSER2	Coiled Coil Serine Rich protein 2	Microtubule binding protein encoding	NM_018999	10q23.1
HNRNPL	Heterogenous Nuclear Ribonucleoprotein L	Formation, processing & packaging of mRNA	NM_001005335	19q13.2
PCBP1	Poly (rC) Binding Protein 1	RNA binding protein encoding	NM_006196	2p13.3
SF3A1	Splicing Factor 3a Subunit 1	Spliceosome assembly & pre-mRNA splicing	NM_005877	22q12.2
AURKA*	Aurora Kinase A	Mitotic centrosomal protein kinase (controls chromosome segregation during mitosis)	NM_003600	20q13.2
KRT19*	Keratin 19	Structural molecule and constituent of cytoskeleton	NM_002276	17q21.2

*Genes that were used as gene of interest for normalization by candidate reference genes.

Additional Table 2. Primers for the selected candidate genes and Genes of Interest

Gene Symbol	Primer Pair (F - Forward; R – Reverse)	Amplicon Length (bp)	Annealing Temperature (^{0}C)	Primer Reference
ACTB	F: 5`- CACCATTGGCAATGAGCGGTTC - 3′<br>R: 3`- AGGTCTTTGCGGATGTCCACGT – 5`	135	58	[3]
GAPDH	F: 5`- GACAGTCAGCCGCATCTTCT - 3`<br>R: 3`- TTAAAAGCAGCCCTGGTGAC - 5`	127	58	[3]
RPL13A	F: 5`- CTATGACCAATAGGAAGAGCAACC - 3`<br>R: 3`- GCAGAGTATATGACCAGGTGGAA – 5`	121	58	[22]
PGK1	F: 5`- CTTAAGGTGCTCAACAACATGG - 3`<br>R: 3`- ACAGGCAAGGTAATCTTCACAC - 5`	119	58	Present Study
HSPCB	F: 5`- CTCTGTCAGAGTATGTTTCTCGC - 3`<br>R: 3`- GTTTCCGCACTCGCTCCACAAA - 5`	114	58	[3]
RNA28S	F: 5`- CAGGGGAATCCGACTGTTTA - 3`<br>R: 3`- ATGACGAGGCATTTGGCTAC - 5`	174	58	Present Study
RNA18S	F: 5`- CGGCTACCACATCCAAGGAA - 3`<br>R: 3`- GCTGGAATTACCGCGGCT – 5`	187	58	[23]
PUM1	F: 5`- AGTGGGGGACTAGGCGTTAG - 3`<br>R: 3`- GTTTTCATCACTGTCTGCATCC -5`	111	58	[24]
CCSER2	F: 5`- GACAGGAGCATTACCACCTCAG - 3`<br>R: 3`- CTTCTGAGCCTGGAAAAAGGGC – 5`	143	58	[2]
HNRNPL	F: 5`- ACAAACCCCAATCTCAGTGG - 3`<br>R: 3`- CCCTCATCATGGTAATGGCT – 5`	140	58	[6]

PCBP1	F: 5`- TGATCATCGACAAGCTGGAG - 3`<br>R: 3`- TCTTTGATCTTACACCCGCC – 5`	145	58	[6]
SF3A1	F: 5`- AAGGGTCCAGTGTCCATCAAAGT – 3`<br>R: 3`- GCCATGTTGTAGTAAGCCAGTGAG – 5`	224	58	[7]
AURKA*	F: 5′- GGAGCCTTGGAGTTCTTTGC – 3′<br>R: 3'- CCTGGCTCCCTCTGTTACAA – 5′	134	58	Present Study
KRT19*	F: 5′- GAGCATGAAAGCTGCCTTGG – 3′<br>R: 3′ – CTGGGCTTCAATACCGCTGA – 5′	103	58	Present Study

* Genes that were used as Gene of Interest for validation of Candidate Reference genes.

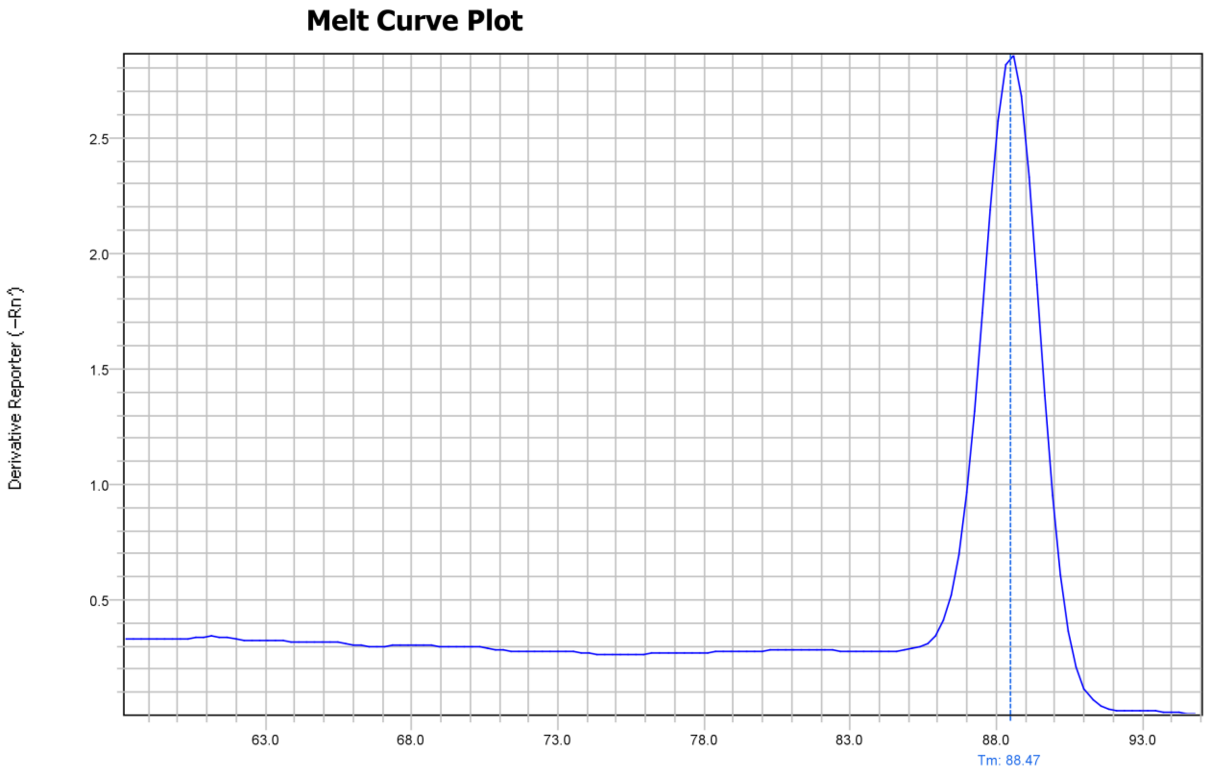

Additional Figure P1. Single, amplicon from *ACTB* reveals a single peak following melting curve analysis.

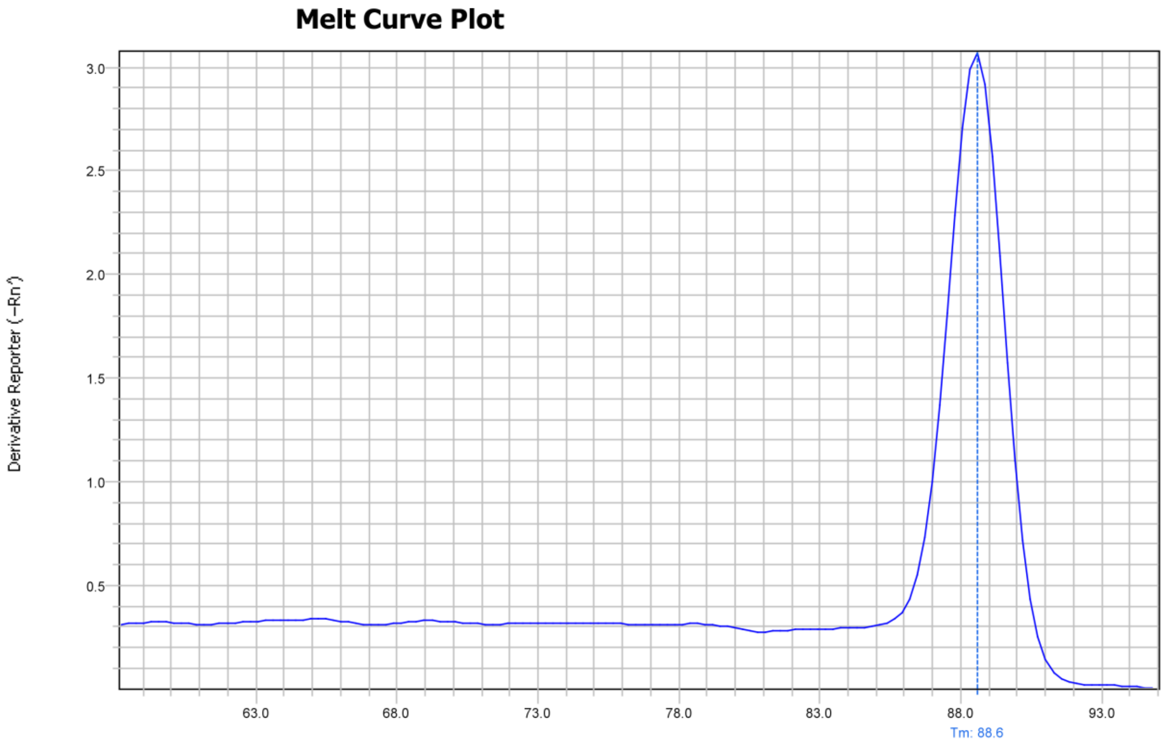

Additional Figure P2. Single, amplicon from *GAPDH* reveals a single peak following melting curve analysis.

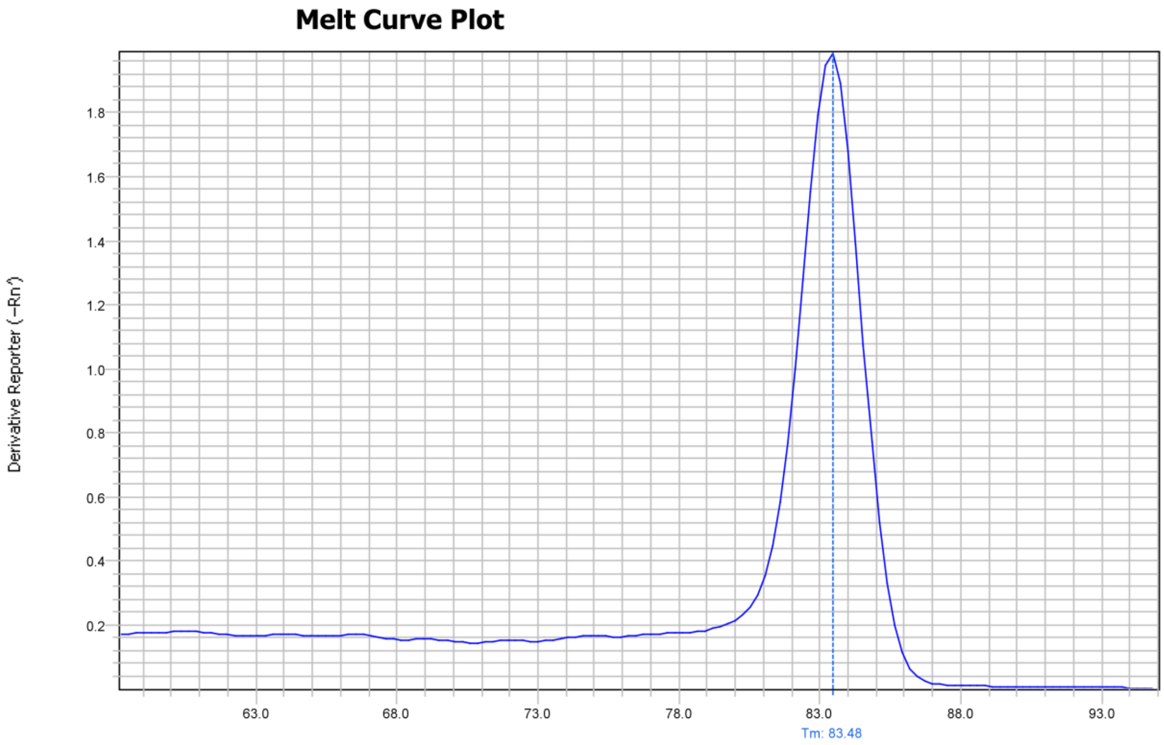

Additional Figure P3. Single, amplicon from *RPL13A* reveals a single peak following melting curve analysis.

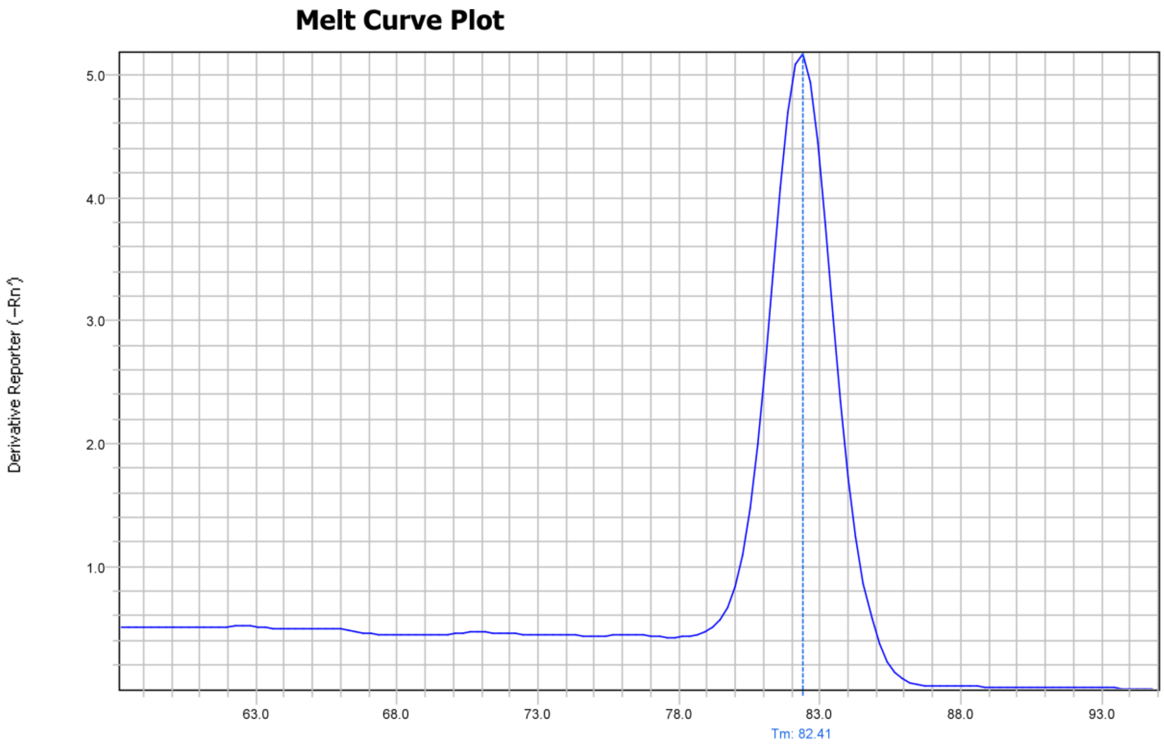

Additional Figure P4. Single, amplicon from *PGK1* reveals a single peak following melting curve analysis.

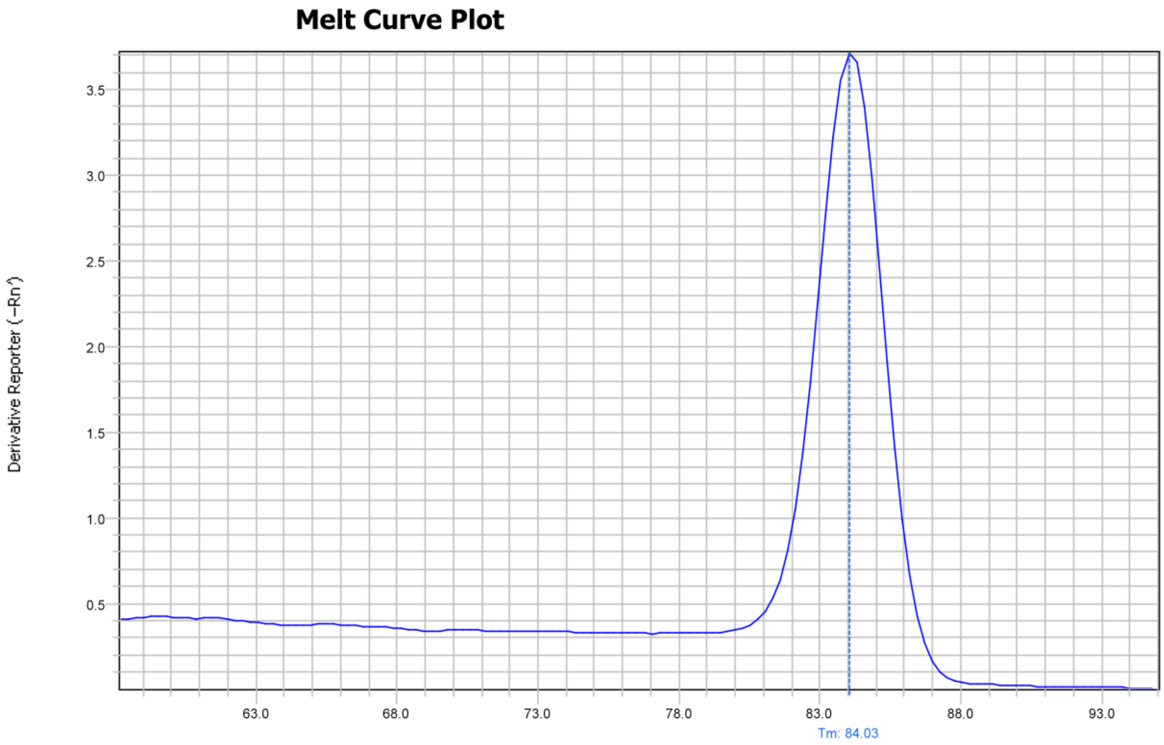

Additional Figure P5. Single, amplicon from *HSPCB* reveals a single peak following melting curve analysis.

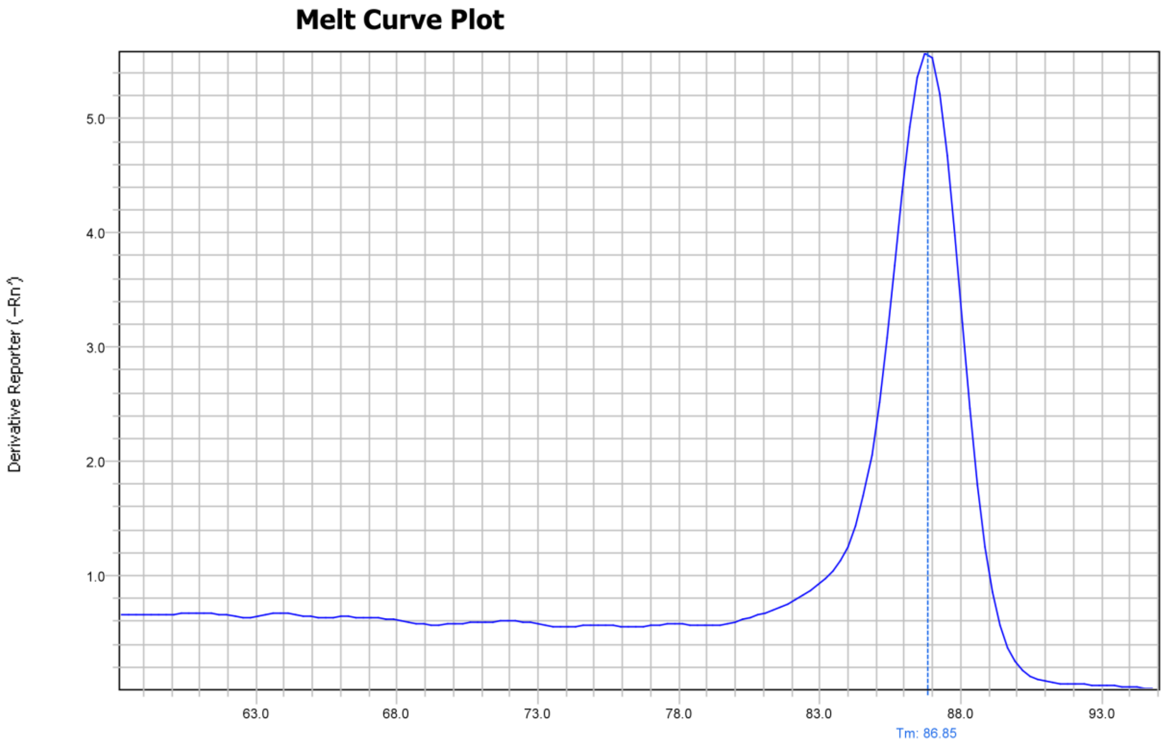

Additional Figure P6. Single, amplicon from *RNA28S* reveals a single peak following melting curve analysis.

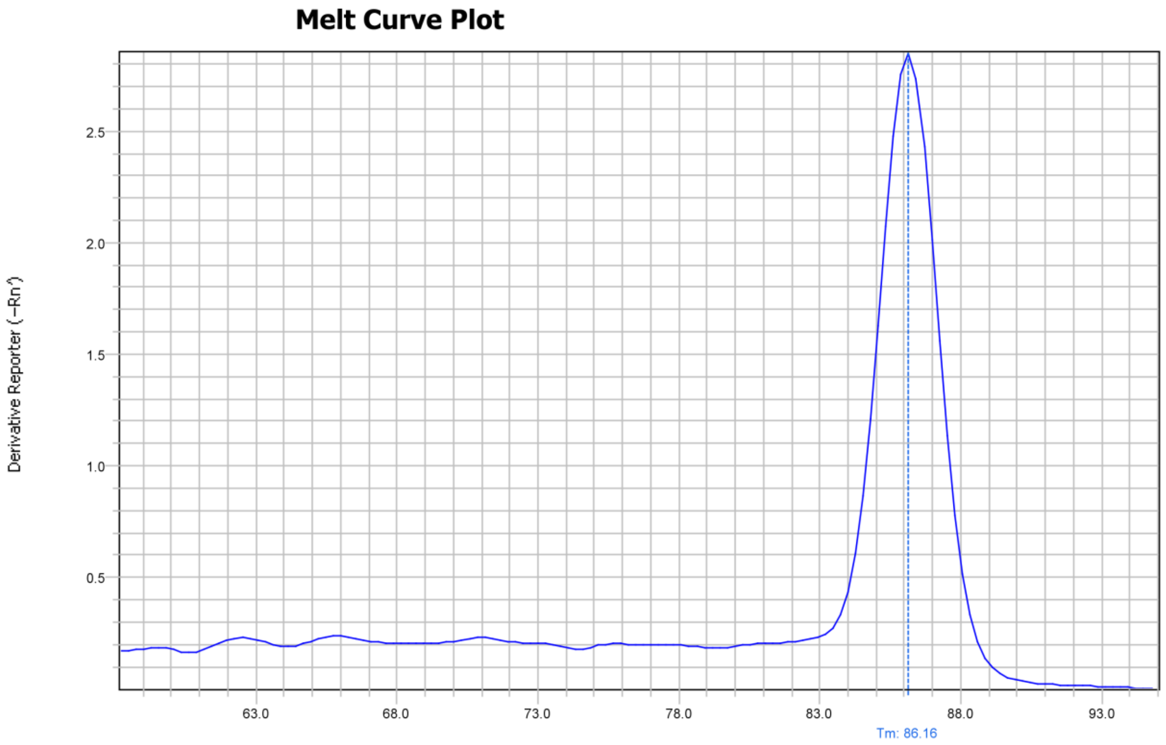

Additional Figure P7. Single, amplicon from *RNA18S* reveals a single peak following melting curve analysis.

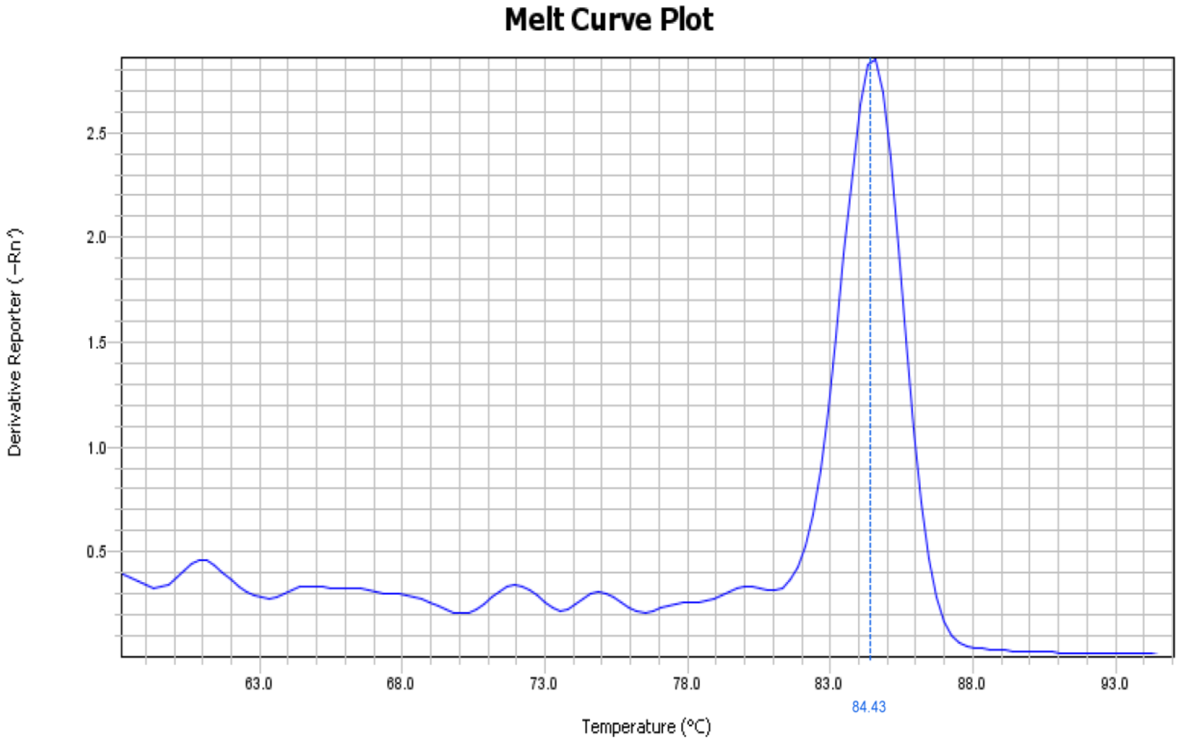

Additional Figure P8. Single, amplicon from *PUM1* reveals a single peak following melting curve analysis.

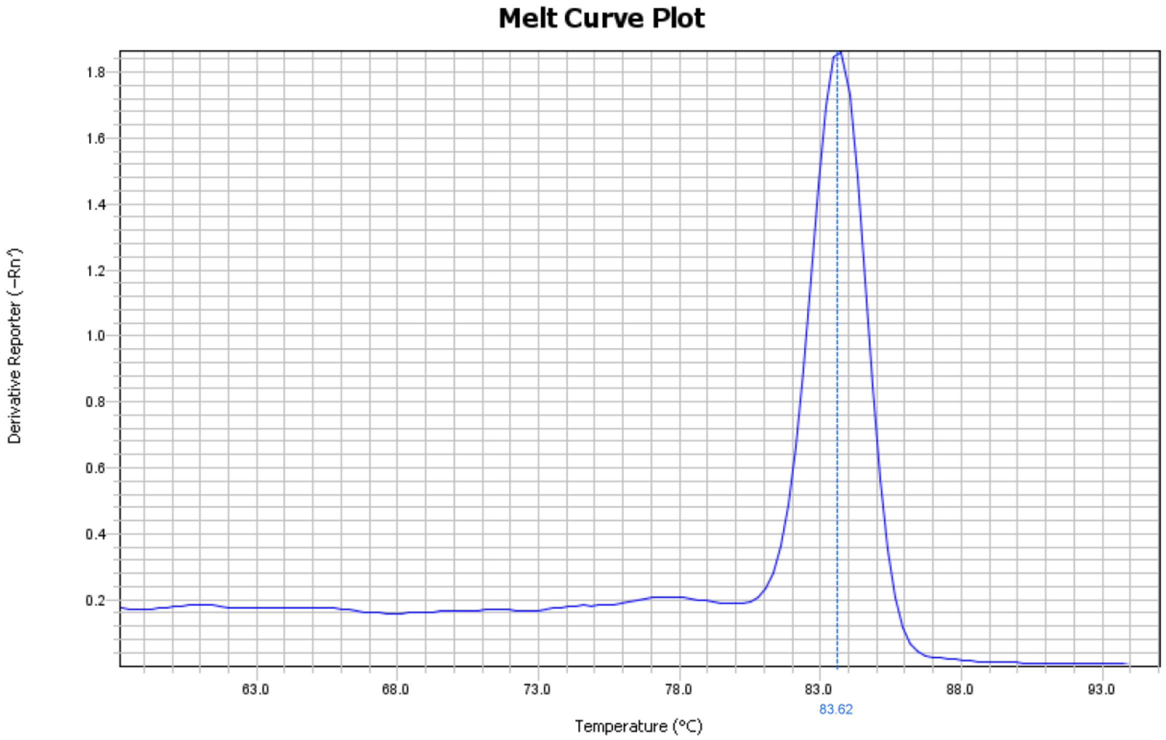

Additional Figure P9. Single, amplicon from *CCSER2* reveals a single peak following melting curve analysis.

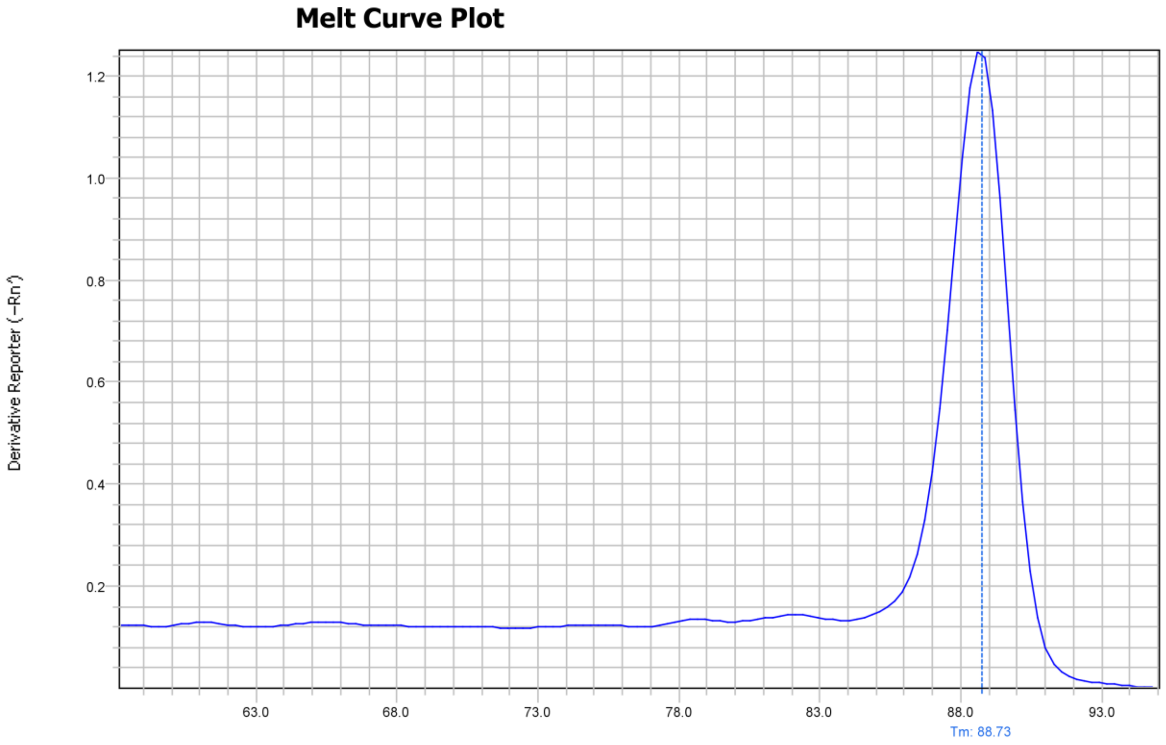

Additional Figure P10. Single, amplicon from *HNRNPL* reveals a single peak following melting curve analysis.

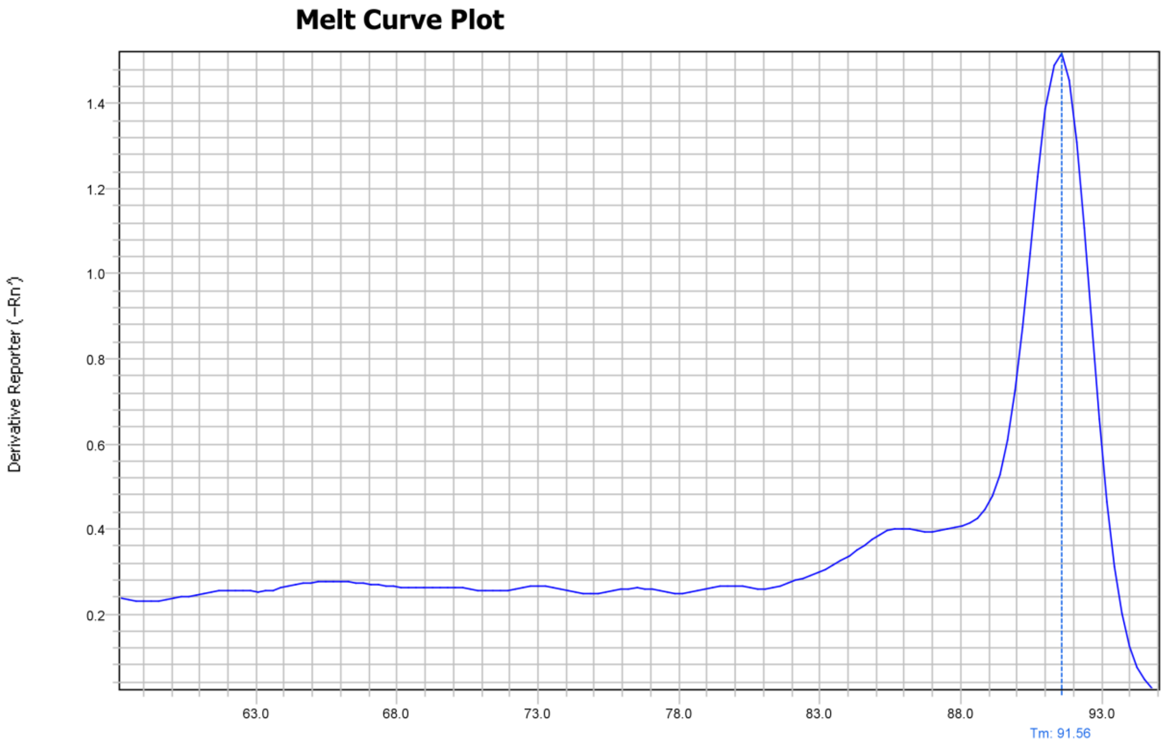

Additional Figure P11. Single, amplicon from *PCBP1* reveals a single peak following melting curve analysis.

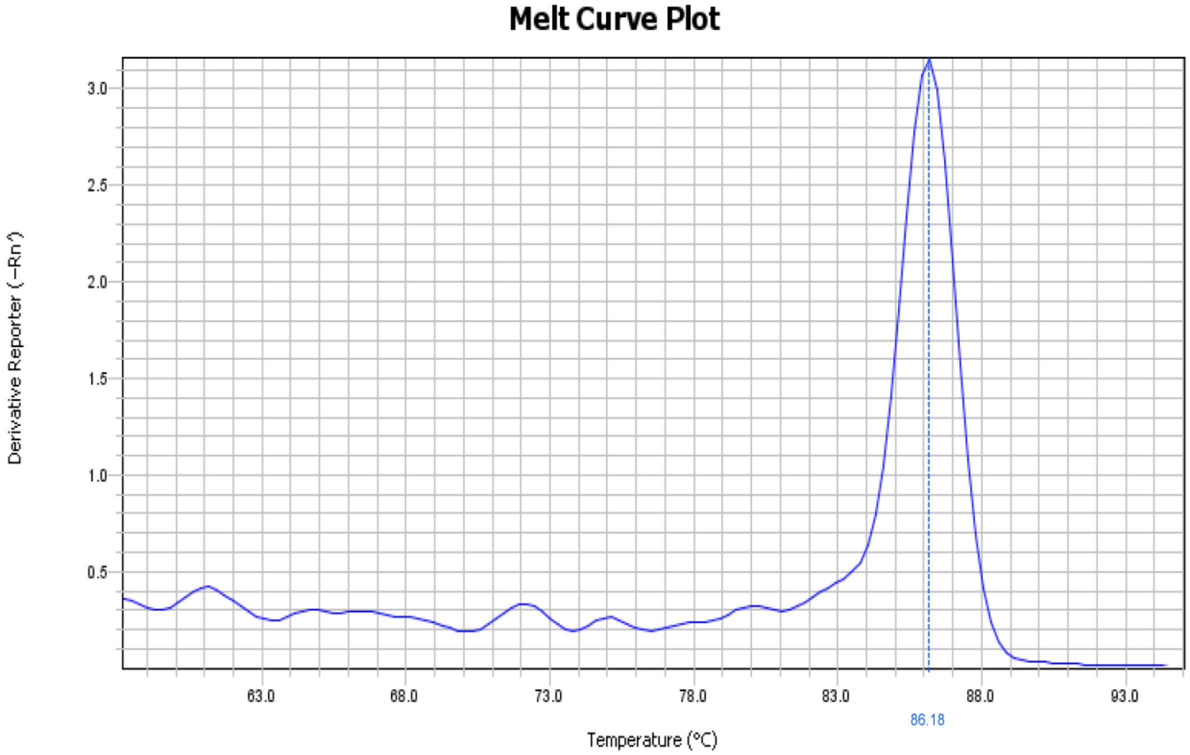

Additional Figure P12. Single, amplicon from *SF3A1* reveals a single peak following melting curve analysis.

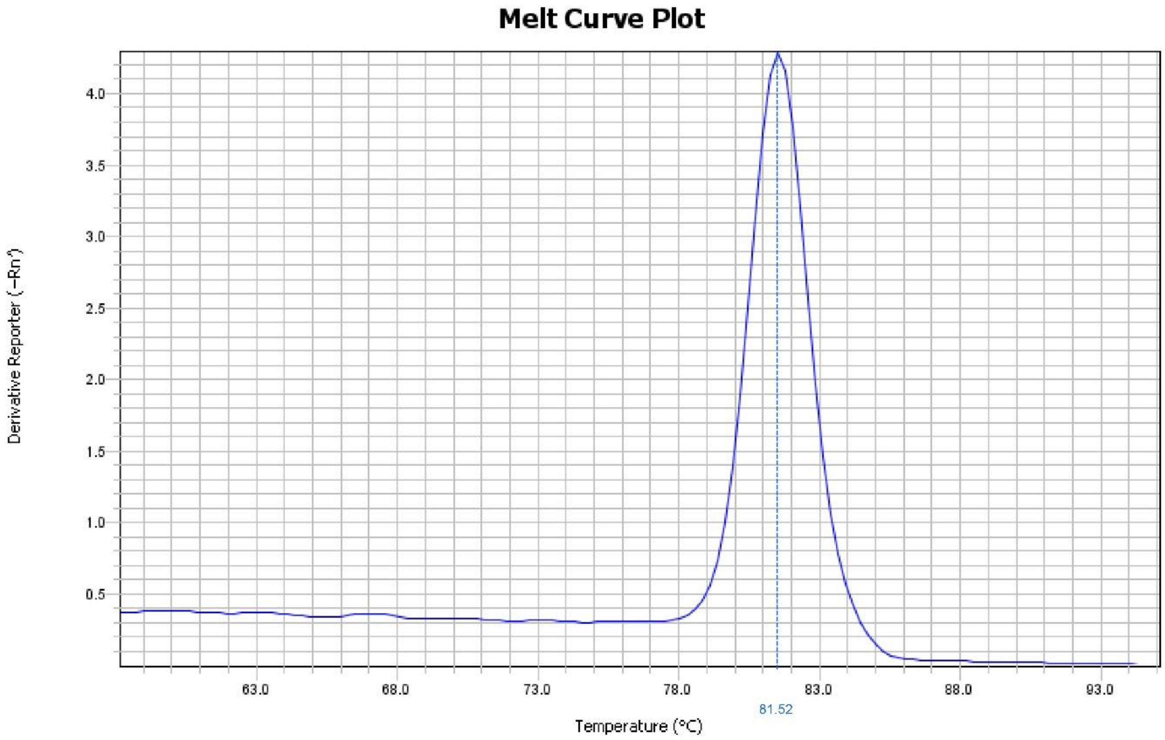

Additional Figure P13. Single, amplicon from *AURKA* reveals a single peak following melting curve analysis.

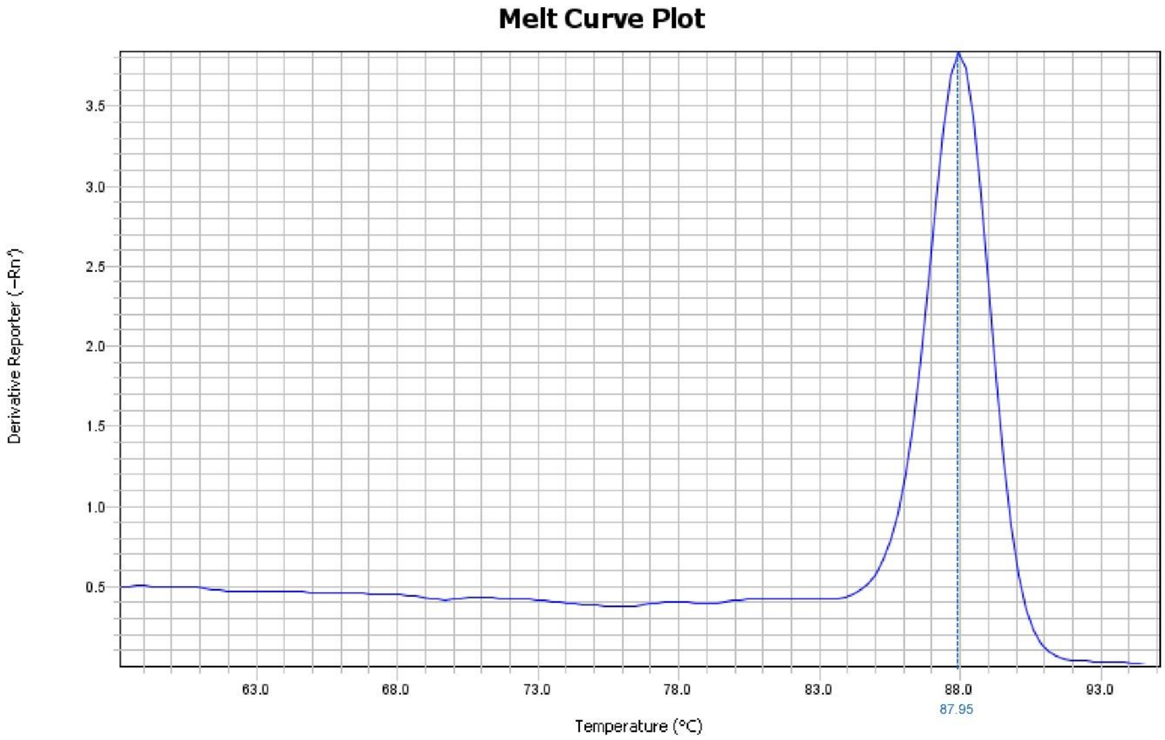

Additional Figure P14. Single, amplicon from *KRT19* reveals a single peak following melting curve analysis.

REFERENCES

1. Suzuki T, Higgins PJ and Crawford DR. Control selection for RNA quantification. Biotechniques. 2000. 29:332-337.
2. Tilli TM, Castro Cda S, Tuszynski JA, Carels N. A strategy to identify housekeeping genes suitable for analysis in breast cancer diseases. BMC Genomics. 2016 Aug. 15;17(1):639. PMID:27526934.
3. Liu LL, Zhao H, Ma TF, Ge F, Chen CS, Zhang YP. Identification of valid reference genes for the normalization of RT-qPCR expression studies in human breast cancer cell lines treated with and without transient transfection. PLoS One. 2015;10(1): e0117058.
4. De Jonge HJM, Fehrmann RSN, De Bont ESJM, Hofstra RMW, Gerbens F, Kamps WA et al. Evidence Based selection of housekeeping genes. PLoS ONE. 2007. 2(9): e898.
5. Jacob F, Guertler R, Naim S, Nixdorf S., Fedier A,,Hacker N.F, Heinzelmann-Schwarz V. Careful Selection of Reference Genes Is Required for Reliable Performance of RT-qPCR in Human Normal and Cancer Cell Lines. 2013. PLoS ONE. 8(3): e59180.
6. Jo, J., Choi, S., Oh, J. et al. Conventionally used reference genes are not outstanding for normalization of gene expression in human cancer research. BMC Bioinformatics. 2019. 20:245.
7. Maltseva DV, Khaustova NA, Fedetov NN, Matveeva EO, Lebedev AE et al. High Throughput identification of reference genes for research and clinical RT-qPCR analysis of breast cancer samples. J Clin Bioinforma. 2013 July. 22;3(1):13. PMID: 23876162.
8. Wang X., Zhou Y., Qiao W., et al. Overexpression of aurora kinase A in mouse mammary epithelium induces genetic instability preceding mammary tumor formation. Oncogene. 2006. 25: 7148-7158.
9. Miyosi Y., Iwao K., Egawa C. and Noguchi S. Association of centrosomal kinase STK15/BTAK mRNA expression with chromosome instability in human breast cancers. Int J Cancer. 2001. 92:370-373.
10. Gritsko T.M., Domenico P., June EP., Lin Y., Mei S., Sue AS., et al. Activation and overexpression of centrosome kinase BTAK/Aurora-A in human ovarian cancer. Clin Cancer. Res. 2003. 9:1420-1426.
11. Li D., Zhu J., Firozi PF., Abbruzzese JL., Evans DB., Cleary K., Friess H. and Sen S. Overexpression of oncogenic STK15/BTAK/Aurora A Kinase in Human pancreatic cancer. Clin Cancer Res. March 2003. 9(3):991-997.
12. Bischoff J.R., Lee A., Yingfang Z., Kevin M., Lelia N., Brian S., Brian S., et al. A homolog of Drosophila aurora kinase is oncogenic and amplified in human colorectal cancers. EMBO Journal. 1998. 17(11):3052-3065.
13. Sen S., Hongyi Z., Ruo-Dan Z., Dong S Y., Funda VL., Shigemi I., et al. Amplification/Overexpression of a mitotic kinase gene in human bladder cancer. J Natl Cancer Inst. 2002. 94:1320-1329.
14. Tong T., Yali Z., Jianping K., Lijia D., Yongmei S., Ming F., Zhihua L., et al. Overexpression of Aurora-A contributes to malignant development of human esophageal squamous cell carcinoma. Clin Cancer Res. 2004. 10(21):7304-7310.
15. Xu J., Wu X., Zhou WH., Liu AW., Wu JB., Deng JY., et al. Aurora A identifies early recurrence and poor prognosis and promises a potential therapeutic target in triple negative breast cancer. PLoS One. 2013. 8(2): e56919.
16. Staff S., Isola J., Jumppanen M. and Tanner M. Aurora-A gene is frequently amplified in basal-like breast cancer. Oncol Rep. 2010. 23:307-312.
17. Ali H.R., Dawson S.J., Blows F.M., Provenzano E., Pharoah P.D. and Caldas C. Aurora Kinase A outperforms Ki67 as a prognostic marker in ER-positive breast cancer. Br J Cancer. 2012. 106:1798-1806.
18. Ignatiadis M., Xenidis N., Perraki M., Apostolaki S., Politaki E., Kafousi M. et al. Different prognostic value of cytokeratin-19 mrna-positive circulating tumor cells according to estrogen receptor and HER2 status in early stage breast cancer. J Clin Oncol. 2007. 25:5194-5202.
19. Wu Y-J. and Rheinwald JG. A new small (40 kd) keratin filament protein made by some cultured human squamous cell carcinomas. Cell. 1981. 25:627-635.
20. Ju J-H., Yang W., Lee K-M., Oh S., Nam K., Shim S., et al. Regulation of cell proliferation and migration by keratin19 induced nuclear import of early growth response-1 in breast cancer cells. Clin Cancer Res. 2013. 19:4335-4346.

21. Saha SK., Choi HY., Kim BW., Dayem AA., Yang GM., Kim KS., Yin YF., and Cho SG. KRT19 directly interacts with β-catenin/RAC1 complex to regulate NUMB-dependent NOTCH signaling pathway and breast cancer properties. Oncogene. Jan 2017. 19;36(3):332-349.
22. Quiroz F.G., Posada O.M., Perez D.G., Castro N.H., Sarassa C., et al. Housekeeping gene stability influences the quantification of osteogenic markers during stem cell differentiation to the osteogenic lineage. Cytotechnology. 2010. 62(2): 109-120.
23. Balwierz A, Czech U, Polus A, Filipkowski R K, et al. Human Adipose tissue stromal vascular fraction cells differentiate depending on distinct types of media. Cell Prolif. 2008. 41:441-459.
24. Kilic Y., Celebiler C. and Sakizli M. Selecting Housekeeping Genes as references for normalization of quantitative PCR data in breast cancer. Published Online May 2013. Clin Transl Oncol. DOI: 10.1007/s12094-013-1058-5.
