# Supplementary material for: Selecting suitable reference genes for qPCR normalization: a comprehensive analysis in MCF-7 breast cancer cell line
Source: BMC Mol Cell Biol. 2020 Sep 25;21:68. doi: 10.1186/s12860-020-00313-x (PMC7519550; doi:10.1186/s12860-020-00313-x)
Supplement: Supplementary file 5 — Additional file 5: Calculations & Algorithms. [file 12860_2020_313_MOESM5_ESM.pdf]

ADDITIONAL FILE 5: CALCULATIONS & ALGORITHMS

Selecting Suitable Reference Genes for qPCR Normalization: A Comprehensive Analysis in MCF-7 Breast Cancer Cell Line

Authors: Nityanand Jain, Dina Nitisa, Valdis Pirsko and Inese Cakstina*

*** For Correspondence:**

Laboratory of Molecular Genetics
Institute of Oncology
Riga Stradins University
16 Dzirciema street
Riga
Latvia (LV-1007)

Email: inese.cakstina@rsu.lv

INDEX

1) Additional Table 1 – Description of various algorithms (Section 5.6)
2) Calculations for *Sections 2.3, 2.10, 2.11, 2.15* and *2.16*
3) References

**Abbreviations – p - passage; RG - reference gene; GOI – gene of interest

Additional Table 1. Description and criteria for different algorithms/approaches used in the present study to determine most stable reference gene pair

Algorithm/ Approach	Section in Study	Criteria	Notes	Reference
Coefficient of Variance (CV%)	*Section 2.3*	Homogenous sample CV < 25% and Heterogenous sample CV < 50%	Also known as Relative Standard Deviation, is calculated as the ratio of the standard deviation and mean of linear Cq values (2^{-Cq}). A lower CV% indicates higher gene expression stability and visa versa.	1,2,3
NormFinder	*Section 2.4*	Lower the group standard deviation, higher the expression stability of the gene	Estimates the overall variation in gene expression for each gene and delivers a stability value that identifies not only the most stable gene but also the best control gene. Stability of gene is presented as an estimate of combined intra- and inter-group variation of individual gene.	4
geNorm	*Section 2.5*	Lower the M value, more stable the gene; M < 1 for heterogenous tissue like breast cancer	Calculates M value for each candidate gene based on pairwise comparisons. Uses stepwise exclusion method of the least stable genes by calculating average M values.	5
		Vn/Vn+1 < 0.15	geNorm can also estimate optimal number of genes required for accurate normalization of expression data. Vn represents the number of genes suitable for normalization. However, in case Vn = 2, it is recommended to consider three stable internal controls.	
BestKeeper	*Section 2.6*	Any of three criteria: 1) S.D ± C.P ≤ 1 2) S.D. ± x-fold ≤ 2 3) High coefficient of correlation r	Analyzes the expression stability of reference genes using crossing points (C.P) to decide whether the genes are differentially expressed under the applied conditions or not.	6

Comparative ΔCt	*Section 2.7*	Lower the standard deviation, higher the expression stability of the gene	Compares the relative expression of pairs of candidate reference genes within each sample to identify and rank the most stable genes. For a stable gene, it should be strongly expressed, displays minimum fluctuations and is independent of expression of other genes.<br><br>Further, if ΔCq values of two genes fluctuate when analyzed in different samples, it can be concluded that one or both genes are variably expressed. Conversely, if the ΔCq values remain constant, then both genes are either expressed stably or are co-regulated among the samples.	7
RefFinder	*Section 2.8*	Lower the geometric mean, the higher the expression stability of the gene	Measures geometric mean of attributed weights by NormFinder, geNorm, BestKeeper and Comparative ΔCt to generate an overall final ranking.	8

CALCULATIONS FOR *SECTION 2.3* (Relative Mean Changes in Expression Profiles)

To calculate relative mean changes with increasing passages (compared with an internal calibrator passage), we used $2^{-\Delta Cq}$ method to calculate fold change [9].

1) Transform the raw Cq values to linear scale using 2^{-Cq} for both internal calibrator passage and passage of interest.
2) Calculate mean 2^{-Cq} for the internal calibrator passage.
3) Divide each individual triplicate Cq value with mean 2^{-Cq} to get the relative mean change in expression profile (relative fold change to internal calibrator).
4) Plot the values on boxplot for better visualization and use *ANOVA* for *P* value (for 3 or more passages).

An example for relative mean changes in expression profile of *RNA18S* in p32 when compared with the internal calibrator p28 from Culture A1 (from the present study):

Triplicates	p28 Cq values	p32 Cq values	2^{-Cq} for p28 Cq values	2^{-Cq} for p32 Cq values	Relative change in p28	Relative change in p32
Sample 1.1	8.153	7.825	3.51E-03	4.41E-03	0.962	1.207
Sample 1.2	8.072	7.938	3.72E-03	4.08E-03	1.017	1.116
Sample 1.3	8.033	7.910	3.82E-03	4.16E-03	1.045	1.138
Sample 2.1	8.206	7.446	3.39E-03	5.73E-03	0.927	1.570
Sample 2.2	8.081	7.477	3.69E-03	5.61E-03	1.011	1.537
Sample 2.3	8.044	7.477	3.79E-03	5.61E-03	1.037	1.537
Sample 3.1	-	7.610	-	5.12E-03	-	1.401
Sample 3.2	-	7.615	-	5.10E-03	-	1.397
Sample 3.3	-	7.629	-	5.05E-03	-	1.383

Use this value to divide 2^{-Cq} values to get relative change → **3.65E-03** (Mean)

Plot on boxplot and calculate *ANOVA* for P values

** Note that in the present study there were more than 3 passages in each culture and hence we used *ANOVA* with post-hoc and Bonferroni correction to calculate *P* values. In case your experiment has 2 passages, use Student's t test (or non-parametric Mann-Whitney *U* test).

CALCULATIONS FOR *SECTIONS 2.10* and *2.11* (Gene of Interest Normalization by Reference Gene)

To normalize the expression of gene of interest using reference gene, we used the ΔΔCt method [10]. However, the method described by Livak [10] can only be used in case of normalization by a single gene. An extension to this method has been suggested, where the average expression of all reference genes can be used [11].

1) Calculate the mean/ average for gene of interest and reference genes.
2) For multiple reference genes, use Cq average of the reference genes.
3) Calculate mean ΔCt by subtracting mean Cq of GOI and reference gene.
4) Repeat Steps 1-3 for both internal calibrator passage and passage of normalization.
5) Calculate ΔΔCt by subtracting the mean ΔCt from internal calibrator passage and passage of normalization.
6) Convert the ΔΔCt into linear scale by using $2^{-\Delta\Delta Ct}$.
7) For calculating *P* value, use individual ΔCt values and perform *ANOVA* (for 3 or more passages).

An example for *AURKA* normalization by *GAPDH-PCBP1-CCSER2* in p25/30 in Culture A2 is shown below (from the present study):

Triplicates	p25 Cq values for *AURKA*	p25 Cq values for RGs	ΔCt	p30 Cq values for *AURKA*	p30 Cq values for RGs	ΔCt
Sample 1.1	23.512	21.578	1.934	23.697	22.159	1.538
Sample 1.2	23.517	21.519	1.998	23.742	22.016	1.726
Sample 1.3	23.485	21.543	1.942	23.676	22.089	1.586
Sample 2.1	23.494	21.621	1.873	24.074	22.161	1.913
Sample 2.2	23.414	21.671	1.743	24.025	22.088	1.937
Sample 2.3	23.223	21.599	1.624	24.006	22.072	1.934
Sample 3.1	23.248	21.639	1.609	23.759	21.748	2.011
Sample 3.2	23.241	21.581	1.660	23.700	21.732	1.968
Sample 3.3	23.328	21.599	1.729	23.414	21.799	1.615

Mean/Average: 23.385 21.595 23.788 21.985

Average ΔCt: 1.790 1.803

Average ΔΔCt: - 0.013

$2^{-\Delta\Delta Ct}$: 1.009

P value: 0.436 (not significant at $P < 0.05$)

Successful Normalization !!

To calculate the Cq values for multiple Reference Genes (2 or more), we can analyze the *GAPDH-PCBP1-CCSER2* triplet from the above example for p25 and p30 in Culture A2 (from the present study):

Triplicates	p25 Cq values (*GAPDH*)	p25 Cq values (*PCBP1*)	p25 Cq values (*CCSER2*)	p30 Cq values (*GAPDH*)	p30 Cq values (*PCBP1*)	p30 Cq values (*CCSER2*)
Sample 1.1	16.697	21.683	26.353	17.585	22.225	26.667
Sample 1.2	16.681	21.766	26.111	17.507	22.165	26.375
Sample 1.3	16.700	21.813	26.117	17.639	22.058	26.572
Sample 2.1	16.778	21.771	26.314	17.263	22.444	26.775
Sample 2.2	16.785	21.924	26.304	17.248	22.377	26.640
Sample 2.3	16.755	21.807	26.235	17.275	22.295	26.647
Sample 3.1	16.817	21.797	26.303	17.047	22.097	26.100
Sample 3.2	16.804	21.785	26.155	17.278	21.853	26.065
Sample 3.3	16.845	21.804	26.148	17.176	21.811	26.410

MEAN / AVERAGE

	p25	USED FOR $2^{-\Delta\Delta Ct}$ CALCULATIONS	p30
Sample 1.1	21.578		22.159
Sample 1.2	21.519		22.016
Sample 1.3	21.543		22.089
Sample 2.1	21.621		22.161
Sample 2.2	21.671		22.088
Sample 2.3	21.599		22.072
Sample 3.1	21.639		21.748
Sample 3.2	21.581		21.732
Sample 3.3	21.599		21.799

** Note that in the present study there were more than 3 passages in each culture and hence we used *ANOVA* with post-hoc and Bonferroni correction to calculate *P* values. In case your experiment has 2 passages, use Student's t test (or non-parametric Mann-Whitney *U* test).

CALCULATIONS FOR *SECTION 2.15* (Fold change in Reference Gene expression)

To calculate the fold change in reference gene expression (nutrient stress vs control cultures), we used the 2^{-Cq} method as described by Schmittgen and Livak [9] in example 3 of their publication.

1) Convert raw Cq values (in triplicates) from RT-qPCR to linear scale by using the formula 2^{-Cq} for both control and nutrient stress cultures.
2) Calculate the mean value for all lysates from both cultures.
3) Divide the mean values so obtained in Step 2.
4) Finally, divide 1 with the value from Step 3 to obtain change in expression fold change.

An example for fold change in expression of *RPL13A* gene in nutrient stress (R5) vs control culture (A2-p25) is shown below (from the present study):

	**Nutrient Stress Culture (R5)**	**2^{-Cq} Values**	**Control Culture (A2-p25)**	**2^{-Cq} Values**
Sample 1.1	20.486	6.81E-07	20.397	7.24E-07
Sample 1.2	20.503	6.73E-07	20.359	7.44E-07
Sample 1.3	20.550	6.51E-07	20.389	7.28E-07
Sample 2.1	20.685	5.93E-07	20.529	6.61E-07
Sample 2.2	20.892	5.14E-07	20.557	6.48E-07
Sample 2.3	21.318	3.83E-07	20.472	6.88E-07
Sample 3.1	-	-	20.610	6.25E-07
Sample 3.2	-	-	20.564	6.45E-07
Sample 3.3	-	-	20.550	6.51E-07
	**Mean/Average:**	5.82E-07		6.79E-07

Triplicates

Fold change = 5.82E-07 / 6.79E-07 = 0.857

Expression change = 1 / 0.857 = 1.166 = 1.17 (round-off)

Interpretation: Nutrient stress in MCF-7 cells grown in R5 culturing conditions reduced the expression of *RPL13A* by 1.17x fold when compared with MCF-7 cells grown in routine standard culturing conditions (culture A2).

Suggested Cutoff: An expression change of ≥ 2 is considered as cutoff for determining whether the reference gene is a good internal control or not.

CALCULATIONS FOR *SECTION 2.16* (Normalized Expression Differences of GOI)

To calculate the normalized expression difference of GOI in nutrient stress vs control conditions, we used the $2^{-\Delta Cq}$ method as described by Schmittgen and Livak [9] in example 5 of their publication.

1) Calculate the mean/average Cq values for GOI and multiple reference genes (as explained above) for nutrient stress condition.
2) Repeat the same for control culture.
3) Calculate mean $2^{-\Delta Cq}$ using the means obtained in step 1 and 2 for both control and nutrient stress cultures.
4) Divide the $2^{-\Delta Cq}$ values.
5) Divide -1 by the value obtained in Step 4.
6) Calculate *P* value by calculating individual sample $2^{-\Delta Cq}$ values and use non-parametric Mann-Whitney *U* test.

An example for *KRT19* normalization by *GAPDH-PCBP1-CCSER2* in nutrient stress culture B5 by control culture A2 with internal calibrator passage p25 is shown below (from the present study):

Triplicates	Cq values for *KRT19* in B5	Cq values for RGs in B5	$2^{-\Delta Cq}$	p25 Cq values for *KRT19*	p25 Cq values for RGs	$2^{-\Delta Cq}$
Sample 1.1	19.693	21.998	4.943	18.700	21.578	7.350
Sample 1.2	19.757	22.023	4.810	18.829	21.519	6.455
Sample 1.3	19.733	22.153	5.353	18.650	21.543	7.430
Sample 2.1	19.593	22.205	6.115	18.775	21.621	7.190
Sample 2.2	19.651	22.086	5.408	18.791	21.671	7.361
Sample 2.3	19.624	22.391	6.805	18.661	21.599	7.663
Sample 3.1	-	-	-	18.789	21.639	7.210
Sample 3.2	-	-	-	18.671	21.581	7.518
Sample 3.3	-	-	-	18.698	21.599	7.469
**Mean/Average:**	19.675	22.143		18.729	21.595	
**Average $2^{-\Delta Cq}$:**	5.531			7.286		

Expression fold change: 0.759

Expression change due to stress: - 1.317

P value (Mann Whitney): 0.428 (not significant at $P < 0.05$)

Successful Normalization !!

Interpretation: Normalized fold change in *KRT19* expression due to nutrient stress in MCF-7 cells cultured as culture B5 is -1.317-fold when compared with MCF-7 cells cultured in routine standard conditions (Culture A2).

Suggested Cutoff: An expression change of ≥ 2 is considered as cutoff. Further, we suggest to evaluate the Mann-Whitney test *P* value to statistically validate the significance of fold change in expression.

REFERENCES

1. Venkat K.S, Nirmal K.S., Charbel M., Julien G. Optimal Use of statistical methods to validate reference gene stability in longitudinal studies. Plos One. 2019. 14(7): e0219440.
2. Jan Hellemans, Geert Mortier, Anne De Paepe, Frank Speleman, Jo Vandesompele. qBase relative quantification framework and software for management and automated analysis of real-time quantitative PCR data. Genome Biology. 2007 Feb. 8(2): R19.
3. D'haene B., Hellemans J. The importance of quality control during qPCR data analyis. Int. Drug Discov. 2010; 18–24.
4. Andersen CL, Jensen JL, Orntoft TF. Normalization of real-time quantitative reverse transcription-PCR data: a model-based variance estimation approach to identify genes suited for normalization, applied to bladder and colon cancer data sets. Cancer research. 2004. 64:5245-5250.
5. Vandesompele J, De Preter K, Pattyn F, Poppe B, Van Roy N, et al. Accurate normalization of real time quantitative RT-PCR data by geometric averaging of multiple internal control genes. Genome Biol 3: Research0034. 2002.
6. Pfaffl MW, Tichopad A, Prgomet C, Neuvians TP. Determination of stable housekeeping genes, differentially regulated target genes and sample integrity: BestKeeper--Excel-based tool using pair-wise correlations. Biotechnology letters. 2004. 26:509-515.
7. Silver N, Best S, Jiang J, Thein SL. Selection of housekeeping genes for gene expression studies in human reticulocytes using real-time PCR. BMC molecular biology. 2006. 7:33.
8. F Xie, P Xiao, D Chen, L Xu, B Zhang. miRDeepFinder: a miRNA analysis tool for deep sequencing of plant small RNAs. Plant molecular biology. 2012. 80 (1), 75-84.
9. Schmittgen, T. D., & Livak, K. J. Analyzing real-time PCR data by the comparative CT method. Nature Protocols. 2008; 3(6): 1101–1108. doi:10.1038/nprot.2008.73.
10. Livak K-J. and Schmittgen T-D. Analysis of Relative gene expression data using real-time quantitative PCR and the $2^{-\Delta\Delta CT}$ method. Methods. 2001. 25; 402-408. DOI: 10.1006/meth.2001.1262.
11. Riedel G, Rüdrich U, Fekete-Drimusz N, Manns MP, Vondran FW, Bock M. An extended ΔCT-method facilitating normalisation with multiple reference genes suited for quantitative RT-PCR analyses of human hepatocyte-like cells. PLoS One. 2014 Mar 21;9(3):e93031. doi: 10.1371/journal.pone.0093031. PMID: 24658132; PMCID: PMC3962476.
