# Supplementary material for: Selecting suitable reference genes for qPCR normalization: a comprehensive analysis in MCF-7 breast cancer cell line
Source: BMC Mol Cell Biol. 2020 Sep 25;21:68. doi: 10.1186/s12860-020-00313-x (PMC7519550; doi:10.1186/s12860-020-00313-x)
Supplement: Supplementary file 6 — Additional file 6: Control & Stress Media. [file 12860_2020_313_MOESM6_ESM.pdf]

ADDITIONAL FILE 6: CONTROL & STRESS MEDIA

Selecting Suitable Reference Genes for qPCR Normalization: A Comprehensive Analysis in MCF-7 Breast Cancer Cell Line

Authors: Nityanand Jain, Dina Nitisa, Valdis Pirsko and Inese Cakstina*

As Adapted & Modified from (our previous study):

Pirsko V. et al. An Effect of Culture Media on Epithelial Differentiation Markers in Breast Cancer Cell Lines MCF7, MDA-MB-436 and SkBr3. Medicina (Kaunas). 2018 Mar 30;54(2):11.

*** For Correspondence:**

Laboratory of Molecular Genetics
Institute of Oncology
Riga Stradins University
16 Dzirciema street
Riga
Latvia (LV-1007)

Email: inese.cakstina@rsu.lv

INDEX

1) Additional Table 1 - Growth media compositions
2) Additional Table 2 – Reagents

Additional Table 1. Composition of growth media in control (A1 and A2) and nutrient stress (B5, D5, E5 and R5) cultures

**Basal Media & Supplements**	**Control Cultures**	**Nutrient Stress Cultures**			
	**A1/A2**	**B5**	**D5**	**E5**	**R5**
Basal Culture Media					
DMEM:F12	✓ (1:1)		✓ (1:3)	✓ (1:1)	
M199		✓			
RPMI-1640					✓
FBS (%)	10	5	5	5	5
Supplements (%)					
Pen/Strep	1	1	1	1	1
Insulin (I)		0.05	0.05	0.05	0.05
Hydrocortisone		0.1	0.1	0.1	0.1
Epidermal G.F.		0.1	0.1	0.1	0.1
17β – Estradiol		0.1	0.1	0.1	0.1
HEPES		1	1	1	
Transferrin		0.1	0.1	0.1	0.1
T3		0.033	0.033	0.033	0.033
Se		0.044	0.044	0.044	0.044
Cholera toxin		0.1	0.1	0.1	0.1

*DMEM:F12 – Dulbecco's Modified Eagle's Medium with Ham's F12 nutrient supplement; M199 – Medium 199; RPMI-1640 - Roswell Park Memorial Institute medium; FBS – Fetal Bovine Serum; Pen/Strep – Penicillin/Streptomycin; Epidermal G.F. – Epidermal Growth Factor; HEPES - (4-(2-hydroxyethyl)-1-piperazineethanesulfonic acid); T3 - 3,3′,5-triiodo-l-thyronine; Se—sodium selenite.

Additional Table 2. List of reagents and manufacturers

Reagent	Manufacturer	Country	Product No./ Catalogue No.
DMEM/F12, GlutaMAX	ThermoFisher Scientific	United States	31331028
Medium 199 (M199)	Sigma-Aldrich	Germany	M4530
RPMI-1640	ThermoFisher Scientific	United States	A1049101
FBS	Sigma-Aldrich	Germany	F9665
Pen/Strep	Sigma-Aldrich	Germany	P4333
Insulin (I)	Sigma-Aldrich	Germany	I0516
Hydrocortisone	Sigma-Aldrich	Germany	H0888
Epidermal G.F.	ThermoFisher Scientific	United States	PHG0313
17β – Estradiol	Cayman Chemical	United States	10006315
HEPES	AppliChem	Germany	A3268
Transferrin	Sigma-Aldrich	Germany	T8158
T3	Sigma-Aldrich	Germany	T6397
Se	Sigma-Aldrich	Germany	S9133
Cholera toxin	Sigma-Aldrich	Germany	C9903
TrypLE	ThermoFisher Scientific	United States	A12177-02
Trizol Reagent	ThermoFisher Scientific	United States	15596026
High Capacity cDNA Reverse Transcription kit	ThermoFisher Scientific	United States	4368814
HOT FIREPol EvaGreen qPCR Supermix	Solis Biodyne	Estonia	08-36-00008
FIREPol DNA Polymerase	Solis Biodyne	Estonia	01-01-00002

*DMEM:F12 – Dulbecco's Modified Eagle's Medium with Ham's F12 nutrient supplement; M199 – Medium 199; RPMI-1640 - Roswell Park Memorial Institute medium; FBS – Fetal Bovine Serum; Pen/Strep – Penicillin/Streptomycin; Epidermal G.F. – Epidermal Growth Factor; HEPES - (4-(2-hydroxyethyl)-1-piperazineethanesulfonic acid); T3 - 3,3',5-triiodo-l-thyronine; Se—sodium selenite.
